# Timing of Decompressive Surgery in Patients With Acute Spinal Cord Injury: Systematic Review Update

**DOI:** 10.1177/21925682231197404

**Published:** 2024-03-25

**Authors:** Michael G. Fehlings, Laureen D. Hachem, Lindsay A. Tetreault, Andrea C. Skelly, Joseph R. Dettori, Erika D. Brodt, Shay Stabler-Morris, Britt J. Redick, Nathan Evaniew, Allan R. Martin, Benjamin Davies, Farzin Farahbakhsh, James D. Guest, Daniel Graves, Radha Korupolu, Stephen L. McKenna, Brian K. Kwon

**Affiliations:** 1 Division of Neurosurgery and Spine Program, Department of Surgery, 7938 University of Toronto, Toronto, Ontario, Canada; 2 Division of Neurosurgery, Krembil Neuroscience Centre, Toronto Western Hospital, 7989 University Health Network, Toronto, Ontario, Canada; 3 Institute of Medical Science, 7938 University of Toronto, Toronto, Ontario, Canada; 4 Department of Neurology, 12297 NYU Langone Medical Center, New York, NY, USA; 5 Aggregate Analytics, Inc, Fircrest, WA, USA; 6 Spectrum Research, Inc, Steilacoom, WA, USA; 7 McCaig Institute for Bone and Joint Health, Department of Surgery, Orthopaedic Surgery, Cumming School of Medicine, 2129 University of Calgary, AB, Canada; 8 Department of Neurological Surgery, University of California, Davis, CA, USA; 9 Department of Neurosurgery, 2152 Cambridge University, Cambridge, UK; 10 Department of Neurosurgery, Shariati Hospital, Tehran University of Medical Sciences, Tehran, Iran; 11 Department of Neurosurgery and The Miami Project to Cure Paralysis, 12235 University of Miami Miller School of Medicine, Miami, FL, USA; 12 Department of Rehabilitation Medicine, 6559 Sidney Kimmel Medical College at Thomas Jefferson University, Philadelphia, PA, USA; 13 Department of Physical Medicine and Rehabilitation, 12340 University of Texas Health Science Center at Houston, Houston, TX, USA; 14 Department of Neurosurgery, 6429 Stanford University, Stanford, CA, USA; 15 Department of Orthopaedics, 8166 University of British Columbia, Vancouver, BC, Canada; 16 International Collaboration on Repair Discoveries (ICORD), 8166 University of British Columbia, Vancouver, British Columbia, Canada

**Keywords:** spinal cord injury, trauma, surgical decompression

## Abstract

**Study design:**

Systematic review and meta-analysis.

**Objective:**

Surgical decompression is a cornerstone in the management of patients with traumatic spinal cord injury (SCI); however, the influence of the timing of surgery on neurological recovery after acute SCI remains controversial. This systematic review aims to summarize current evidence on the effectiveness, safety, and cost-effectiveness of early (≤24 hours) or late (>24 hours) surgery in patients with acute traumatic SCI for all levels of the spine. Furthermore, this systematic review aims to evaluate the evidence with respect to the impact of ultra-early surgery (earlier than 24 hours from injury) on these outcomes.

**Methods:**

A systematic search of the literature was performed using the MEDLINE database (PubMed), Cochrane database, and EMBASE. Two reviewers independently screened the citations from the search to determine whether an article satisfied predefined inclusion and exclusion criteria. For all key questions, we focused on primary studies with the least potential for bias and those that controlled for baseline neurological status and specified time from injury to surgery. Risk of bias of each article was assessed using standardized tools based on study design. Finally, the overall strength of evidence for the primary outcomes was assessed using the GRADE approach. Data were synthesized both qualitatively and quantitively using meta-analyses.

**Results:**

Twenty-one studies met inclusion and exclusion criteria and formed the evidence base for this review update. Seventeen studies compared outcomes between patients treated with early (≤24 hours from injury) compared to late (>24 hours) surgical decompression. An additional 4 studies evaluated even earlier time frames: <4, <5, <8 or <12 hours. Based on moderate evidence, patients were 2 times more likely to recover by ≥ 2 grades on the ASIA Impairment Score (AIS) at 6 months (RR: 2.76, 95% CI 1.60 to 4.98) and 12 months (RR: 1.95, 95% CI 1.26 to 3.18) if they were decompressed within 24 hours compared to after 24 hours. Furthermore, moderate evidence suggested that patients receiving early decompression had an additional 4.50 (95% CI 1.70 to 7.29) point improvement on the ASIA motor score. With respect to administrative outcomes, there was low evidence that early decompression may decrease acute hospital length of stay. In terms of safety, there was moderate evidence that suggested the rate of major complications does not differ between patients undergoing early compared to late surgery. Furthermore, there was no difference in rates of mortality, surgical device-related complications, sepsis/systemic infection or neurological deterioration based on timing of surgery. Firm conclusions were not possible with respect to the impact of ultra-early surgery on neurological, functional or safety outcomes given the poor-quality studies, imprecision and the overlap in the time frames examined.

**Conclusions:**

This review provides an evidence base to support the update on clinical practice guidelines related to the timing of surgical decompression in acute SCI. Overall, the strength of evidence was moderate that early surgery (≤24 hours from injury) compared to late (>24 hours) results in clinically meaningful improvements in neurological recovery. Further studies are required to delineate the role of ultra-early surgery in patients with acute SCI.

## Introduction

Traumatic spinal cord injury (SCI) leads to permanent sensorimotor impairment, decreased quality of life, and immense social as well as economic costs. Currently, there remains no effective regenerative or neuroprotective treatment for SCI. Therefore, it is important to optimize post-SCI interventions to maximize recovery. Surgical decompression is a cornerstone in the management of patients with SCI; however, the influence of the timing of surgery on neurological recovery after acute SCI remains controversial. Early pre-clinical studies demonstrated a significant benefit of early surgical decompression in improving functional outcomes in animal models of SCI.^1^ In recent years, clinical evidence has emerged in support of early surgery for acute SCI; however, clinical practice patterns remain variable. A 2017 systematic review^2^ and related clinical practice guidelines^3^ suggested that early surgical management (within 24 hours) of acute SCI may facilitate improvement in neurological function; however, the confidence in the evidence was low to very low for primary outcomes. After the publication of these guidelines, numerous additional studies on the effectiveness and safety of early surgical decompression have emerged,^4-13^ including specific research on time frames for surgical management of less than 24 hours. As such, there is a strong need to re-examine the current evidence surrounding the role of surgical timing for SCI.

This systematic review update aims to critically appraise and summarize current evidence on the effectiveness, safety, and cost-effectiveness of early (≤24 hours) or late (>24 hours) surgery in patients with acute traumatic SCI, for all levels of the spine. This review will ultimately serve as the basis for updating clinical practice guidelines related to the timing of surgical decompression in acute SCI. To this end, we aimed to address the following key questions:

Key Question 1: What is the effectiveness of early decompression (≤24 hours) compared with late decompression (>24 hours) or conservative therapy based on clinically important changes in neurological status? What is the effectiveness of ultra-early decompression compared with other “early” time frames up to 24 hours (eg, <8 hours vs ≥ 8 hours but <24 hours)?

Key Question 2: How does timing of decompression influence other functional outcomes or administrative outcomes?

Key Question 3: What is the safety profile of early decompression compared with late decompression?

Key Question 4: Does early decompression have differential efficacy or safety in specific subgroups of patients?

Key Question 5: What is the cost-effectiveness of early decompression compared with late decompression?

## Materials and Methods

### Electronic Literature Search

A systematic search of the literature was performed to identify studies published through September, 2021. The MEDLINE database (PubMed), Cochrane database, and EMBASE were used (Appendix A). Reference lists of newly included articles and relevant systematic reviews were also evaluated for inclusion. Studies recommended by clinical authors were also assessed for inclusion. Citations captured from the original systematic review that were identified and/or screened were removed. Results from the prior/original search were re-reviewed to ensure that relevant studies assessing time frames <24 hours were captured given the expanded scope of this review. The protocol for this updated systematic review was registered on PROSPERO (CRD42021292237).

### Study Selection and Data Abstraction

The inclusion and exclusion criteria for the systematic review are summarized in Table 1. Articles first underwent dual title and abstract screening. The full-text articles of citations that appeared to meet inclusion criteria were independently reviewed by a minimum of 2 reviewers, and disagreements were resolved by consensus.

**Table 1.** Inclusion and Exclusion Criteria: Population, Interventions, Comparators, Outcomes, Timing, and Settings.

Study Component	Inclusion	Exclusion
Population	Adults with traumatic spinal cord injury	• Pediatric patients • Pregnancy • Penetrating injury to spinal cord • Cord compression due to tumor, hematoma, degenerative disease • Patients without neurological deficit following trauma • <80% of patients had traumatic SCI
	Adults with complete or incomplete traumatic SCI at any level	
Intervention	Early decompression (≤24 hours) Ultra-early decompression (eg, <8 hours vs, ≥8 but <24 hours)	
Comparators	Delayed decompression (>24 hours) for early vs late Latest time for thresholds for ultra-early (eg, ≥8 hours but <24 hours if ultra early was defined as <8 hours for example) Conservative therapy	
Outcomes	Efficacy/effectiveness • Neurological outcomes (eg, frankel Grade, American Spinal Injury Association (ASIA) Impairment Scale (AIS), Spinal Cord Independence measure) Change in AIS grade Change in ASIA motor scores Change in sensation Functional or patient reported outcomes • Functional Independence measure, ambulatory function, others Administrative outcomes • Length of stay (eg, intensive care unit, hospital, rehabilitation) Safety outcomes • Complications, adverse events • Death • Postinjury medical complications	
Study design	• Comparative studies that control for baseline neurological status and specify timing from injury to surgery • Studies with least potential for bias (RCTs and high-quality comparative studies) • Full economic studies	• Animal studies • Nonclinical studies • Case series • n < 10 per treatment arm • Studies that do not control for baseline neurological status or severity • Studies with different definitions of early vs delayed decompression (eg, studies comparing ≤72 hours vs >72 hours)
Publication	Studies in any language with abstracts published in peer review journals	• Abstracts, editorials, letters • Duplicate publications of the same study that do not report on different outcomes • Single reports from multicenter trials • White papers • Narrative reviews • Proceedings/abstracts from meetings • Articles identified as preliminary reports when results are published in later versions

RCT = randomized control trial.

Standardized data abstraction included the following (at minimum): age, sex, completeness and level of SCI, baseline assessments, any adjunct medical therapy administered (eg, methylprednisolone), timing of decompression, and results related to neurological, functional, and safety outcomes. A second team member verified all abstracted study data for accuracy and completeness.

For all KQs, we focused on primary studies with the least potential for bias using a “best evidence” approach. Randomized control trials (RCTs) and high-quality prospective cohort studies that controlled for confounding and met inclusion criteria were included as primary evidence. For inclusion, all studies needed to control for baseline neurological status and specify the time from injury to surgery. Control for confounding was broadly interpreted to include restriction (eg, to those with American Spinal Injury Association (ASIA) Impairment Scale (AIS) A injuries only), stratification (eg, by baseline AIS), statistical methods (eg, multivariate analysis, propensity score matching), or clear demonstration that baseline neurological status was not different between groups.

### Assessment of Methodological Risk of Bias of Individual Studies

Pre-defined criteria were used to assess the risk of bias of individual studies based on the Cochrane Collaboration’s risk of bias tools for RCTs^14,15^ and Non-randomized Studies of Interventions (ROBINS-I) for observational studies.^16,17^ The risk of bias of studies included in the previous systematic review was updated using these tools. Pooled analyses and systematic reviews were assessed for bias using AMSTAR-2 criteria and/or guidance related to reporting of specialized analyses.^18-20^ Economic studies were assessed using the Quality of Health Economic Studies (QHES)^21^ while considering epidemiologic principles that may impact findings. Two team members independently appraised each included study, and discrepancies in ratings were resolved by discussion. Based on the risk of bias assessment, clinical studies were rated as “good,” “fair” or “poor” quality based on the criteria outlined in Appendix I (Table I1). Good quality studies meet most criteria for valid study methods and typically produce results that are considered valid. Fair quality studies contain several flaws (although no flaw is likely to cause major bias that would invalidate results), and/or are missing information that makes it difficult to assess limitations. This is a broad category; results from these studies may or may not be valid. Poor quality studies contain significant flaws that introduce various kinds of biases that may invalidate results.

### Data Synthesis

Data were synthesized qualitatively and quantitatively. Meta-analyses, using profile-likelihood random effects models to account for uncertainty across trials and provide more conservative estimates, were conducted to summarize data and obtain more precise effect size estimates when at least 2 studies were homogeneous enough to be combined.^22,23^ Statistical heterogeneity was assessed using Cochran’s *c*^*2*^ test and the *I*^2^ statistic.^23^ Risk ratios (RR) were calculated to evaluate associations based on dichotomous outcomes. Effect sizes for continuous variables were reported as mean differences (MD) if outcomes were based on the same or similar scale. For all effect sizes, 95% confidence intervals were reported. To maximize evaluation of available data, calculated RRs and MDs were based on raw data provided in each study. To account for differences in baseline ASIA Motor Score (AMS), the difference in change scores was used as opposed to final scores. This allowed for the incorporation of more information into the results as well as the comparison of results across studies, but did not fully adjust for baseline differences. Where adjusted estimates were reported in studies, we compared analyses using adjusted and unadjusted estimates and found no substantial difference (<10%) in our pooled estimates. When only adjusted estimates were available, the number of events for an outcome was back-calculated, assuming a baseline control risk equal to the average risk reported by the remaining studies within the same outcome. Meta-regression on SCI type (eg, complete, incomplete) and level was done where data permitted to evaluate the potential for hypothesis generation related to heterogeneity of treatment effect. Since most trials were fair quality and because removal of the estimates from the poor quality trials would not have changed the conclusions given the small sample sizes of most studies, sensitivity analyses based on study quality were not performed. Visual inspection of the forest plots and the consideration of the I^2^ of 0% indicated that no substantial statistical heterogeneity was present; thus, sensitivity analyses to explore this were not warranted. Sensitivity analysis excluding a large individual patient data (IPD) synthesis was done given the differences in study design and its substantial contribution to the pooled estimate.^4^ For calculations of pooled RRs, instances when zero events occurred within an outcome were adjusted by a fixed amount by adding .5 to all cells of a study’s contingency table so that a pooled RR could be defined. This approximation can slightly skew the study estimate in the direction of “no difference” and widens the corresponding confidence intervals. The Mantel-Haenszel method was used to determine each study’s weight in the pooled total. This method minimizes the risk of bias caused by adding a fixed amount. Prior to calculating the profile-likelihood estimates, all effect sizes were transformed to a logarithmic scale and transformed back to their original units for reporting.^24^ We ensured that patient populations included in more than 1 study were only included once for any given analysis. Calculations were carried out using Stata v13.0, and figures were created using Cochrane’s Review Manager v5.4. There were insufficient numbers of high-quality studies to effectively evaluate publication/small study bias.^25^

### Primary Outcomes

Consistent with the prior review, AMS improvement and improvement of ≥2 AIS grades were considered the primary outcomes for effectiveness. Other outcomes were reported, but overall strength of evidence was not assessed. Major complications included mortality, decubitus/pressure ulcer, surgical device-related complications requiring reoperation, sepsis/systemic infection, CSF leak, meningitis, neurological deterioration, deep wound infection, wound dehiscence, cardiopulmonary complications, tracheostomy and unplanned return to the operating room. The severity of complications was often poorly described. We considered events minor unless they were clearly reported as major or likely to be major (ie, life-threatening or requiring re-operation or invasive intervention.) An algorithm for categorizing poorly specified complications is described in Appendix H.

### Grading the Strength of Evidence for Major Comparisons and Outcomes

The overall strength (quality) of evidence (SOE) for the primary outcomes was assessed based on the application of GRADE described in the AHRQ Methods Guide.^25^ SOE was based on the totality of evidence available across studies identified in the original review and this update. One methodologist made an initial determination, which was subsequently reviewed independently by a second senior methodologist for consistency and validity before the final assessment. Disagreements were resolved by consensus. RCTs were initially considered to be high-quality evidence; however, the evidence was downgraded based on the aggregate assessment of risk of bias, consistency, imprecision, directness, and publication bias. Evaluation of reporting and publication bias is challenging in the absence of individual study protocols (reporting bias) and when few high-quality studies (eg, RCTs for publication bias) are available.^25^ Publication bias was rated as unknown for the primary outcomes in this review. Comparative observational studies were usually initially assigned as low-strength evidence. In instances where RCTs were unavailable, unethical, or not feasible, high-quality nonrandomized observational studies (NROS) provided the “best evidence.” NROS, which controlled for various biases and had few methodologic limitations, were initially considered “moderate” when such studies are at low risk of confounding.^26^ On rare occasions, observational evidence was upgraded if there was a large magnitude of effect, presence of dose-response relationship, or existence of plausible unmeasured confounders.^27^ This was considered *only* if there were no downgrades in any of the 5 primary domains, and plausible confounding would not alter conclusions. The overall SOE expresses our confidence that the observed effects for important outcomes are close to the true effects and stable, and whether new evidence is likely to change conclusions. The SOE was assigned an overall high, moderate, low, or very low grade by evaluating and weighing the combined judgments for the above domains (Appendix I; Table I2). If no studies on an outcome were identified, it was denoted as “no evidence.”

## Results

### Search Results

**Figure 1.**
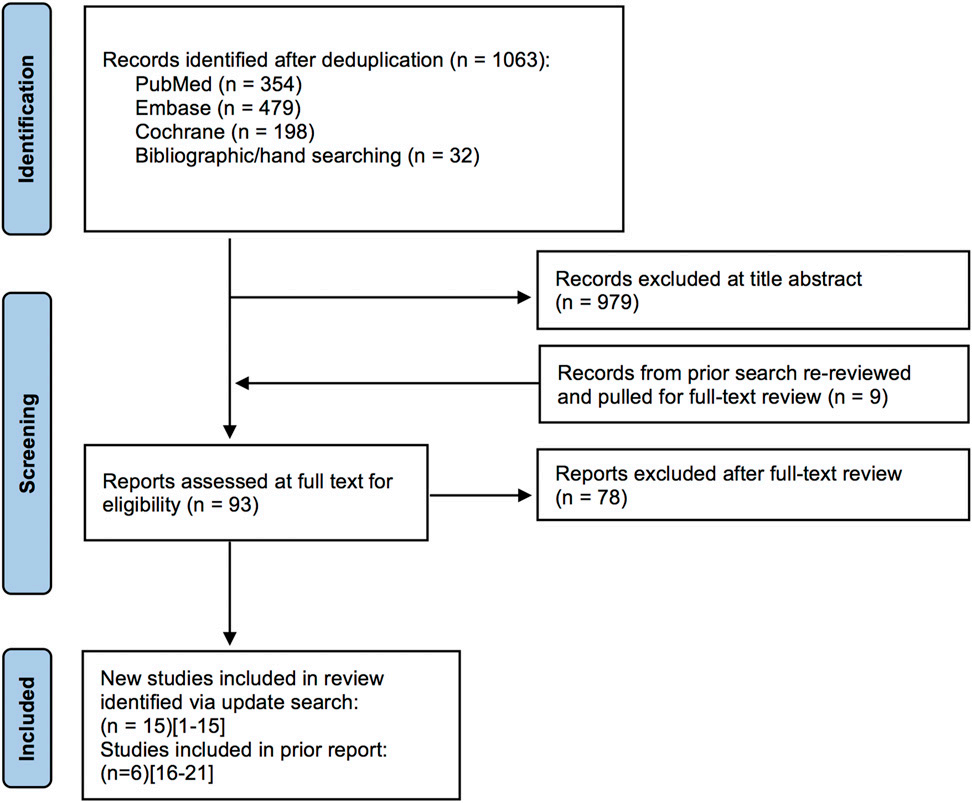
Flow chart showing results of literature search.

The search strategy yielded 1063 potentially relevant citations published since November 24, 2014. Of these, 979 were excluded at title/abstract review, 78 were excluded at full-text review, and 14 were retained. In addition, citations from the original review were evaluated for inclusion; of these, 755 were excluded at title/abstract review, 3 were excluded at full-text review, and 6 were retained^28-33^ (Figure 1). Studies suggested by clinical authors were captured in our searches, with the exception of 1 citation published ahead of print.^13^ A list of excluded studies with reasons for exclusion is provided in Appendix E. A total of 21 studies formed the evidence base for this review update.

In addition to the 6 studies included in the prior systematic review,^28-33^ which compared early surgery (≤24 hours from injury) vs late surgery (>24 hours), an additional 11 studies using this same threshold were included for this update.^4-8,11-13,34-36^ Of these, there were 6 retrospective cohort studies,^5,6,11-13,36^ 2 prospective cohort studies,^7,34^ 1 economic analysis of a previously included prospective cohort study,^35^ a pooled analysis of individual patient data (IPD) from 4 studies,^4^ and 1 RCT.^8^ The RCT was a continuation of the 2014 RCT^32^ included in the prior review and was used for updated analyses as it contained the most complete dataset.

In addition, 2 prospective^37,38^ and 2 retrospective cohort studies^5,10^ compared time frames <24 hours; one of these also provided data based on the ≤24 vs >24 hour threshold.^5^ One study comparing early surgery to nonoperative care was also identified.^9^ Patients in the STASCIS study^30^ were also represented in the IPD^4^; however, the degree of overlap could not be quantified.

### Overview of Included Studies

Details of primary study features and patient characteristics for included studies are provided in Table D1; detailed data abstraction is found in Appendix D. For the 24 hour threshold for surgical intervention, delayed time to surgery across the studies ranged from greater than 24 hours (not further defined) to 504 hours.^12^ For the <24 hour threshold, time to ultra-early surgery ranged from <4 hours to <12 hours. Inclusion/exclusion criteria varied across studies, but all patients had some level of neurological deficit at presentation, most commonly measured using the International standards for neurological classification of spinal cord injury (ISNCSCI) also known as the AIS. The initial neurological assessment was taken at the time of admission in 11 studies (in 12 publications)^5,6,8,10-12,32-34,36-38^ and within 72 hours post-injury in 3 studies;^7,29,30^ time of assessment was not reported in 2 of the surgical studies^28,31^ or in 1 study^9^ comparing timing of surgery to conservative treatment. The time of initial neurological assessment varied for the IPD.^4^

The most common causes of injury across studies that reported etiology of SCI were motor vehicle accidents or falls. Sample sizes across individual studies ranged from 35 to 888 for those investigating a 24 hour threshold, and 42 to 72 for studies exploring <24 hour thresholds. Males comprised the majority (≥65%) of the study populations, and mean patient ages ranged from 30 to 59 years. For studies on ultra-early surgery, males comprised ≥68% of the samples in each study, and mean ages ranged from 38 to 55 years. For the IPD analysis, study sample size ranged from 304 to 515; males comprised 80% of the sample, and mean patient ages ranged from 31.9 to 47.3 years. Follow-up time was <6 months in ten studies^9,12,13,29,30,33,34,36-38^ and ≥6 months in 8 studies (in 9 publications).^4-8,10,11,31,32^ One study^28^ did not specify follow-up time and so was included with the studies reporting <6 months follow-up. Follow-up rates ranged from 65.4% to 100%.

### Risk of Bias Assessment/Study Quality

The RCT (2 publications) was rated fair quality^8,32^ due to methodological limitations, including baseline differences between the intervention groups, lack of controlling for possible confounding, and lack of a prespecified threshold and definitions of key outcomes. In addition, the trial did not report on all prespecified outcomes due to problems in the hospital setting, and it was terminated early due to slow recruitment.^8^

Across the 18 observational cohort studies, one was rated good quality (the IPD analysis),^4^ 11 were rated fair quality,^5,7,12,13,28,30,33,34,36-38^ and 6 were rated poor quality.^6,9-11,29,31^ The main methodological limitations across the fair quality cohorts were related to confounding and confounding control, participant selection, and missing data and handling of missing data (ie, loss-to-follow-up); additional concerns in the poor quality cohorts included failure to clearly describe co-interventions and incomplete reporting of results.

The single economic analysis (of a previously included prospective cohort) was rated as good quality.^35^ Primary limitations included the lack of a direct incremental cost-effectiveness ratio (ICER) comparing early and late surgery and failure to explicitly discuss the direction and magnitude of potential biases. Details regarding quality ratings are summarized in Appendix B.

Key Question 1: What is the effectiveness of early decompression (≤ 24 hours) compared with late decompression (>24 hours) or conservative therapy based on clinically important changes in neurological status? What is the effectiveness of ultra-early decompression compared with other “early” time frames up to 24 hours (e.g., < 8 hours vs ≥ 8 hours but <24 hours)?

#### ASIA Motor Score Improvement

Improvement in AMS was reported in 5 studies: 1 RCT,^8^ the IPD analysis,^4^ 2 prospective cohorts^31,33^ in patients with SCI at various levels, and 1 retrospective cohort^6^ in patients with central cord syndrome. Among these, 2 studies^31,33^ were described in the prior report, and 3 studies were new^4,6,8^ (1 good quality, 3 fair quality, and 2 poor quality). These studies compared surgical decompression at ≤24 hours with >24 hours.

The 2 studies included in the prior review that discussed AMS at ≤6 months could not be pooled due to substantial clinical heterogeneity and limited data reporting. One of these was a poor quality study in patients with acute central cord syndrome and reported an additional 7.47 (95% CI -.94 to 14.91) point AMS improvement for early surgery compared with late surgery.^31^ The other fair quality study in patients with cervical, thoracic, or lumbosacral SCI reported a 13 point AMS improvement in patients receiving early surgery compared to those treated later (*P* = .01, no confidence intervals or other data provided).^33^

**Figure 2.**
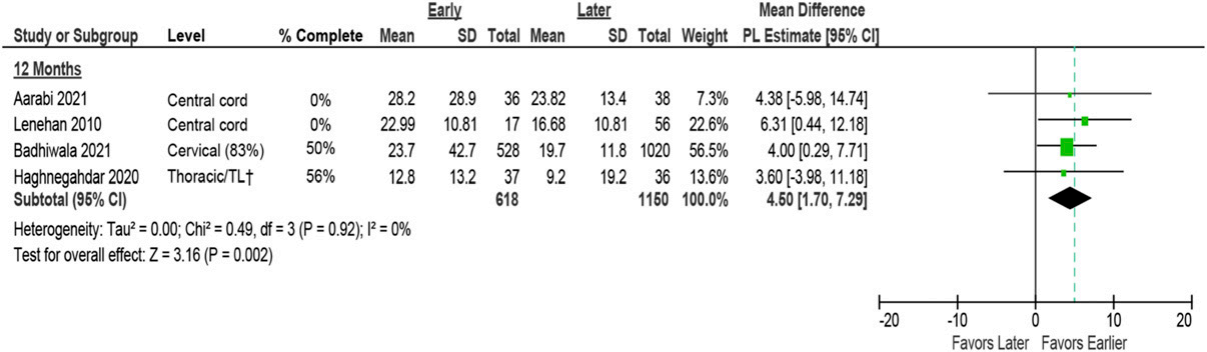
Change in ASIA Motor Score comparing early (≤24 hours) vs late (>24 hours) surgery*. AMS = ASIA Motor Score; CI = confidence interval; PL = profile-likelihood; SD = standard deviation. * The prior report considered ≥5-point change in AMS to be clinically significant; this is represented in the dashed line, † TL = thoraco-lumbar; >80% had TL injuries.

At 12 months, early surgery was associated with improved AMS across 4 studies (pooled mean difference 4.50 points, 95% CI 1.70 to 7.29, I^2^ = 0)^4,6,8,31^ (Figure 2). Sensitivity analysis excluding the IPD study resulted in a marginally higher pooled estimate but more variability (pooled MD 4.82, 95% CI 0.75 to 8.88).^4^ Results across studies, which included 2 in patients with central cord syndrome, were consistent.

None of the included studies evaluating ultra-early surgical timing thresholds reported on AMS.

#### Improvement in AIS Grade

Improvement in AIS grade was reported in 9 studies: 2 prospective cohorts^30,36^ and 2 retrospective cohorts^5,12^ in patients with cervical SCI; 1 retrospective cohort^11^ in patients with thoracolumbar SCI; 1 RCT^8^ in patients with thoracic SCI; 1 prospective cohort^7^ and 1 retrospective cohort^13^ in patients with SCI at various levels; and 1 prospective cohort^31^ in patients with central cord syndrome.

##### Early (≤24 hours) surgery versus late (>24 hours) surgery

**Figure 3.**
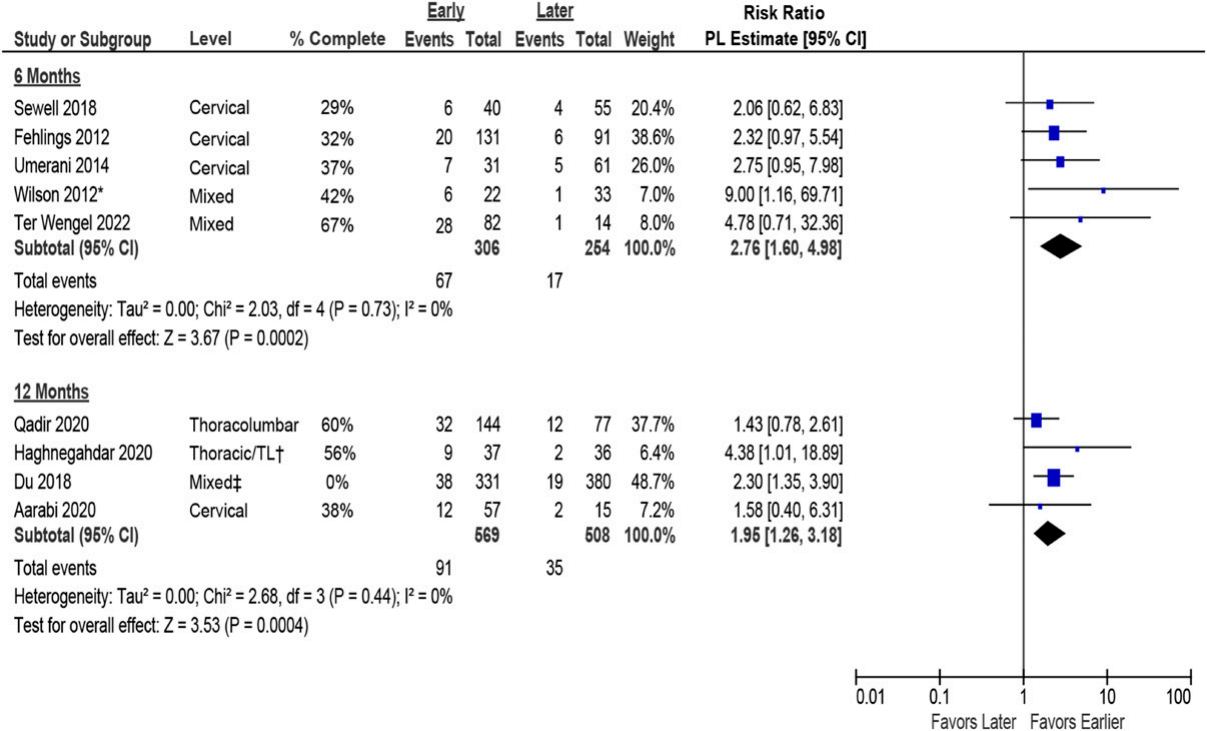
Improvement in AIS by ≥ 2 grades comparing early (≤24 hours) vs late (>24 hours) surgery. AIS = ASIA Impairment Scale; CI = confidence interval; PL = profile-likelihood. * Timing from preoperative to inpatient rehabilitation, mean 89.6 ± 47.4 days, † TL = thoraco-lumbar; >80% had TL injuries, ‡ 58% of population was thoracic, 42% had thoracolumbar.

Data comparing early (≤24 hours) vs late (>24 hours) surgery for change in AIS grade were available from 1 RCT, 7 prospective cohort studies, 5 retrospective studies, and 1 pooled analysis of IPD. A 2-fold higher likelihood of achieving a clinically important improvement in AIS grade (≥2 grades) was seen in patients receiving early vs late surgery at 6 months (5 studies, pooled RR 2.76, 95% CI 1.60 to 4.98, I^2^ = 0%).^12,13,30,33,36^ Similarly, across 4 other studies, early surgery was associated with a higher likelihood of improving by ≥ 2 AIS grades at 12 months (RR 1.95, 95% CI 1.26 to 3.18, I^2^ = 0%)^5,7,8,11^ compared with late surgery. Estimates were pooled across injury levels and completeness of SCI; however, results were consistent across studies at both time frames (Figure 3). In studies reporting AIS improvement of ≥1 grade, results similarly favored early surgery over late surgery at 6 months (7 studies, pooled RR 1.26, 95% CI 1.07 to 3.26, I^2^ = 0%)^12,13,30,31,33,34,36^ and 12 months (6 studies, pooled RR 1.17, 95%CI 0.95 to 1.43, I^2^ = 67%)^4,5,7,8,11,31^ but effect sizes were diminished (Appendix G, Figure G-1). Sensitivity analysis excluding an individual patient data (IPD) study,^4^ with a large number of patients resulted in a marginally higher pooled estimate, but more variability (pooled MD 4.82, 95% CI 0.75 to 8.88).

##### Ultra-Early Time Frame Comparisons

Data comparing various thresholds for ultra-early (≤12 hours) surgery to early (up to 24 hours) surgery for change in AIS grade were available from 4 studies: 1 prospective^38^ and 2 retrospective cohorts^5,10^ in patients with cervical SCI, and 1 prospective cohort^37^ in patients with SCI at multiple levels. Three studies^5,37,38^ were rated fair quality, and 1^10^ was rated poor quality. Surgical timing thresholds for ultra-early surgery included 4 hours,^37^ 5 hours,^10^ 8 hours,^38^ and 12 hours.^5^ Across the 4 studies^5,10,37,38^ comparing earlier times for surgery (thresholds ≤12 hours), results were inconsistent at short- and long-term endpoints, thus precluding firm conclusions about the effectiveness of ultra-early surgery on improving AIS by ≥ 2 grades. Missing data/handling of missing data was a concern across all 4 studies, as was selection bias in 2 studies.^5,10^

Table 2 summarizes the effect sizes for the ≤24 hour vs >24 hour thresholds reported above, along with those from studies that evaluated earlier surgical timing at various thresholds. Using a 24 hour threshold for surgical timing, early surgery was strongly favored as compared with late surgery. Evidence at thresholds <24 hours was inconclusive. One study^38^ suggested a large advantage to surgery within 8 hours of injury compared to >8 hours with respect to improving AIS by ≥ 2 grades at 6 months, while another^6^ study did not show an advantage to surgery within 12 hours of injury compared with >12 hours at 12 months. In contrast, 2 studies in which surgery was performed within 4^37^ or 5^10^ hours suggested substantially lower effectiveness of ultra-early surgery for improving AIS by ≥ 2 grades. At 6 months, 1 study^37^ tended to favor surgery at >4 hours from injury vs <4 hours as did the second study^10^ at 12 months using a 5 hour threshold. Across the same 4 studies, no differences were seen between ultra-early and early surgery with respect to improvement by > 1 AIS grade at either 6 or 12 months.

**Table 2.** Summary of AIS Improvement by ≥ 2 Grades at Different Surgical Timing Thresholds.

Threshold	No. of Studies (patients)	Risk Ratio (95% CI)
≤6 month follow-up		
24 hours	5 (N = 560)	2.76 (1.60 to 4.98)
8 hours	1 (N = 44)	4.55 (1.13 to 18.29)
4 hours	1 (N = 56)	.50 (CI 0.16 to 1.50)
12 month follow-up		
24 hours	4 (N = 1077)	1.95 (1.26 to 3.18)
12 hours	1 (N = 49)	1.09 (.39 to 3.04)
5 hours	1 (N = 57)	.24 (.07 to .85)

CI = confidence interval; No. = number.

##### Other Reported Neurologic Outcomes

The good quality IPD analysis across 4 studies was the only new study to include information on pin prick or light touch scores. Specifically, it reported one-stage meta-analyses across included studies adjusted for baseline score, age, mechanism of injury, AIS grade, spinal level of injury, and administration of methylprednisolone.^4^ Mean difference for change in pin prick score and light touch score at 12 months was reported as 4.0 (95% CI 1.5 to 6.6) and 4.3 (95% CI 1.6 to 7.0), respectively. One fair quality retrospective study evaluated the odds of achieving ≥10 point improvement in upper or lower extremity motor score (UEMS or LEMS) using multivariate analyses that adjusted for baseline neurological status and demonstrated a lower odds of improvement in patients receiving surgery ≥24 hours after injury (odds ratio [OR] for UEMS .02, 95% CI 0.02 to 1.23 and OR for LEMS .19, 95% CI 0.02 to 1.13).^13^

##### Early Surgery vs Conservative Treatment

1 poor quality retrospective cohort study (N = 54) included patients with pre-existing cervical spinal stenosis who experienced incomplete traumatic SCI and compared early surgery (<24 hours) with conservative care.^9^ Crude AIS improvements at 24 months favored early surgery for both ≥1 grade (OR 1.69, 95% CI 0.56 to 5.10) and ≥2 grade (OR 4.13, 95% CI 0.81 to 21.19) improvements. Multivariate linear regression of AIS grade improvement at 24 months found that improvement in the early surgery group was .543 grades higher than in the conservative group (*P* = .0044, no confidence intervals provided).

Key Question 2: How does timing of decompression influence other functional outcomes or administrative outcomes?

A total of 6 studies comparing early and late decompression (based on a 24 hour threshold) provided data on length of stay in an acute hospital setting. Two prospective studies^29,33^ in mixed populations of patients and 1 in patients with thoracolumbar SCI^32^ were included in the prior review. Three new studies were identified for this update; 2 in mixed populations^7,13^ and 1 in patients with cervical SCI.^12^ 1 new study comparing ultra-early (≤8 hours) with early surgery (>8 hours to 24 hours) in patients with cervical SCI reported length of stay.^38^

**Figure 4.**
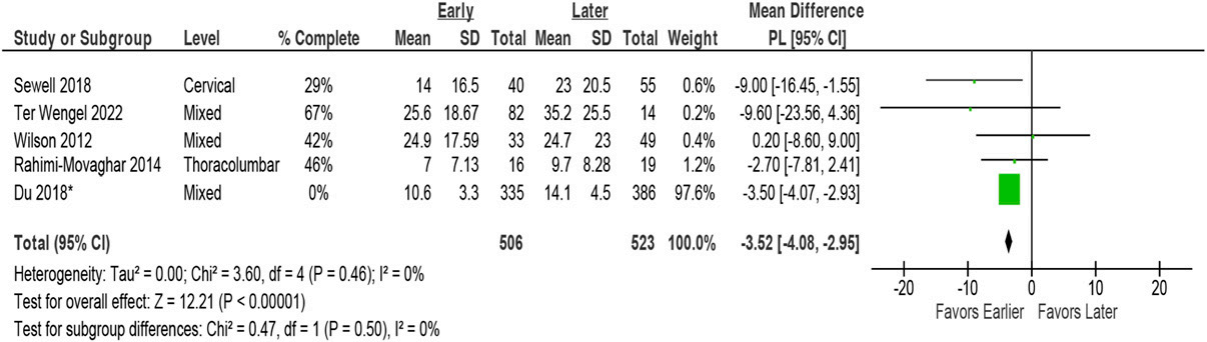
Hospital length of stay (acute care) comparing early (≤24 hours) vs late (>24 hours) surgery. CI = confidence interval; PL = profile-likelihood; SD = standard deviation. * 58% were thoracic injuries, 42% were thoracolumbar injuries.

Early surgery was associated with a small decrease in acute care hospital length of stay across 5 studies (5 studies, pooled MD –3.5 days, 95% CI, −4.1 to −3.0 days, I^2^ = 0%)^7,12,13,32,33^ (Figure 4). In addition to studies that could be pooled, 1 large poor quality registry study^29^ reported a statistically significant difference in length of stay (setting undefined) for the early compared to late surgery groups in patients with AIS A or B injury severity. However, there was limited data provided for comparison, and there was lack of clarity regarding attrition in the surgical group (Table 3).

**Table 3.** Summary of Administrative Outcomes Comparing Early (≤24 hours) vs late (>24 hours) Surgical Decompression.

Author, year Study Design	Measure	Early ≤24 hours	Late >24 hours	MD in days (95% CI)^a^
Hospital length of stay				
Mixed SCI				
Dvorak, 2015 Prospective cohort [prior report]	Length of stay Setting undefined	*AIS A patients* n = NR^b^ 7.5 days *AIS B patients* n = NR^b^ 12.8 days	*AIS A patients* n = NR^b^ Days NR *AIS B patients* n = NR^b^ Days NR	Adjusted estimates^c^ Beta: −.181 (−.303 to −.059), *P* = .004 IRR: .834 (.738 to .942) Beta: −.358 (−.590 to −.126), *P* = .003 IRR: .699 (.554 to .881)
Wilson, 2012 Prospective cohort [prior report]	Length of stay Acute care	n = 33 24.9 days	n = 49 24.7 days	.2 (8.6 to 9.0)
Ter wengel, 2022 Retrospective cohort	Length of stay Hospital	n = 82 25.59 ± 18.67 days	n = 14 35.15 ± 25.5 days	−9.6 (−23.6 to 4.4)
Du, 2018 Prospective cohort^d^	Length of stay Hospital	n = 335 10.6 ± 3.3	n = 386 14.1 ± 4.5	−3.5 (−4.1 to 3.0)
Thoracolumbar SCI				
Rahimi-movaghar, 2014 RCT [prior report]	Length of stay Setting undefined	n = 16 7.0 ± 7.13 days	n = 19 9.7 ± 8.28 days	−2.7 (−7.8 to 2.4)
Cervical SCI				
Sewell, 2018 Retrospective cohort	Length of stay Hospital	n = 40 14 (range, 2-68) days	n = 55 23 (range, 4-68) days	−9.0 (−15.5 to −1.6)
Inpatient rehabilitation length of Stay				
Mixed SCI				
Wilson, 2012 Prospective cohort [prior report]	Length of stay Rehabilitation	n = 22 102.9 days	n = 33 80.2 days	22.7 (−4.2 to 49.6)
Ter wengel, 2022 Retrospective cohort	Length of stay Rehabilitation	n = 82 168.4 ± 93.8 days	n = 14 214 ± 98.5 days	−45.6 (−73.32 to 59.39)

AIS = ASIA Impairment Score; CI = confidence interval; IRR = incidence rate ratio; MD = mean difference; NR = not reported; RCT = randomized controlled trial; SCI: spinal cord injury.

^a^Unless otherwise noted, estimates are from meta-analyses using profile likelihood methods.

^b^n at baseline was 533. Discrepancies regarding reported numbers of patients analyzed were noted. Attrition by surgical timing group was unknown for this outcome.

^c^Author reported estimates adjusted for age, sex, neurologic level, Injury Severity Score (ISS) score and injury type.

^d^58% were thoracic injuries, 42% were thoracolumbar injuries.

There was no difference between ultra-early (≤8 hours) and early (>8 to 24 hours) surgery with respect to length of hospital stay in 1 small study (MD 10.0, 95% CI –30.31 to 10.31).^38^

**Figure 5.**
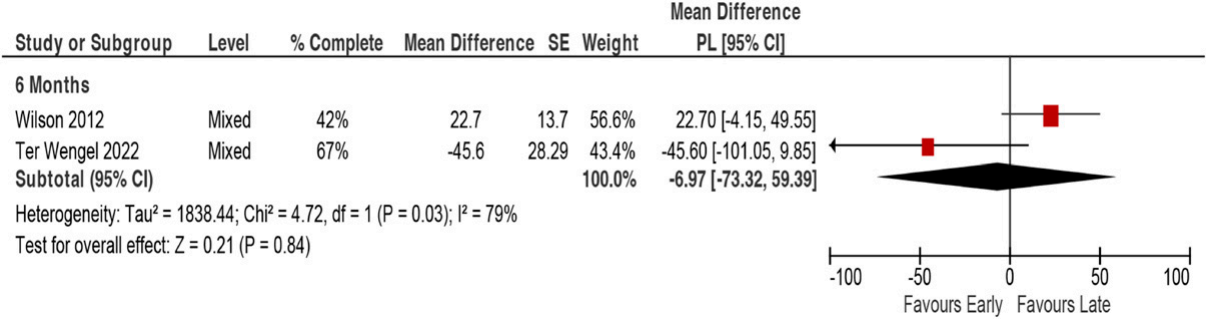
Rehabilitation length of stay comparing early (≤24 hours) vs late (>24 hours) surgery. CI = confidence interval; PL = profile-likelihood; SE = standard error.

There was no association between timing of surgery and rehabilitation length of stay across 2 studies in mixed populations (2 studies, pooled MD –6.97 days, 95% CI –73.32 to 59.4, I^2^ = 79%).^13,33^ However, there was substantial imprecision in the estimates, calling their stability into question (Figure 5, Table 3).

Since the time of the last systematic review, no new studies reporting on functional outcomes were identified. One poor quality prospective observational dataset of patients with central cord syndrome, included in the previous review, suggested that Functional Independence Measure (FIM) motor sub-score and total score improvement were greater at 12 months with early decompression (Table 4).^31^ The authors reported that propensity scoring was performed to decrease selection bias, but no details were provided. Given the wide confidence intervals, the estimates should be viewed with caution.

Key Question 3: What is the safety profile of early decompression compared with late decompression or conservative therapy?

**Table 4.** Summary of Functional Outcomes Comparing Early (≤24 hours) vs Late (>24 hours) Surgical Decompression.

Author, year Study Design	Measure	Early ≤24 hours	Late >24 hours	Group difference (95% CI)^a^
Acute Central Cord Injury without Instability				
Lenehan (2010) Prospective observational study	FIM motor sub-score improvement from discharge to 12-month follow-up	n = 17 NR	n = 56 NR	6.92 (95% CI –.11 to 13.96), *P* = .0537
	FIM total score improvement from discharge to 12-month follow-up	NR	NR	7.79 (95% CI 0.09 to 15.49), *P* = .0474

CI = confidence interval; FIM = Functional Independence Measure; NR = not reported.

^a^Authors reported that regression with propensity scoring was done to adjust for potential selection bias; however, details were not provided.

#### Early Surgery (≤24 hours) vs Late Surgery (>24 hours)

Complications were variably reported and often poorly specified in 8 studies.^5,7,8,12,13,28,30,36^ In most studies, it was not clear whether patients experienced more than 1 complication. Furthermore, the number of patients experiencing a given complication was not always described. Some studies reported “any” complication but did not specify what complications occurred or their severity (Appendix D). Complications were not reported in 7 studies.^4,6,11,28,29,31,33^

**Figure 6.**
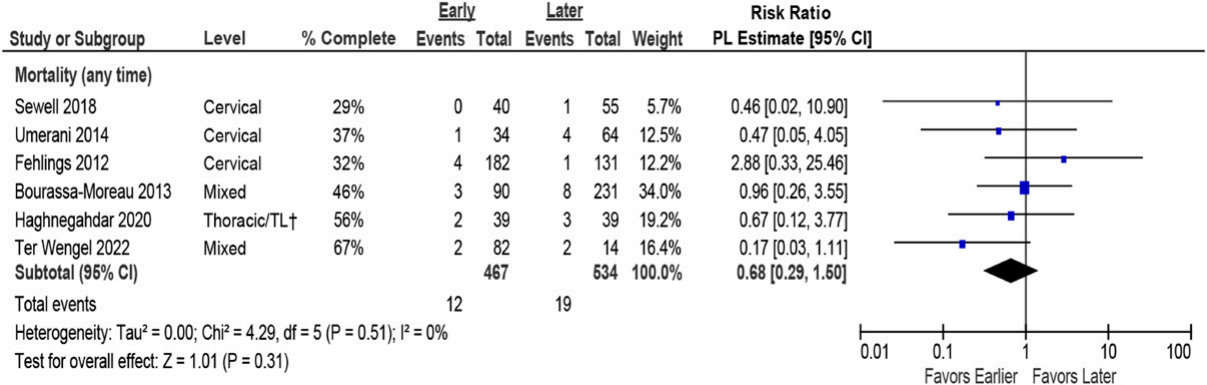
Pooled estimates for mortality comparing early (≤24 hours) vs late (>24 hours) surgery. CI = confidence interval; PL = profile-likelihood. † TL = thoraco-lumbar; >80% had TL injuries.

Pooled estimates revealed no difference in mortality based on timing of surgery (Figure 6). While mortality was rare across studies, most were likely underpowered to detect the incidence or any difference in this outcome between surgical groups.

**Figure 7.**
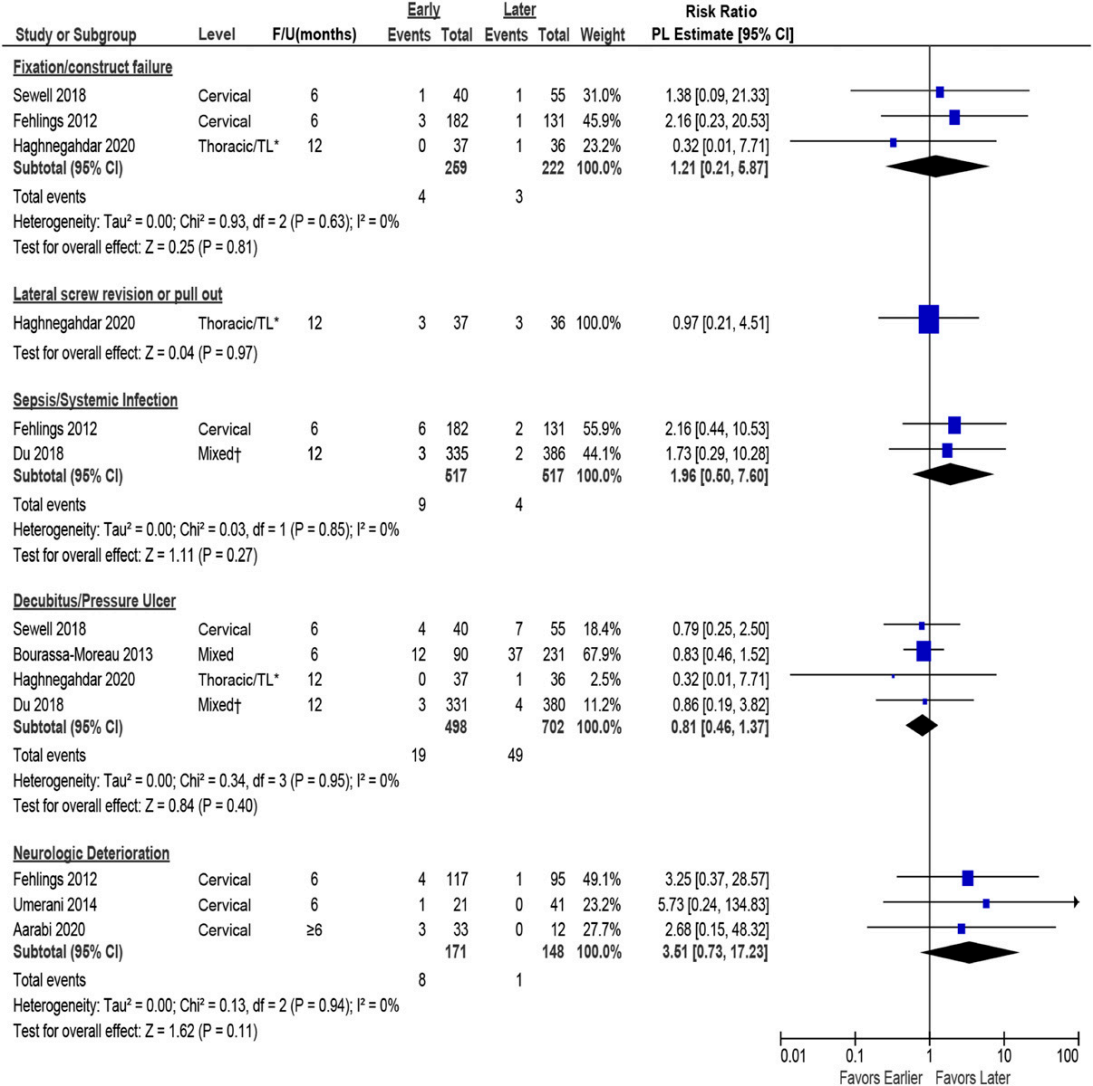
Pooled estimates for major complications (other than mortality) comparing early (≤24 hours) vs late (>24 hours) surgery. CI = confidence interval; F/U = follow-up; PL = profile-likelihood. * TL = thoraco-lumbar >80% had TL injuries, † 58% were thoracic injuries, 42% were thoracolumbar injuries.

There were no differences in the rate of major complications between early and late surgery (≤24 vs >24 hours) (Figure 7, Table 5). One fair quality study^30^ reported the total number of patients in each surgical group experiencing major complications, allowing for estimation of effect size (24% vs 30.5%; RR .79, 95% CI 0.55 to 1.14).

**Table 5.** Summary of Major Complications (other than mortality) Comparing Early (≤24 hours) vs Late (>24 hours) Surgical Decompression.

Complication	Author, year	Early, ≥24 hours, % (n/N)	Late, >24 hours, % (n/N)	RR (95% CI)^a^
Fixation/Construct Failure	3 Studies	1.5% (4/259)	1.4% (3/222)	Pooled 1.21 (.21 to 5.87)
	Sewell, 2018 (fixation failure)	2.5% (1/40)	1.8% (1/55)	1.38 (.09 to 21.33)
	Fehlings, 2012^b^ (Construct failure)	1.6% (3/182)	.8% (1/131)	2.16 (.23 to 20.53)
	Haghnegahdar, 2020 (rod failure)	0% (0/37)	2.8% (1/36)	.32 (.01 to 7.71)
Screw-related complications^c^	1 study	8.1% (3/37)	8.3% (3/36)	Pooled .97 (.21 to 4.51)
	Haghnegahdar, 2020 (revision of laterally placed screws)	8.1% (3/37)	5.6% (2/36) (3 screws)	.97 (.21 to 4.51)
	Haghnegahdar, 2020 (delayed screw pull-out)	0% (0/37)	2.8% (1/36)	.32 (.01 to 7.71)
Sepsis/systemic infection	2 studies	1.7% (9/517)	.8% (4/517)	Pooled 1.96 (.50 to 7.60)
	Fehlings, 2012^b^	3.3% (6/182)	1.5% (2/131)	2.16 (.44 to 10.53)
	Du, 2018	.9% (3/335)	.5% (2/386)	1.73 (.29 to 10.28)
Decubitus/pressure ulcer	4 studies	3.8% (19/498)	6.9% (49/702)	Pooled .81 (.46 to 1.37)
	Sewell, 2018	10.0% (4/40)	12.7% (7/55)	.79 (.25 to 2.5)
	Bourass-moreau, 2013	13.3% (12/90)	16.0% (37/231)	.83 (.46 to 1.52)
	Haghengahdar, 2020	0% (0/37)	2.8% (1/36)	.32 (.01 to 7.71)
	Du, 2018	.9% (3/331)	1.1% (4/380)	.86 (.19 to 3.82)
Neurological deterioration	3 studies	4.7% (8/171)	.7% (1/148)	Pooled 3.51 (.73 to 17.23)
	Fehlings, 2012	3.4% (4/117)	1.1% (1/95)	3.25 (.37 to 28.57)
	Umerani, 2014	4.8% (1/21)	0% (0/41)	5.73 (.24 to 134.83)
	Aarabi, 2020	9.1% (3/33)	0% (0/12)	2.68 (.15 to 48.32)
CSF leak	1 study			
	Haghnegahdar, 2020	0% (0/37)	2.8% (1/36)	Incalculable
Meningitis	1 study			
	Haghnegahdar, 2020	0% (0/37)	2.8% (1/36)	Incalculable
Wound-related events	1 study	0% (0/182)	2.3% (3/131)	Incalculable
	Fehlings, 2012^b^ (deep wound infection)	0% (0/182)	1.5% (2/131)	Incalculable
	Fehlings, 2012^c^ (wound dehiscence)	0% (0/182)	.8% (1/131)	Incalculable
Cardiopulmonary	1 study			
	Fehlings, 2012^b^	17.6% (32/182)	25.9% (34/131)	.68 (.44 to 1.04)
Unplanned return to OR	1 study			
	Du, 2018	.9% (3/335)	1.0% (4/386)	.86 (.19 to 3.83)
Tracheostomy required	1 study			
	Sewell, 2018	45.0% (18/40)	54.5% (30/55)	.82 (.54 to 1.25)

CI = confidence interval; CSF = cerebrospinal fluid; OR = operating room; RR = risk ratio.

^a^I^2^ test for heterogeneity was 0% for all pooled analyses.

^b^Denominator was total number of subjects enrolled because information on timing of complications and number of patients available was not provided.

^c^Revision of laterally placed screw or screw pull-out.

For complications where results could be pooled across studies, there were no differences between groups in the frequency of surgical device-related complications or neurological deterioration; however studies may have been underpowered to detect differences in these outcomes (Figure 7, Table 5). Across 4 studies,^7,8,12,28^ decubitus ulcers were less common with early surgery (3.8%) compared with late surgery (6.9%), although results were within the limits of chance. Similarly, in single studies, there were fewer cardiopulmonary complications (17.6% vs 25.9%)^30^ and less need for tracheostomy (45% vs 55%) in the early surgery group^12^; however, these results were also within the limits of chance.

In 1 fair quality RCT,^8^ while the risk of CSF leak and meningitis was low in patients undergoing late surgery, no patient receiving early surgery experienced this complication. Similarly, while the risk of wound-related adverse events was low with late surgery in 1 cohort study,^30^ this complication did not occur in any patient undergoing early surgery (Figure 7, Table 5). Both studies were limited by small sample sizes.

Pooled estimates across 4 studies reporting “any” complication, including minor events, suggested that many patients experience these complications following both early and late surgery (24 vs 28%) but revealed no difference between surgical groups (pooled RR .85.95% CI 0.71 to 1.03) (Appendix G, Figure G2).^7,12,13,28^

#### Ultra-Early Surgery

In 3 studies comparing other surgical timing thresholds, no differences in mortality, CSF leak, or neurological deterioration were identified between the ultra-early and late surgical groups; however, sample sizes were likely too small to detect rare events (Table 6).^5,37,38^

Key Question 4: Does early (or ultra-early) decompression have differential efficacy or safety issues in specific subpopulations of patients?

**Table 6.** Summary of Major Complications Comparing Ultra-Early vs Early Surgical Decompression.

Measure Timing	Author, year	Ultra-early <12 hours, % (n/N)	Early <24 hours, % (n/N)	RR (95% CI)
Mortality				
8 hours	Jug, 2015	7.7% (2/26)	4.5% (1/22)	1.69 (.16 to 17.44)
4 hours	Biglari, 2016	0% (0/29)	0% (0/22)	Incalculable
CSF leak				
8 hours	Jug, 2015	9.1% (2/22)	0% (0/20)	Incalculable
Neurological deteriorations				
12 hours	Aarabi, 2020	6.3% (2/32)	4.0% (1/25)	1.56 (.15 to 16.27)

CI = Confidence interval; CSF = cerebrospinal fluid; NR = not reported; RR = risk ratio.

#### Complete and Incomplete SCI

**Figure 8.**
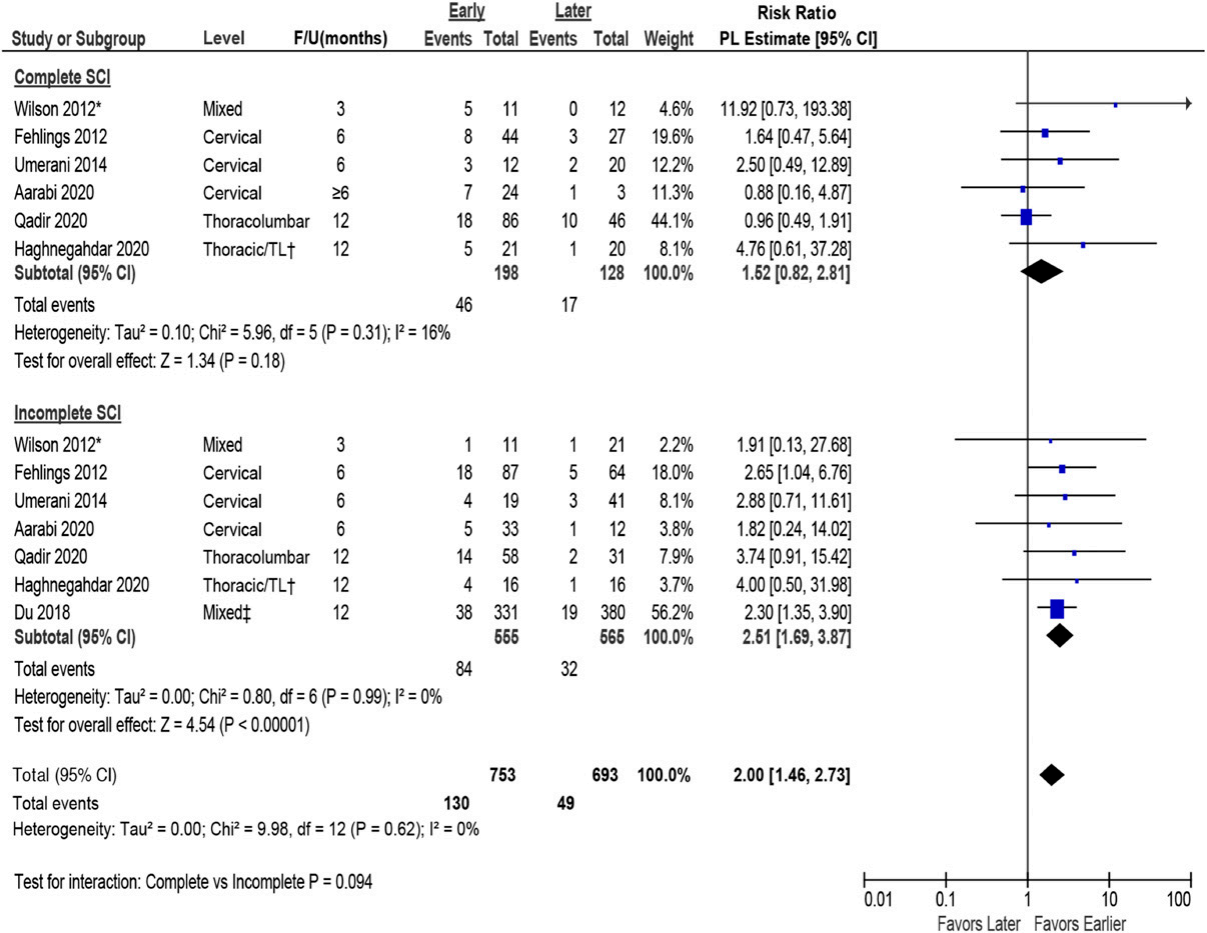
Analysis by complete and incomplete SCI on AIS improvement of ≥2 grades comparing early (≤24 hours) vs late (>24 hours) surgery. AIS = ASIA Impairment Scale; CI = confidence interval; F/U = follow-up; PL = profile-likelihood; SCI = spinal cord injury. * Timing from preoperative to inpatient rehabilitation, mean 89.6 ± 47.4 days, † TL = thoraco-lumbar; >80% had TL injuries, ‡ 58% were thoracic injuries, 42% were thoracolumbar injuries.

None of the included studies formally evaluated the differential effectiveness or safety of surgical timing in subpopulations of SCI patients. One study included in the prior report suggested that outcomes may differ in AIS A vs AIS B, C, or D patients; however, formal tests for interaction were not reported. Meta-regression across studies that separately reported data for complete and incomplete SCI suggested that completeness of injury does not modify the effect of early vs late surgery (*P*-value for interaction = .94) on AIS improvement of ≥2 grades (Figure 8) or ≥1 grade (Appendix G, Figure G3).

#### Levels

**Figure 9.**
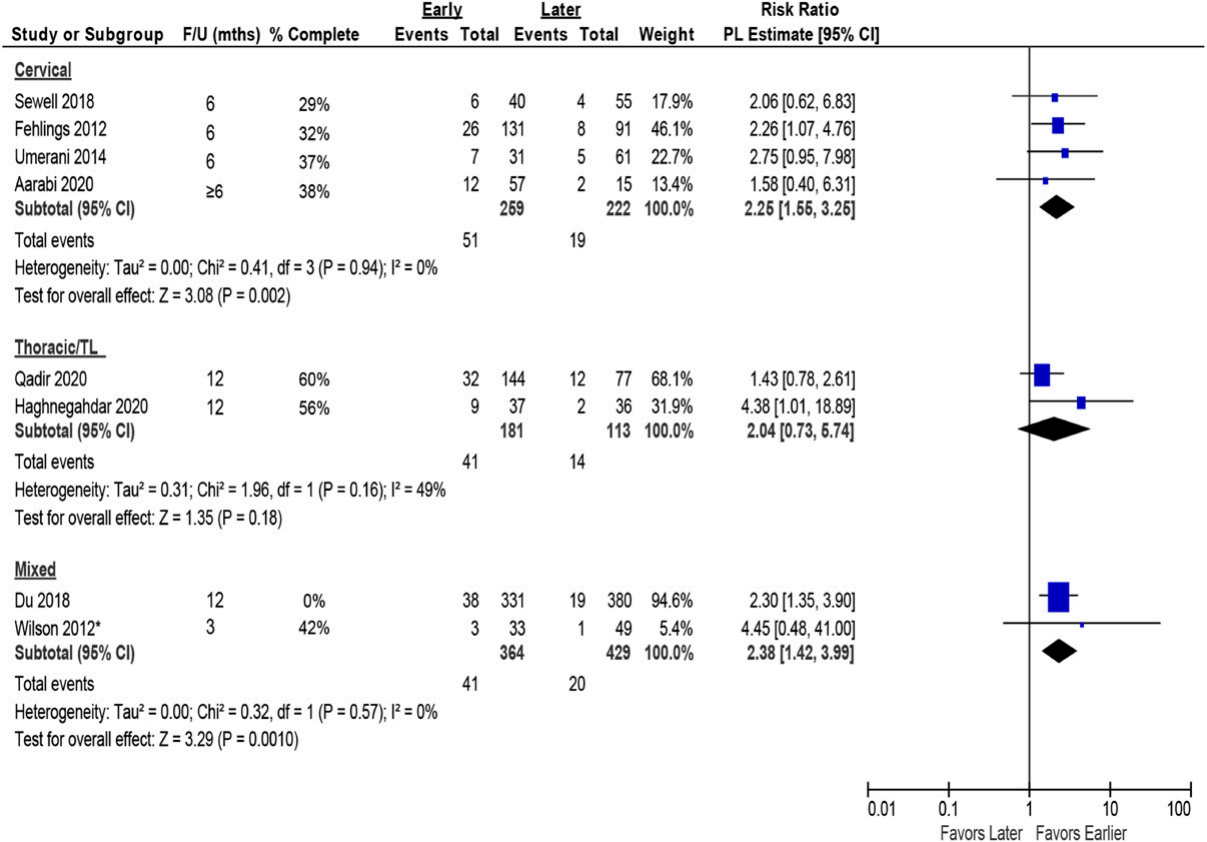
Analysis by level of SCI for AIS improvement of ≥2 grades comparing early (≤24 hours) vs late (>24 hours) surgery. AIS = ASIA Impairment Scale; CI = confidence interval; F/U = follow-up; PL = profile-likelihood; SCI = spinal cord injury. * Timing from preoperative to inpatient rehabilitation, mean 89.6 ± 47.4 days.

Data were insufficient to evaluate or draw conclusions regarding whether neurological level of injury modified the effect of early surgery. Given similarity in point estimates and overlap of confidence intervals across levels, neurological level of injury does not appear to change the treatment effect of early vs late surgery on AIS improvement by ≥ 2 grades (Figure 9) or ≥1 grade (Appendix G, Figure G4).

Key Question 5: What is the evidence of cost-effectiveness comparing the treatment options evaluated in KQ 1-4?

One good quality cost-utility study from a payer perspective evaluating early surgical decompression (≤24 hours after injury) vs late decompression (>24 hours) was identified.^35^ The decision analytic model was based on the STASCIS study^30^ of adult patients with acute traumatic cervical SCI (N = 61), which is included in this review. While the analysis suggested potential cost savings of surgical decompression within the initial 24 hours of injury in patients with either complete or incomplete SCI, the base cases and sensitivity analyses found that neither surgical timing strategy clearly dominated (Table 7).

**Table 7.** Summary of Furlan 2016 Cost-Utility Analysis of Early (≤24 hours) vs Late (>24 hours) Surgical Decompression.

	Furlan 2016 (QHES 81/100)
Population	Adults with complete or incomplete acute traumatic cervical SCI
Surgical timing comparison	≤24 hours vs >24 hours after injury
Country, currency	Canada; Costs adjusted to 2014 USD (bank of Canada conversion)
Perspective	Public payer, Ontario ministry of health and long-term Care (OMHLTC)
Study design	Cost-utility, assuming willingness to pay of $50,000 USD
Data sources	Clinical: STASCIS 2012, prospective observational study (N = 61) Costs: Direct costs (eg, hospital expenses) for acute care (toronto western hospital), rehabilitation (Ontario costing Case initiative), physicians’ fees (Ontario health insurance plan schedule)
Time horizon, discounting	6 months following injury; no discounting
Analytic model	Decision analysis: 6 possible outcomes based on surgical outcomes (postoperative complications or not), status in the first 6 months after SCI (stay in acute care or discharge from acute care), and if discharged, where the patient was discharge to (home or rehabilitation center)
Outcome (QALY, utility)	SF-6d utilities derived from SF-36
Primary findings (eg, ICER)	Potential savings of early vs late Complete SCI: $58,368,024.12 USD/QALY gained; neither strategy clearly dominant; minimal difference in QALY. Incomplete SCI: $536,217.33 USD/QALY gained; neither strategy clearly dominant; marginally higher utility (QALY) in early compared to late.
Sensitivity analysis	Threshold analysis Complete SCI and Incomplete SCI: There was no clearly dominant strategy in patients with either type of SCI; cost-effectiveness varied by which parameter was evaluated. Probabilistic (Monte Carlo simulations) Complete SCI: Early surgery was only cost-effective 26.6% of the time and was more costly/less effective than delayed surgery 23-43% of the time. The upper limit (100%) of the acceptability curve was not reached. ICERs covered at WTP thresholds ranged from 53% at $50,000 to 52.9% at $500,000 per QALY Incomplete SCI: Early surgery was cost effective 32.6% of the time and was more costly/less effective than delayed surgery 18% of the time. The upper limit (100%) of the acceptability curve was not reached. ICERs covered at WTP thresholds ranged from 59.97% at $50,000 to 58.99% at $500,000 per QALY
Author conclusions	Change toward early decompression is more likely to decrease healthcare costs. Early spinal decompression is more cost-effective compared to late decompression in patients with motor complete and incomplete SCI, even though *no strategy was dominant*. This cost-utility analysis suggests economic benefits of surgical decompression of the spinal cord within the initial 24 hours after acute traumatic cervical SCI
Funding	Cervical Spine research society Spinal Cord Injury solutions Network rapid response award from the rick hansen foundation
Primary limitations	• Possibly limited power to detect rare but potentially costly adverse outcome • Short time horizon • Limited discussion of model, study limitation and potential bias • Unclear applicability across other healthcare systems or payer structures • ICERs were provided for early and late decompression; ICER directly comparing early and late would be informative

ICER = incremental cost-effectiveness ratio; QALY = quality-adjusted life years; QHES = Quality of Health Economics Studies; SCI = spinal cord injury; USD = U.S. dollars; WTP = willingness-to-pay.

##### Evidence Summary and Strength (Quality) of Evidence

The overall SOE was assessed for the primary effectiveness outcomes of total AMS and ≥2 grades improvement in AIS (Appendix C, Tables C1 and C2) as well as for major complications (Appendix C, Table C4) where pooled estimates were possible.

Key Question 1: Early surgery (≤24 hours) may be associated with improved total AMS at short-term (≤6 months) follow-up compared with late surgery; however, confidence in this conclusion was “Very Low” given differences between the 2 available studies in populations, methods, availability of data and imprecision of effect estimates. At longer-term (>6-12 months) follow-up, there was “Moderate” evidence that early surgery improved total AMS. Similarly, early surgery conferred a 2-fold greater likelihood of a ≥2 AIS grade improvement at both short- and long-term time points (SOE “Moderate” for both).

No firm conclusions regarding the effectiveness of early surgery vs usual care in patients with incomplete SCI from 1 small poor-quality study were possible (SOE “Very low”)*.*

Firm conclusions regarding the impact of ultra-early surgery on improving AIS by ≥ 2 grades were not possible given the poor quality of studies, their imprecision, and overlap in the time frames examined (SOE “Very low”).

Key Question 2: The overall strength of evidence was “Low” that early decompression may slightly decrease acute hospital length of stay. There was no difference in rehabilitation length of stay between early and late surgery; however, there was insufficient evidence to draw firm conclusions (SOE “Very low”).

There was no difference in hospital length of stay between ultra-early (≤8 hours) and early surgery (<8 to 24 hours) groups; however, there was insufficient evidence to draw firm conclusions (SOE “Very low”).

Key Question 3: There was “Moderate” evidence from 1 study that the rate of major complications does not differ between early and late surgery. While no differences in rates of mortality, surgical device-related complications (ie, fixation or construct failure and revision of lateral screws or screw pull-out), sepsis/systemic infection (SOE “Low”), or neurological deterioration (SOE “Very low”) were seen between surgical groups, studies were likely underpowered to detect these events. There were fewer decubitus ulcers, cardiopulmonary complications, and tracheostomies in patients receiving early surgery compared with late; however, results were within the limits of chance (SOE “Low”).

Evidence on harms at earlier time frames was sparse, and conclusions regarding the impact of ultra-early surgery on adverse events was “Very low.”

Key Question 4: SOE was not assessed for KQ 4 as results were considered hypothesis-generating.

Key Question 5: SOE was not assessed for KQ 5 as there is no accepted formal system for assessing SOE across economic studies. The single included study was considered of good quality.

## Discussion

In this systematic review, we provide a comprehensive update on the current state of evidence surrounding the role of early surgical decompression on clinically relevant outcome measures after acute traumatic SCI. Since the prior 2017 systematic review and associated clinical practice guidelines, several new studies have been published investigating the timing of surgical decompression after SCI, which have increased the overall strength of evidence favoring early surgery.

Overall, the strength of evidence was moderate that early decompression (<24 hours after injury) compared to late surgery (>24 hours) leads to statistically significant improvements in neurological recovery long term. Importantly, included studies demonstrated consistent outcomes regarding neurological improvements with effects in the same direction and low heterogeneity, thus further adding to the confidence of this evidence base. Specifically, there was a mean AMS change of 4.5 (95% CI 1.70 to 7.29) in favor of early surgery in our meta-analysis, however, imprecision in the estimates was seen.

While the ISNCSCI and AMS score are the preferred tools to assess the severity and level of SCI, there remains no consensus on the minimal clinically important difference (MCID) of these outcome measures. One of the inherent challenges in establishing the MCID is that these outcome measures are not set on a linear scale, and a change in their value carries differential clinical significance based on patient and injury characteristics. A previous report using distribution-based methods suggested that the MCIDs for the upper extremity motor score (UEMS), lower extremity motor score (LEMS), and total motor score (TMS) were 2.72, 3.66, and 4.48, respectively.^39^ While MCIDs differed depending on level of injury and SCI severity, these findings would support that an AMS difference of 4.5 would likely represent a clinically significant change. Recently, MCID values for an improved measure of recovery of arm and hand function (Graded Redefined Assessment of Strength, Sensibility and Prehension) have been reported to be 8 points.^40^ Incorporation of new metrics of upper limb function in future studies may help better determine the impact of surgical timing on specific patient subgroups or injury patterns.

Similar to the previous review, we chose an AIS improvement of ≥2 grades to be clinically meaningful. A two-grade change is thought to be of high functional importance and is less likely to occur with only spontaneous recovery.^30^ In contrast, a one-grade improvement in AIS has variable clinical significance and may not as reliably correlate to a clinically meaningful improvement in neurological outcome. A pooled analysis of IPD further demonstrated a dose-response relationship between timing of decompression and neurological outcomes, specifically reporting a continuous decline in motor recovery with delayed decompression in the first 24-36 hours after injury.^4^ This provides further credence to the positive effects of early decompression.

While 1 study demonstrated greater improvements in FIM motor sub-score and total score with early decompression in patients with central cord syndrome, confidence in this measure is limited by the poor quality of the study and lack of precision (wide confidence intervals). Across studies there was a paucity of data on measures of functional independence and quality of life. Future prospective studies should therefore consider including these outcome measures.

Since the previous systematic review, several studies have emerged looking at time frames earlier than 24 hours for surgical decompression. However, the evidence pertaining to these earlier time points remains weak due to poor study quality, along with imprecision and overlap in time frames across studies. As such, while ultra-early surgery intuitively makes sense in most instances, the evidence does not allow for strong conclusions to be made for earlier time frames. Since the completion of this systematic review update, an additional prospective, multi-center observational study was published investigating neurological outcomes in patients receiving decompression surgery ≤12 hours after SCI vs >12 hours.^41^ While there was a trend in favor of ultra-early decompression (≤12 hours) for LEMS change at 12 months, this study was severely limited by imbalances in early and late surgical groups, particularly the baseline ASIA score, inadequate statistical power, and lack of reporting of clinically relevant outcome measures such as UEMS. Investigation into the role of ultra-early surgery also raises an important consideration related to the timing of baseline neurological examination. Patients undergoing ultra-early surgery are likely to have had an earlier clinical assessment, which may reduce the reliability of the exam if done in the immediate period following SCI and could be associated with a higher chance of spontaneous neurological recovery.^42^ This is an inherent limitation in assessing the clinical benefits of ultra-early surgery that must be addressed in future primary studies.

Based on the current data synthesis, SCI severity or level of injury does not appear to modify the treatment effect of early surgery. However, confidence in subgroup effects is limited due to a lack of sufficiently powered high-quality studies that explicitly evaluate subgroups based on a priori hypotheses or test for interactions. Surgical timing for the specific entity of central cord injury remains an important and controversial issue. One additional poor-quality study was identified for this update^6^; however, confidence in the findings across the 2 included studies (1 from the prior review) remains very low. Nevertheless, there is growing evidence to suggest that clinical decision making should not rely on a syndrome-based approach and that patients with the classically defined “central cord syndrome” have a similar clinical course as other patients with incomplete cervical SCI. Indeed, since this systematic review update was completed, results from a study investigating the timing of surgery in central cord syndrome patients have been published favoring early surgery.^43^ Specifically, in this analysis of 3 independent prospective multicenter datasets, early surgical decompression (<24 hours) resulted in significantly improved recovery in upper limb motor function at 12 months compared to late surgical decompression (>24 hours). This further supports the potential benefits of early surgery across different SCI phenotypes.

Compared to the previous review, there were no new updates on other functional outcomes; however, the addition of new studies permitted the pooling of data on hospital and rehabilitation length of stay. Confidence that early surgery substantially decreases hospital or rehabilitation length of stay is low and very low, respectively. The evidence pertaining to cost-effectiveness of the timing of surgery was limited to 1 full economic (cost-utility) study. While this study suggested potential cost savings of surgical decompression within 24 hours of injury, base cases and sensitivity analyses found that neither surgical timing strategy clearly dominated.

We found that current evidence is primarily low that safety and harms do not significantly differ between early and late surgical timepoints. In fact, most studies tended to favor early surgery. There is no known biological basis to suggest added harms of early compared to late surgery, provided that the appropriate expertise are available.

Our study provides the most up-to-date synthesis of the current evidence on the timing of surgical decompression for acute SCI. It is strengthened by incorporating a quantitative meta-analysis in contrast to the previous review, which was limited to a qualitative analysis. Only studies that controlled for baseline factors (ie, baseline neurologic status) were included, and we employed rigorous accepted methods for systematic reviews and registered a study protocol. Furthermore, clinically meaningful thresholds for outcome measures were chosen to examine the evidence base, and specific definitions of early decompression were used.

Despite the strengths of this systematic review, there are also several limitations that deserve note, many related to the available evidence base. There were not enough studies or publicly available data for all outcomes to formally evaluate the possibility of publication bias. Although none of the outcomes were downgraded for publication bias, the possibility of publication bias and/or selective outcome reporting cannot be ruled out. Furthermore, there was variability in injury levels (although the majority of studies were in patients with cervical SCI), and severity of SCI in patient populations reported across studies. As such, the consistency of findings is unknown for all levels; however, we found no evidence of heterogeneity of treatment effect modification by level. There was also limited and unclear reporting of some major adverse events in studies, which could have affected our synthesis of these outcomes. Furthermore, individual studies may not have had sufficient power to identify rare outcomes. Improved evaluation and reporting of harms are needed in future studies. While many studies were considered fair quality, this may partly be due to strict inclusion criteria set a priori for this review. For example, many studies were excluded that measured time from emergency department visit to surgery instead of time from injury. Nevertheless, this criterion limits the heterogeneity in study quality as well as reporting, and provides more clarity on treatment effects to facilitate clinical decision-making.

Ultimately, the current evidence base favors early surgical decompression (defined as ≤24 hours) in acute SCI compared with delayed decompression (>24 hours). As such, an update of current clinical practice guidelines is warranted.

## Supplemental Material

Supplemental Material - Timing of Decompressive Surgery in Patients With Acute Spinal Cord Injury: Systematic Review Update Supplemental Material for Timing of Decompressive Surgery in Patients With Acute Spinal Cord Injury: Systematic Review Update by Michael G. Fehlings, Laureen Hachem, Lindsay A. Tetreault, Andrea C. Skelly, Joseph R. Dettori, Erika D. Brodt, Shay Stabler-Morris, Britt J. Redick, Nathan Evaniew, Allan R. Martin, Benjamin Davies, Farzin Farahbakhsh, James D. Guest, Daniel Graves, Radha Korupolu, Stephen L. McKenna, and Brian K. Kwon in Global Spine Journal
