# Supplementary material for: Timing of Decompressive Surgery in Patients With Acute Spinal Cord Injury: Systematic Review Update
Source: Global Spine J. 2024 Mar 25;14(3 Suppl):38S–57S. doi: 10.1177/21925682231197404 (PMC10964893; doi:10.1177/21925682231197404)
Supplement: Supplemental Material - Timing of Decompressive Surgery in Patients With Acute Spinal Cord Injury: Systematic Review Update [file sj-pdf-1-gsj-10.1177_21925682231197404.pdf]

APPENDICES – Early vs. Late Decompression for Spinal Cord Injury

Contents

Appendix A. Search strategy ....

Appendix B. Risk of Bias ....

Table B1: Individual RCTs (Based on Cochrane Collaboration Tool) ....

Table B2: Individual observational studies, (based on Cochrane ROBINS-I) [1,2] ....

Table B3. Criteria for assessing studies based on AMSTAR-2. ....

Table B4. Risk of Bias Assessment for Randomized Controlled Trials ....

Table B5. Risk of Bias Assessment for Non-randomized Observational Cohort Studies ....

Table B5. Risk of Bias Assessment for Non-randomized Observational Cohort Studies, continued. ....

Table B5. Risk of Bias Assessment for Non-randomized Observational Cohort Studies, continued. ....

Table B6. Quality Assessment for Cost-Effectiveness Analysis. ....

Appendix C. Strength of evidence..

Table C1. Key Question 1: Effectiveness of early versus late decompression ....

Table C2. Key Question 1: Effectiveness of early decompression versus conservative care in patients with pre-existing spinal stenosis and AIS B or C ....

Table C3. Key Question 2: How does timing of decompression influence other functional outcomes or administrative outcomes?..

Table C4. Key Question 3: Complications ....

Appendix D. Detailed evidence tables for included studies ....

Table D1. Study characteristics and patient demographics for studies comparing early versus late decompression..

Table D2. Study characteristics and patient demographics for studies comparing other surgical timings..

Table D3. Detailed results for studies comparing early versus late decompression ....

Table D4. Detailed results for studies comparing other surgical timings ....

Appendix E. Excluded studies ....

Table E1. List of Select Excluded Studies and Rationale ....

Appendix F. Contemporary systematic reviews reporting on early vs. late timing of surgery..

Table F1. Rating overall Confidence in the Results of the Review (Dettori 2020).[7]..

Table F2. Summary table of contemporary systematic reviews reporting on early vs. late timing of surgery ....

Appendix G. Forest plots of meta-analyses for key questions ....

Figure G1. AIS improvement ≥1 AIS grade: Early surgery (≤ 24 hours) vs. late (>24 hours) [KQ1] ....

Figure G2. Any complication: Early surgery (≤ 24 hours) vs. late (>24 hours) [KQ2] ....

Figure G3. Complete/Incomplete: AIS improvement ≥1 AIS grade: Early surgery (≤ 24 hours) vs. late (>24 hours) [KQ4] ....

Figure G4. Levels: AIS improvement ≥1 AIS grade: Early surgery (≤ 24 hours) vs. late (>24 hours) [KQ4] ....

Appendix H. Algorithm for classifying adverse events ....

Appendix I.

Table I1. Criteria for grading the quality of individual studies 106

Table I2. Description of the strength of evidence grades 106

Appendix References ....

Appendix A. Search strategy

MEDLINE search

Search dates: 08/24/2014 to 09/18/2021

	**Description**	**Search terms**	**Results**	**Update (08/12/13-11/24/14)**	**Update (08/24/14-09/18/21)**
1.	SCI terms	"Spinal Cord Injuries"[MeSH] OR "Spinal Cord Compression"[MeSH] OR "Spinal Cord Ischemia"[MeSH] OR "Central Cord Syndrome"[MeSH] OR (Myelopathy AND (Trauma OR Traumas OR Traumatic OR Post-traumatic OR Posttraumatic)) OR ((Spine OR Spinal) AND (Trauma OR Traumas OR Traumatic OR Injur* OR Damag*)) OR (Cord AND (Contusion* OR Laceration* OR Transaction* OR Trauma OR Traumas OR Traumatic* OR Ischemi*)) OR "Central Cord Injury Syndrome" OR "Central Spinal Cord Syndrome" OR "Cervical Vertebrae/injuries"[MeSH] OR "Lumbar Vertebrae/injuries"[MeSH] OR "Thoracic Vertebrae/injuries"[MeSH] OR SCI OR "Paraplegia"[MeSH] OR "Quadriplegia"[MeSH] OR Paraplegi* OR Quadriplegi* OR Tetraplegi*	333,398	81,070	736,483
2.	Decompression terms	"Decompression, Surgical"[MeSH] OR Decompression OR "Spinal decompression" OR Microdecompression OR Microdiscectomy OR "Open Decompression" OR Laminectomy OR Traction OR "Mechanical Traction" OR "Inversion Therapy"	39,521	3,724	32,436
3.		#1 AND #2	7,578	627	5,560
4.	Timing terms	"Time Factors"[MeSH] OR Early[TIAB] OR Delayed[TIAB] OR Urgent[TIAB] OR Timing[TIAB]	1,164,021	140,104	896,993
5.		#3 AND #4	1,638	153	1,102
6.	Study design terms	"Comparative Study"[Publication Type] OR "Clinical Trial"[Publication Type] OR "Controlled Clinical Trials as Topic"[MeSH] OR "Randomized Controlled Trial"[Publication Type] OR "Cohort Studies"[Publication Type] OR "Prospective Studies"[Publication Type] OR RCT OR "Randomized" OR Random OR Randomly OR "Comparison" OR "Comparative" OR "Compared" OR Trial[TI] OR "Meta-Analysis"[Publication Type] OR "Multicenter Study"[Publication Type] OR "Systematic Review" OR Systematic*[TIAB]	2,380,046	388,548	2,547,638
7.		#5 AND #6	331	48	**362**

Cochrane Search:

Search dates: Database inception to 09/20/2021

ID	Search	Hits
#1	MeSH descriptor: [Spinal Cord Injuries] explode all trees	1790

#2	MeSH descriptor: [Spinal Cord Compression] explode all trees	108
#3	MeSH descriptor: [Spinal Cord Ischemia] explode all trees	10
#4	MeSH descriptor: [Central Cord Syndrome] explode all trees	5
#5	((Myelopathy AND (Trauma OR Traumas OR Traumatic OR Post-traumatic OR Posttraumatic))):ti,ab,kw (Word variations have been searched)	23
#6	(((Spine OR Spinal) AND (Trauma OR Traumas OR Traumatic OR Injur* OR Damag*))):ti,ab,kw (Word variations have been searched)	6873
#7	((Cord AND (Contusion* OR Laceration* OR Transaction* OR Trauma OR Traumas OR Traumatic* OR Ischemi*))):ti,ab,kw (Word variations have been searched)	1234
#8	(Central Cord Injury Syndrome):ti,ab,kw (Word variations have been searched)	64
#9	(Central Spinal Cord Syndrome):ti,ab,kw (Word variations have been searched)	115
#10	MeSH descriptor: [Cervical Vertebrae] explode all trees and with qualifier(s): [injuries - IN]	88
#11	MeSH descriptor: [Lumbar Vertebrae] explode all trees and with qualifier(s): [injuries - IN]	119
#12	MeSH descriptor: [Thoracic Vertebrae] explode all trees and with qualifier(s): [injuries - IN]	119
#13	(SCI):ti,ab,kw OR ("paraplegia"):ti,ab,kw OR ("quadriplegia"):ti,ab,kw OR ("paraplegic"):ti,ab,kw OR ("quadriplegic"):ti,ab,kw (Word variations have been searched)	3383
#14	("tetraplegia"):ti,ab,kw OR ("tetraplegic"):ti,ab,kw (Word variations have been searched)	333
#15	#1 OR #2 OR #3 OR #4 OR #5 OR #6 OR #7 OR #8 OR #9 OR #10 OR #11 OR #12 OR #13 OR #14	8862
#16	MeSH descriptor: [Decompression, Surgical] explode all trees	1181
#17	(Decompression):ti,ab,kw OR (Spinal decompression):ti,ab,kw OR (Microdecompression):ti,ab,kw OR (Microdiscectomy):ti,ab,kw OR (Open Decompression):ti,ab,kw (Word variations have been searched)	3767
#18	(Decompression):ti,ab,kw OR ("laminectomy"):ti,ab,kw OR ("traction"):ti,ab,kw OR ("mechanical traction"):ti,ab,kw OR ("inversion therapy"):ti,ab,kw (Word variations have been searched)	6229
#19	#16 OR #17 OR #18	6787
#20	#15 AND #19	442
#21	MeSH descriptor: [Time Factors] explode all trees	66139
#22	(early):ti,ab,kw OR (delayed):ti,ab,kw OR (urgent):ti,ab,kw OR (timing):ti,ab,kw (Word variations have been searched)	588891
#23	#21 OR #22	588891
#24	#20 AND #23	233
#25	(Comparative Study):ti,ab,kw OR (Clinical Trial):ti,ab,kw OR (Controlled Clinical Trials as Topic):ti,ab,kw OR (Randomized Controlled Trial):ti,ab,kw (Word variations have been searched)	1088811
#26	("cohort studies"):ti,ab,kw OR ("prospective studies"):ti,ab,kw OR (RCT):ti,ab,kw OR (Randomized):ti,ab,kw OR ("random"):ti,ab,kw (Word variations have been searched)	1135294
#27	("randomly"):ti,ab,kw OR ("comparison"):ti,ab,kw OR (RCT):ti,ab,kw OR ("comparative"):ti,ab,kw OR ("Compared"):ti,ab,kw (Word variations have been searched)	1280040
#28	("trial"):ti,ab,kw OR ("meta analysis"):ti,ab,kw OR (Multicenter Study):ti,ab,kw OR ("systematic review"):ti,ab,kw OR (Systematic*):ti,ab,kw (Word variations have been searched)	976165
#29	#25 OR #26 OR #27 OR #28	1417425
#30	#24 AND #29	**217**

EMBASE Search:

Search date: 09/28/21

No.	Search	Hits
#1	'spinal cord injury'/exp OR 'spinal cord injury' OR 'spinal cord compression'/exp OR 'spinal cord compression' OR 'spinal cord ischemia'/exp OR 'spinal cord ischemia' OR 'central cord syndrome'/exp OR 'central cord syndrome' OR (('myelopathy'/exp OR myelopathy) AND ('trauma'/exp OR trauma OR traumas OR traumatic OR 'post traumatic' OR posttraumatic))	329,337

	OR (('spine'/exp OR spine OR spinal) AND ('trauma'/exp OR trauma OR traumas OR traumatic OR injur* OR damag*)) OR (cord AND (contusion* OR laceration* OR transaction* OR 'trauma'/exp OR trauma OR traumas OR traumatic* OR ischemi*)) OR 'quadriplegia'/exp OR 'quadriplegia' OR 'paraplegia'/exp OR 'paraplegia'	
#2	'decompression surgery'/exp OR 'decompression'/exp OR 'spinal cord decompression' OR 'microdiscectomy'/exp OR 'laminectomy'/exp OR 'traction therapy' OR 'mechanical traction' OR 'inversion therapy'	**102,542**
#3	#1 AND #2	22,753
#4	'time factor'/exp OR early:ab,ti OR delayed:ab,ti OR urgent:ab,ti OR timing:ab,ti	2,843,167
#5	#3 AND #4	3,866
#6	#5 AND ('cohort analysis'/de OR 'comparative effectiveness'/de OR 'comparative study'/de OR 'controlled clinical trial'/de OR 'controlled study'/de OR 'evidence based medicine'/de OR 'meta analysis'/de OR 'multicenter study'/de OR 'prospective study'/de OR 'randomized controlled trial'/de OR 'systematic review'/de)	3,866
#7	#5 AND ('cohort analysis'/de OR 'comparative effectiveness'/de OR 'comparative study'/de OR 'controlled clinical trial'/de OR 'controlled study'/de OR 'evidence based medicine'/de OR 'meta analysis'/de OR 'multicenter study'/de OR 'prospective study'/de OR 'randomized controlled trial'/de OR 'systematic review'/de) AND [24-8-2014]/sd NOT [29-9-2021]/sd	591

Total hits prior to deduplication:1,170
Studies found via hand searching: 32
Total hits after deduplication: 1,063

Appendix B. Risk of Bias

Criteria Used for Determining Risk of Bias

Table B1: Individual RCTs (Based on Cochrane Collaboration Tool)

Type of Bias	Source of bias	Support for Judgment
**Selection bias**	Random sequence generation	Describe the method used to generate the allocation sequence in sufficient detail to allow an assessment of whether it should produce comparable group
	Allocation Concealment	Describe the method used to conceal the allocation sequence in sufficient detail to determine whether intervention allocations could have been foreseen before or during enrolment
**Performance bias**	Patient and personnel blinded	Describe all measures used, if any, to blind trial participants and researchers from knowledge of which intervention a participant received. Provide any information relating to whether the intended blinding was effective
**Detection bias**	Outcomes assessor blinded	Describe all measures used, if any, to blind outcome assessment from knowledge of which intervention a participant received. Provide any information relating to whether the intended blinding was effective
**Attrition bias**	Incomplete outcomes data	Describe the completeness of outcome data for each main outcome, including attrition and exclusions from the analysis. State whether attrition and exclusions were reported, the numbers in each intervention group (compared with total randomized participants), reasons for attrition or exclusions where reported, and any re-inclusions in analyses for the review
**Reporting bias**	Selective Reporting	State how selective outcome reporting was examined and what was found
**Other Bias**	Anything else, ideally prespecified	Important concerns about bias not covered in the other domains

RCT = randomized controlled trial.

Table B2: Individual observational studies (based on Cochrane ROBINS-I)[1,2]

N=no or probably no; Y=yes or probably yes; NI=not enough information to determine

Methodological Domain	
**1 Confounding**	CRITICAL
1.1 Is there potential for confounding	Y
If yes to above:	
1.2 Was the analysis based on splitting participants' F/U time according to intervention received?	N
If yes to 1.2:	
• 1.3 intervention discontinuations or switches likely related to factors prognostic for outcome?	--
1.4 Appropriate analysis method that controlled for all the important confounding domains?	N
**2 Selection of participants into the study**	MODERATE
2.1 Selection of participants based on characteristics observed after the start of intervention?	N
If yes to 2.1:	
• 2.2 Post-intervention variables that influenced selection likely associated with intervention?	--
If yes to 2.2:	
• 2.3 Post-intervention variables that influenced selection likely influenced by the outcome or a cause of the outcome?	--
2.4 Start of follow-up and start of intervention coincide for most participants?	N
If yes to 2.2 and 2.3, or no to 2.4:	
2.5 Adjustment techniques used that are likely to correct for the presence of selection biases?	Y
**3 Classification of interventions**	LOW
3.1 Intervention groups clearly defined?	Y
3.2 information used to define intervention groups recorded at the start of the intervention?	Y
3.3 Classification of intervention status affected by knowledge of outcome or of the outcome?	N
**4 Deviations from intended interventions (adhering to intervention)**	LOW
4.3. Were important co-interventions balanced across intervention groups?	Y
4.4. Was the intervention implemented successfully for most participants?	Y
4.5. Did study participants adhere to the assigned intervention regimen?	Y
If N/PN to 4.3, 4.4 or 4.5:	
• 4.6. Appropriate analysis used to estimate the effect of starting and adhering to intervention?	--
**5 Missing Data**	LOW
5.1 Outcome data available for all, or nearly all, participants?	Y
5.2 Participants excluded due to missing data on intervention status?	N

5.3 Participants excluded due to missing data on other variables needed for the analysis?	N
If PN/N to 5.1, or Y/PY to 5.2 or 5.3:	
• 5.4 Proportion of participants and reasons for missing data similar across interventions?	---
If PN/N to 5.1 or Y/PY to 5.2 or 5.3:	
• 5.5 The proportion of participants and reasons for missing data similar across interventions?	
**6 Measurement of Outcomes**	LOW
6.1 Could the outcome measure have been influenced by knowledge of the intervention received?	N
6.2 Were outcome assessors aware of the intervention received by study participants?	Y
6.3 Were the methods of outcome assessment comparable across intervention groups?	Y
6.4 Any systematic errors in outcome measurement related to intervention received?	N
**7 Reported Results**	
7.1. Estimate likely selected from multiple outcome measurements within the outcome domain?	N
7.2 Estimate likely selected from multiple analyses of the intervention-outcome relationship?	N
7.3 Estimate likely selected from different subgroups?	N
**OVERALL Risk of Bias**	**HIGH RISK**

Systematic reviews and meta-analyses (based on AMSTAR-2)[3,4]

Table B3 shows our criteria for RoB assessment based on the AMSTAR-2 tool. AMSTAR-2 is the revised and updated version of AMSTAR[5] published in 2007 used for critical appraisal of systematic reviews. It is not intended to provide an overall score, as high scores may hide weaknesses in critical domains. In light of this, we used a modified AMSTAR tool as determined by Dettori et al (2020).[6] Table B4 (adapted from Dettori 2020)[6] describes how overall scores were determined taking into account critical domains. Bold items in B3 were considered as critical items. The original AMSTAR-2 guidance suggests grading each item as either no or yes, with items 2, 4, 7, 8, and 9 allowing for a 'partial yes'. We considered a 'yes' or 'partial yes' as yes.

Table B3. Criteria for assessing studies based on AMSTAR-2

Item	Criteria
1: Did the research questions and inclusion criteria for the review include the components of PICO?	• **Yes** if all components of PICO are described somewhere in the report.<br>• **No** if any components of PICO are missing.
**2: Did the report of the review contain an explicit statement that the review methods were established prior to the conduct of the review and did the report justify any significant deviations from the protocol?**	• **Yes** if the protocol or review methods were established prior to review.<br>• **No** if no protocol or discussion of methods decided prior to review.
3: Did the review authors explain their selection of the study designs for inclusion in the review?	• **Yes** if study design inclusion is justified or discussed. No penalty for restricting study designs.<br>• **No** if no discussion of justification for inclusion.
**4: Did the review authors use a comprehensive literature search strategy?**	• **Yes** if 2 or more electronic databases were searched and key words are available in report or appendices. No penalty for language restrictions.<br>• **No** if less than 2 electronic databases were searched or key words are unavailable.
5: Did the review authors perform study selection in duplicate?	• **Yes** if selection at title/abstract and full text reviews were performed by 2 authors with consensus upon disagreement or single author selecting with a second checking agreement on sample and a kappa reported of ≥0.80.<br>• **No** if no second author involved or no kappa reported.
6: Did the review authors perform data extraction in duplicate?	• **Yes** if abstraction was performed by 2 authors with consensus upon disagreement or single author abstracting with a second checking agreement on sample and a kappa of reported of ≥0.80.<br>• **No** if no second author involved or no kappa reported.
**7: Did the review authors provide a list of excluded studies and justify the exclusions?**	• **Yes** if a list of potentially relevant studies is reported in appendix or discussed in text with citations with justification for exclusion. List of references must be provided.<br>• **No** if no list of references provided or no potentially relevant but excluded studies are discussed.
8: Did the review authors describe the included studies in	• **Yes** if study characteristics are reported in sufficient detail to determine whether the

adequate detail?	studies met PICO criteria and provides framework to judge heterogeneity.<br>• **No** if study characteristics are not reported or table 1 does not include age, sex, (and #'s).
**9: Did the review authors use a satisfying technique for assessing the RoB in individual studies that were included in the review?**	**RCTS**<br>• **Yes** if important domains similar to Cochrane.<br>**Cohort studies**<br>• **Yes** if it addresses all of the following: confounding, selection bias, measurement bias, and selective reporting of outcomes (Newcastle okay if all 8 questions included).<br>**Case series** (study of incidence, no direct comparison)<br>• **Yes** if selection bias, measurement bias, and selective reporting of outcomes met (Newcastle okay IF questions #1, 2, 3, 4, 6, 7, and 8 addressed).<br>**For all studies**<br>• **No** if there is obvious evidence that the authors misapplied an acceptable technique.
10: Did the review authors report on the sources of funding for the studies included in the review?	• **Yes** if authors report funding of individual studies.<br>• **No** if authors do not report funding.
**11: If meta-analysis was performed did the review authors use appropriate methods for statistical combination of results?**	• **Yes** if all the following are present<br>o Meta-analysis justified (e.g., studies comparable, direct comparison).<br>o Explanation of fixed or random effects (must do more than merely report without explanation).<br>o Pooled results reported separately for RCTs and cohort studies.<br>o Assessment of heterogeneity (must address I^2).<br>• **No** if one or more of the above are not present.
12: If meta-analysis was performed, did the review authors assess the potential impact of RoB in individual studies on the results of the meta-analysis or other evidence synthesis?	• **Yes** if results are stratified by RoB or if the review only included the lowest RoB studies in the analysis.<br>• **No** if results are not stratified by RoB and review includes a range of RoB outcomes in the analysis. No credit if RoB method from item #9 is not acceptable.
**13: Did the review authors account for RoB in individual studies when interpreting or discussing the results of the review?**	• **Yes** if there is a discussion of the impact of RoB in the interpretation of results and/or accounting for differences between studies.<br>• **No** if there is no discussion of the impact of RoB in the interpretation of results and/or accounting for differences between studies. No credit if method from #9 is not acceptable.
14: Did the review authors provide a satisfactory explanation for, and discussion of, any heterogeneity observed in the results of the review?	• **Yes** if I^2 demonstrates no heterogeneity (<50%) or authors explored reasons for heterogeneity if I^2 is ≥50%.<br>• **No** if I^2 demonstrates heterogeneity (>50%) and authors do not explore reasons for heterogeneity.
**15: If they performed quantitative synthesis did the review authors carry out an adequate investigation of publication bias**	• **Yes** if there is an attempt to identify publication bias. Must also show awareness of likely impact of publication bias on results. Credit given if they acknowledge publication

**(small study bias) and discuss its likely impact on the results of the review?**	bias could be a problem but not enough data given or if they have fewer than 10 studies and show no evidence of publication bias.<br>• **No** if there is no attempt to identify or discuss publication bias.
16: Did the review authors report any potential sources of conflict of interest, including any funding they received for conducting the review?	• **Yes** if authors report no competing interests or how they managed potential conflicts of interest.<br>• **No** if there is no discussion or reporting of potential conflicts of interest.

PICO = population, intervention, comparison, outcome; RoB = risk of bias.

Risk of Bias Assessments

Table B4. Risk of Bias Assessment for Randomized Controlled Trials

	Rahimi-Movghar (2014)	Haghnegahdar (2020)
**Study design**		
**Randomized controlled trial**	✓	✓
**Methodological Principle**		
Random sequence generation†	Yes	Yes
Statement of Concealed allocation†	Yes	Yes
Analysis according to random assignment†	Yes	Yes
Independent or blinded outcome assessment	Yes	Yes
Patients comparable at baseline on key characteristics	No	No
Prespecified threshold or definition of key outcomes	No	No
Attrition (≤ 20% overall)	Yes	Yes
Attrition ≤ 10% difference between groups	Yes	Yes
Comparable followup time or accounting for time at risk	Yes	Yes
Controlling for possible confounding‡	No	No
Full reporting on pre-specified outcomes	Yes	No*
**Overall Quality Rating**	**FAIR**	**FAIR**

*Due to problems in the hospital setting, we could not evaluate all outcomes of interest predefined in the protocol. Therefore, we focused on the AIS conversion rate, AMS, and complications during the hospital admission.

Table B5. Risk of Bias Assessment for Non-randomized Observational Cohort Studies

	Aarabi (2020)	Aarabi (2021)	Badhiwala (2021)*	Biglari (2016)	Bourassa-Moreau (2013)	Bourassa-Moreau (2016)	Du (2018)
**Study design**							
Prospective cohort			✓	✓		✓	✓
Retrospective cohort	✓	✓			✓		
**Methodological Principle**							
Confounding and confounding control	Moderate	Moderate	Low	Moderate	Moderate	Moderate	Moderate
Selection of participants	Serious	Serious	Moderate	Moderate	Low	Low	Moderate
Classification of interventions	Low	Low	Low	Low	Low	Low	Low
Adequate description of co-interventions†	Moderate	Serious	Moderate	Moderate	Moderate	Moderate	Moderate
Missing data and handling of missing data	Serious	Serious	Moderate	Serious	Moderate	Moderate	Moderate
Measurement of outcomes	Low	Low	Low	Low	Low	Low	Low
Reported results	Moderate	Moderate	Low	Moderate	Low	Low	Low
**OVERALL Quality Rating**	**FAIR**	**POOR**	**GOOD**	**FAIR**	**FAIR**	**FAIR**	**FAIR**

*Badhiwala 2021 - IPD, used high quality datasets and included RCT data (2 RCTs) and high-quality registry data.
† This criterion is called "deviation from intended interventions" in the ROBINS protocol.

Table B5. Risk of Bias Assessment for Non-randomized Observational Cohort Studies, continued.

	Dvorak (2015)	Fehlings (2012)	Jug (2015)	Lee (2021)	Lenehan (2010)	Mattiassich (2017)	Qadir (2020)
**Study design**							
Prospective cohort	✓	✓	✓		✓		
Retrospective cohort				✓		✓	✓
**Methodological Principle**							
Confounding and confounding control	Moderate	Moderate	Moderate	Moderate	Moderate	Serious	Serious
Selection of participants	Moderate	Low	Moderate	Serious	Serious	Serious	Serious
Classification of interventions	Low	Low	Low	Low	Low	Moderate	Low
Adequate description of co-interventions*	Serious	Moderate	Moderate	Serious	Serious	Moderate	Moderate
Missing data and handling of missing data	Critical (NI)	Serious	Moderate	Serious	Critical (NI)	Serious	Serious
Measurement of outcomes	Low	Low	Low	Low	Low	Low	Low
Reported results	Low	Low	Low	Low	Serious	Moderate	Serious
**OVERALL Quality Rating**	**POOR**	**FAIR**	**FAIR**	**POOR**	**POOR**	**POOR**	**POOR**

NI = no information.
* This criterion is called "deviation from intended interventions" in the ROBINS protocol.

Table B5. Risk of Bias Assessment for Non-randomized Observational Cohort Studies, continued.

	Sewell (2018)	ter Wengel (2022)	Umerani (2014)	Wilson (2012)
**Study design**				
Prospective cohort			✓	✓
Retrospective cohort	✓	✓		
**Methodological Principle**				
Confounding and confounding control	Moderate	Moderate	Serious	Moderate
Selection of participants	Low	Low	Moderate	Moderate
Classification of interventions	Low	Low	Low	Low
Adequate description of co-interventions*	Moderate	Serious	Serious	Moderate
Missing data and handling of missing data	Moderate	Moderate	Low	Serious
Measurement of outcomes	Low	Low	Low	Low
Reported results	Low	Low	Low	Moderate
**OVERALL Quality Rating**	**FAIR**	**FAIR**	**FAIR**	**FAIR**

* This criterion is called "deviation from intended interventions" in the ROBINS protocol.

Table B6. Quality Assessment for Cost-Effectiveness Analysis.

**Furlan (2016)**			
**Question**		**Possible Points**	**Points awarded**
1	Was the study objective presented in a clear, specific, and measurable manner?	7	7
2	Were the perspective of the analysis (societal, third-party payer, etc.) and reasons for its selection stated?	4	4
3	Were variable estimates used in the analysis from the best available source (ie, randomized controlled trial - best, expert opinion - worst)?	8	8
4	If estimates came from a subgroup analysis, were the groups prespecified at the beginning of the study?	1	1
5	Was uncertainty handled by (1) statistical analysis to address random events, (2) sensitivity analysis to cover a range of assumptions?	9	9
6	Was incremental analysis performed between alternatives for resources and costs?	6	0
7	Was the methodology for data abstraction (including the value of health states and other benefits) stated?	5	5

8	Did the analytic horizon allow time for all relevant and important outcomes? Were benefits and costs that went beyond 1 year discounted (3% to 5%) and justification given for the discount rate?	7	7
9	Was the measurement of costs appropriate and the methodology for the estimation of quantities and unit costs clearly described?	8	8
10	Were the primary outcome measure(s) for the economic evaluation clearly stated and did they include the major short-term, long-term and negative outcomes included?	6	6
11	Were the health outcomes measures/scales valid and reliable? If previously tested valid and reliable measures were not available, was justification given for the measures/scales used?	7	7
12	Were the economic model (including structure), study methods and analysis, and the components of the numerator and denominator displayed in a clear, transparent manner?	8	8
13	Were the choice of economic model, main assumptions, and limitations of the study stated and justified?	7	8
14	Did the author(s) explicitly discuss direction and magnitude of potential biases?	6	0
15	Were the conclusions/recommendations of the study justified and based on the study results?	8	0
16	Was there a statement disclosing the source of funding for the study?	3	3
**TOTAL**		**100**	**81**

Appendix C. Strength of evidence

Consistent with the prior report, the critical outcomes for determining effectiveness were improvement in AIS Grade, ASIA motor score improvement and improvement in functional outcomes (e.g., FIM).

Table C1. Key Question 1: Effectiveness of early versus late decompression

Outcome (follow-up)	Studies (N)	Risk of bias	Consistency	Directness	Precision	Reporting Bias	Early vs. Late<br>Effect size (95% CI)<br>Conclusions	Overall quality of evidence
**≤24 hours vs. >24 hours**								
**ASIA Motor Score**								
**ASIA Motor Score**<br><br>**(≥ 5-point Improvement was considered clinically important in prior review and guideline)**<br><br>***(≤6 months)***	2 studies<br>Lenehan (N=73)<br>Wilson 2012 (N= 82 acute care, N=55, IP rehabilitation	Moderate	Unknown	Direct	Imprecise	Unknown	Data were insufficient to provide meaningful pooled estimates.<br><br>**Lenehan (acute central cord syndrome): MD 95% CI**<br>7.47 (95% CI -0.94 to 14.91, p=0.0511))<br><br><u>**Wilson (cervical, thoracic or lumbosacral)**</u> IP Rehab discharge (mean 89.6 days):<br>**Early vs. Late:** adjusted parameter estimate: 13-points improvement favoring early surgery, p = 0.01 (data and confidence interval not provided)<br><br>**Conclusions:** Both studies suggest that early surgery may improve AMS short-term. However, confidence for this is very low given the differences in patient populations, study methods, lack of precision and availability of data across the studies.	Very low
**ASIA Motor Score**<br><br>**(≥ 5-point Improvement**	**4 (N= 1768)**<br>Lenehan 2010,<br>Aarabi 2021,	Low	Consistent	Direct	Imprecise	unknown	Pooled MD 4.50, 95% CI 1.70 to 7.29, I^2 = 0%)<br><br>**Conclusion:** Early surgery may confer a small improvement in total AMS compared with late	Moderate

**was considered clinically important in prior review and guideline)**<br><br>**>6-12 months**	Badhiwala 2021, Haghnegahdar 2020						surgery; it is unclear whether this would be considered clinically meaningful improvement.	
**Change in AIS ≥ 2**								
**≥ 2 AIS grade improvement** ***Short term (≤6 months)***	**5 (N= 560)** Sewell 2018, Fehlings 2012, Umerani 2014, Wilson 2012, Ter Wengel 2022	Moderate	Consistent	Direct	Precise	Unknown	Pooled RR 2.76 (95%CI 1.60 to 4.98), I^2=0%<br><br>**Conclusion:** A greater than two-fold likelihood of a ≥ 2 AIS grade improvement was observed for early vs. late surgery.	Moderate
**≥ 2 AIS grade improvement Longer term (6 - 12 months)**	**4 (N= 1077)** Qadir 2020, Haghnegahdar 2020, Du 2018, Aarabi 2020	Moderate	Consistent	Direct	Precise	Unknown	Pooled RR 1.95 (95% CI 1.26 to 3.18), I^2=0%<br><br>**Conclusion:** A two-fold greater likelihood of having a ≥ 2 AIS grade improvement was observed with early vs. late surgery.	Moderate
**Ultra-early vs. early surgery**								
**≥ 2 AIS grade improvement** ***Short term (≤6 months)***	2 studies Jug 2015 (N=44) Biglari 2016 (N=56)	High	Unknown	Direct	Imprecise	Unknown	**8-hour threshold**<br>RR 4.55 (95% CI 1.13 to 18.29) [Jug 2015]<br>**4-hour threshold**<br>RR 0.50 (95%CI 0.16 to 1.50) [Biglari 2016]<br><br>**Conclusion:** Firm conclusions on the effectiveness of ultra-early surgery (< 4 hours or < 8 hours) to improve AIS by ≥ 2 AIS grades by 6 months are not possible	Very low

**≥ 2 AIS grade improvement Longer term *(12 months)***	2 studies Mattiassich 2017 (N= 49) Aarabi 2020 (N= 57)	High	Unknown	Direct	Imprecise	Unknown	**5-hour threshold**<br>RR 0.24 (95% CI 0.07 to 0.85) [Mattiassich 2017]<br><br>**12-hour threshold**<br>RR 1.09 (95% CI 0.39 to 3.04) [Aarabi 2020]<br><br>**Conclusion:** Firm conclusions on the effectiveness of ultra-early surgery (< 5 hours or < 12 hours) to improve AIS by ≥ 2 AIS grades by 12 months are not possible.	Very low

AIS = Asia Impairment Scale; ASIA = American Spinal Cord Injury Association Impairment Scale; CI = confidence interval; IP = inpatient; MD = mean difference; RR = risk ratio.

Table C2. Key Question 1: Effectiveness of early decompression versus conservative care in patients with pre-existing spinal stenosis and AIS B or C

Outcome (follow-up)	Studies (N)	Risk of bias	Consistency	Directness	Precision	Reporting Bias	Early vs. Late<br>Effect size (95% CI)<br>Conclusions	Overall quality of evidence
**≥ 2 AIS grade improvement**<br>**Longer term *(24 months)***	1 (N= 54)<br>Lee 2021	High	Unknown	Direct	Imprecise	Unknown	Crude OR 4.13 (95% CI 0.81 to 21.19)<br><br>**Conclusion:** Firm conclusions on the effectiveness of early surgery compared with conservative treatment in patients with pre-existing cervical spinal stenosis who experienced incomplete traumatic AIS are not possible.	Very low

AIS = Asia Impairment Scale; CI = confidence interval; OR = odds ratio

Table C3. Key Question 2: How does timing of decompression influence other functional outcomes or administrative outcomes?

Outcome (follow-up)	Studies (N)	Risk of bias	Consistency	Directness	Precision	Reporting Bias	Early vs. Late<br>Effect size (95% CI)<br>Conclusions	Overall quality of evidence
**How does timing of decompression influence functional outcomes?**								
**Acute central cord injury without instability**								
**FIM Motor Sub-Score and Total Score Improvement (from discharge to follow-up)**<br>***Longer term (12 months)***	1 prospective observational study [Lenehan, 2010]<br>N = 73	High	Unknown	Direct	Imprecise	Unknown	**FIM motor sub-score**<br>MD 6.92 (95% CI -0.11 to 13.96), p=0.0537<br><br>**FIM total score**<br>MD 7.79 (95% CI 0.09 to 15.49), p=0.0474<br><br>**Conclusion:** Although early surgery tended to improve FIM motor scores compared with late surgery, the estimates should be viewed cautiously given quality and lack of precision.	Very Low
**How does timing of decompression influence administrative outcomes? – Length of Stay**								
**Early (≤24 hours) and late (>24 hours)**								

**Hospital LOS for acute care**	5 (N= 1029)* Sewel 2018 Ter Wengel 2022 Wilson 2012 Rahimi-Movaghar 2014 Du 2018	Moderate	Consistent	Direct	Precise	Unknown	Pooled MD -3.52 days, 95%CI -4.1 to 3.0, I^2= 0% **Conclusion:** Pooled estimates across 5 studies suggest early surgery (≤24 hours) is associated with an average of 3 days shorter hospital LOS compared with late surgery (>24 hours). The impact of this difference on costs or other outcomes is not known	Low
**LOS for inpatient rehabilitation**	2 (N= 151) Wilson 2012 Ter Wengel 2022	Moderate	Inconsistent	Direct	Imprecise	Unknown	Pooled MD -6.97 days (95% CI -73.32 to 59.4, I^2= 79%**)** **Conclusion:** While there was no difference in rehabilitations length of stay, between early and late surgery groups, there is insufficient evidence across 2 studies to draw firm conclusions regarding the impact of surgical timing on rehabilitation LOS.	Very Low
**Ultra-early (≤8 hours) and early (>8 to 24 hours)**								
**Hospital LOS for acute care**	1 (N= 44) Jug 2015	Moderate	Unknown	Direct	Imprecise	Unknown	MD 10.0 [-30.31, 10.31] **Conclusion:** While there was no difference in LOS between ultra-early and early surgery, there is insufficient evidence to draw firm conclusions.	Very Low

AIS: ASIA Impairment Score; FIM = Functional Independence Measure; IRR: Incident Rate Ratio; LOS: length of stay; MD = mean difference; NR: Not Reported; RCT = randomized controlled trial; SCI: spinal cord injury

* An additional poor-quality study included in the prior report (Dvorak 2015); Lack of data on mean LOS for the late group and lack of clarity on appropriate denominator for this outcome precluded pooling with other studies and results could not be verified and are not considered in this strength of evidence rating. Results available in the full report.

Table C4. Key Question 3: Complications

Outcome (follow-up)	Studies (N)	Risk of bias	Consistency	Directness	Precision	Reporting Bias	Early vs. Late Effect size (95%CI) Conclusions	Overall quality of evidence
**≤24 hours vs. >24 hours**								
**Any Major Complications**	1 (N= 313)<br>Fehlings 2012†	Moderate	Unknown	Direct	Precise	Unknown	24.2 (44/182) vs. 30.5% (40/131)<br>RR 0.79 (95% CI 0.55 to 1.14)<br><br>**Conclusion:** There were no differences in the rate of major complications between early and late surgeries.	Moderate
**Mortality**	6 (N=1001)<br>Sewell 2018,<br>Umerani 2014,<br>Fehlings 2012†,<br>Bourassa-Moreau 2013,<br>Haghnegahdar 2020,<br>Ter Wengel 2022	Moderate	Consistent	Direct	Imprecise*	Unknown	2.6% (12/467) vs. 3.6% (19/534)<br>Pooled RR 0.68 (95% CI 0.29 to 1.50), I^2= 0%<br><br>**Conclusion:** There were no differences in mortality between early and late surgery, however most studies were likely underpowered to detect this.	Low
**Surgical device related**	**Fixation or construct failure**<br>3 (N=481)<br>Sewell 2018,<br>Fehlings 2012†,<br>Haghnegahdar 2020<br><br>**Revision of lateral screws or screw pull-out**<br>1 (N=73)<br>Haghnegahdar 2020	Moderate	Consistent	Direct	Imprecise*	Unknown	**Fixation or construct failure**<br>1.5% (4/259) vs. 1.4% (3/222)<br>Pooled RR 1.21 (95% CI 0.21 to 5.87), I^2= 0%<br><br>**Revision of lateral screws or screw pull-out**<br>8.1% (3/37) vs. 8.3% (3/36)<br>Pooled RR 0.97 (95% CI 0.21 to 4.51), I^2= 0%<br><br>**Conclusion:** There were no differences between early and late surgical intervention with regard to	Low

							surgical-device related complications, however to the extent these are rare, studies may have been underpowered to detect them or differences between surgical groups.	
**Sepsis, systemic infection**	2 (N=1,034)<br>Fehlings, 2012†<br>Du 2018	Moderate	Consistent	Direct	Imprecise*	Unknown	1.7% (9/517) vs. 0.8% (4/517)<br>Pooled RR 1.96 (95% CI 0.50 to 7.60) I^2= 0%<br><br>**Conclusion:** There were no differences between early and late surgical intervention in the frequency of sepsis; studies were likely underpowered to detect this.	Low
**Decubitus, Pressure ulcer**	4 (N=1200)<br>Sewell 2018,<br>Bourassa-Moreau 2013,<br>Haghnegahdar 2020	Moderate	Consistent	Direct	Precise	Unknown	3.8% (19/498) vs. 6.9% (49/702)<br>Pooled RR 0.81 (95% CI 0.46 to 1.37), I^2= 0%<br><br>**Conclusion:** There were fewer decubitus ulcers in patients receiving early surgery compared with late, but results were within the limits of chance.	Low
**Neurological deterioration**	3 (N=319)<br>Fehlings 2012†<br>Umerani 2014<br>Aarabi 2020	Moderate	Consistent	Direct	Imprecise*	Unknown	4.7% (8/171) vs. 0.7% (1/148)<br>Pooled RR 3.51 (95% CI 0.73 to 17.23), I^2= 0%<br><br>**Conclusion:** There were no differences between early and late surgical intervention in the frequency of neurological deterioration; studies were likely underpowered to detect this.	Very Low

**Cardiopulmonary**	1 (N=313)<br>Fehlings 2012†	Moderate	Unknown	Direct	Precise	Unknown	17.6% (32/182) vs. 25.9% (34/131)<br>RR 0.68 (95% CI 0.44 to 1.04)<br><br>**Conclusion:** There were fewer cardiopulmonary complications in the early surgery group compared with later surgery, however, but results were within the limits of chance.	Low
**Tracheostomy required**	1 (n=95)<br>Sewell 2018	Moderate	Unknown	Direct	Precise	Unknown	45% (18/40) vs. 55% (30/55)<br>RR 0.82 (95% CI 0.54 to 1.25)<br><br>**Conclusion:** There were fewer tracheostomies required in patients receiving early surgery compared with late surgery, but results were within the limits of chance.	Low
**Ultra-early vs. early**								
**Mortality**	2 (N=100)<br>Jug 2015<br>Biglari 2016	Moderate	Unknown	Direct	Imprecise*	Unknown	**8-hour** threshold: 7.7% (2/26) vs. 4.5% (1/22), RR 1.69 (95% CI 0.16 to 17.44) [Jug 2015]<br><br>**4-hour** threshold: 0% (0/29) vs. 0% (0/22) [Biglari 2016]<br><br>**Conclusion:**<br>There were no differences in mortality for either threshold, however studies were likely underpowered to detect this.	Very Low
**CSF leak**	1 (N=44)<br>Jug, 2015	Moderate	Unknown	Direct	Imprecise*	Unknown	**8-hour** threshold: 9.1% (2/22) vs. 0% (0/20), RR 4.36 (95% CI 0.22 to 84.28)	Very Low

							**Conclusion:**<br>There was no difference between early and late surgery in the rate of CSF leak however the study was likely underpowered to detect a difference.	
**Neurologic deterioration**	1 (N=57)<br>Aarabi, 2020	Moderate	Unknown	Direct	Imprecise*	Unknown	**12-hour** threshold: 6.3% (2/32) vs. 4.0% (1/25), RR 1.56 (95% CI 0.15 to 16.27)<br><br>**Conclusion:**<br>There was no difference between early and late surgery in the frequency of neurological deterioration; the study was likely underpowered to detect a difference.	Very Low

CI = confidence interval; CSF = cerebrospinal fluid; RR = risk ratio.
* This appears to be a rare event and studies may have been underpowered.
†Denominator is total number of subjects enrolled because information on timing of complications and number of patients available is not provided.

Appendix D. Detailed evidence tables for included studies

Table D1. Study characteristics and patient demographics for studies comparing early versus late decompression

Author (year)<br>Injury<br>Injury level	Study design (Quality)	Demographics	Follow-up (% followed)	Baseline neurological, disease, and trauma severity status	Patient characteristics	Intervention(s) and Co-intervention(s)	Timing of treatment	Inclusion/Exclusion<br><br>Adjustment for baseline neuro-status
**Central cord SCI**								
Lenehan 2010<br><br>Acute central cord injuries without instability<br><br>Complete/incomplete<br><br>From prior report	Prospective cohort (Poor)	**Early (≤24 h)**<br>N = 17<br>Mean age ± SD: 55.0 ± 14.4 years<br>Male: 82.3%<br><br>**Late (>24 hours):**<br>N = 56<br>Mean age ± SD: 59.1 ± 14.3 years<br>Male: 80.3%	6 months (% NR)<br><br>12 months (% NR)	Timing of initial neurological examination NR.<br><br>**Co-morbidity, % (n/N):**<br>Early:<br>Yes: 17.7% (3/17)<br>No: 82.4% (14/17)<br>Late:<br>Yes: 23.2% (13/56)<br>No: 76.8% (43/56)<br><br>**Baseline AIS grade, % (n/N):**<br>Early:<br>C: 52.9% (9/17)<br>D: 47.1% (8/17)<br>Late:<br>C: 33.9% (19/56)<br>D: 66.1% (37/56)<br><br>**Baseline AMS, mean ± SD:**<br>Early: 61.1 ± 29.2<br>Late: 63.5 ± 25.1	**Mechanism of Injury, % (n/N):**<br>Early:<br>Falls: 52.9% (9/17)<br>MVA: 23.5% (4/17)<br>Other: 23.53% (4/17)<br>Late:<br>Falls: 66.1% (37/56)<br>MVA: 17.9% (10/56)<br>Other: 16.1% (9/56)<br><br>**Surgical approach, % (n/N):**<br>Early:<br>Anterior: 18.8% (3/17)<br>Posterior: 37.5% (6/17)<br>Combined: 43.8% (7/17)<br>Late:	**Interventions**<br>• Surgical decompression.<br><br>**Co-interventions**<br>NR	From timing of injury to surgery.<br><br>**Time from injury to surgery, mean ± SD**<br>Total: 67.7 ± 85.7 hours	**Inclusion:**<br>• Presenting with acute central cord syndrome, AIS C or D, sacral sensory sparing, motor score greater in lower limbs than in the upper limbs.<br><br>**Exclusion:**<br>• Instability secondary to a fracture/fracture dislocation, acute traumatic cervical disc herniation.<br><br>**Adjusted for:**<br>• Regression analysis uses propensity score stratification.

					Anterior: 27.8% (15/56)<br>Posterior: 59.3% (32/56)<br>Combined: 12.9% (7/56)			
Aarabi (2021)<br><br>Acute traumatic central cord syndrome<br><br>Incomplete	Retrospective cohort (Poor)	**Early (≤24 h)**<br>N = 36<br>Mean age ± SD: 58.1 ± 11.3 years<br>Male: 86.1%<br><br>**Late (25-72 h)**<br>N = 38<br>Mean age ± SD: 57.4 ± 12.4 years<br>Male: 76.3%<br><br>**Very late (>72 h)**<br>N = 27<br>Mean age ± SD: 58.2 ± 11.8<br>Male: 81.5%	≥6 months: 100% (101/101)<br><br>Early: 100% (36/36)<br><br>Late: 100% (38/38)<br><br>Very late: 100% (27/27)	Timing of initial neurological examination performed upon arrival by trauma surgeons and then again by a neurosurgical team once determined to be medically stable.<br><br>**Baseline AIS grade, % (n/N):**<br>Early:<br>C: 27.8% (10/36)<br>D: 72.2% (26/36)<br>Late:<br>C: 28.9% (11/38)<br>D: 71.1% (27/38)<br>Very late:<br>C: 11.1% (3/27)<br>D: 88.9% (24/27)<br><br>**Baseline AMS, mean ± SD:**<br>Early: 62.9 ± 24.3<br>Late: 68.08 ± 24.7<br>Very late: 75.9 ± 18.6	**Surgical technique, % (n/N):**<br>Early:<br>ACDF/ACCF: 36.1% (13/36)<br>ACDF + laminectomy: 25.0% (9/36)<br>Laminectomy or expansive laminoplasty: 38.9% (14/36)<br>Late:<br>ACDF/ACCF: 42.1% (16/38)<br>ACDF + laminectomy: 15.8% (6/38)<br>Laminectomy or expansive laminoplasty: 42.1% (16/38)<br>Very late:<br>ACDF/ACCF: 40.7% (11/27)<br>ACDF + laminectomy: 3.7% (1/27)<br>Laminectomy or expansive laminoplasty:	**Interventions**<br>• ACDF/ACCF<br>• ACFF + laminectomy<br>• Laminectomy or expansive laminoplasty<br><br>**Co-interventions**<br>• Methylprednisolone if needed between the years 2000-2009, but discontinued in new participants starting in 2010.	From timing of injury to decompression.<br>**Time from injury to surgery, mean ± SD**<br>NR	**Inclusion:**<br>• Patients with blunt traumatic cervical SCI, presented as acute traumatic central cord syndrome, AMS ≤95, AIS grades C and D, no mechanical instability on CT scan, presence of high intensity signal change on T2 weighted image or short T1 inversion recovery imagines indicating evidence of traumatic cervical SCI, presence of spinal stenosis or disc osteophyte complex, no evidence of disco ligamentous injury on MRI except for minor extension distraction, underwent surgery for spinal cord decompression, post-operative MRI indicated presence of CSF interface between spinal cord and dura indicating complete compression, and followed for at least 6 months after acute

					55.6% (15/27)<br><br>**Received methylprednisolone, % (n/N):**<br>Early: 30.6% (11/36)<br>Late: 31.6% (12/38)<br>Very late: 51.9% (14/27)			care discharge in order to determine long-term AMS.<br><br>**Exclusion:**<br>• AMS 96-100, inadequate follow-up, inadequate decompression on postoperative MRI, no postoperative MRI, death during their acute care in the hospital, or no medical records.<br><br>**Adjusted for:**<br>• Time from injury to surgery, age, gender, etiology, baseline AMS, baseline AIS grade, number of stenosed skeletal segments, maximum canal compromise, point of maximum compression, number of high intensity signals on MRI, and intramedullary lesion length.
**Cervical SCI**								
Sewell (2018)<br><br>Cervical SCI<br><br>Complete/incomplete	Retrospective cohort (Fair)	**Early (≤24 h)**<br>N = 40<br>Median age (range): 42 (16 to 78) years<br>Male: 67.5%<br><br>**Late (>24 h)**	6 months: 98.9% (94/95)<br><br>Early: 100% (40/40)<br><br>Late: 98.2%	Initial neurologic exam performed preoperatively.<br><br>**Baseline AIS grade, % (n/N)**<br>Early:<br>A: 27.5% (11/40)	**Mechanism of injury, % (n/N)**<br>Early:<br>Road traffic accident: 60% (24/40)<br>Fall: 35% (14/40)<br>Assault: 5% (2/40)	**Interventions**<br>• Surgical decompression.<br><br>**Co-interventions**<br>NR	From time of injury to surgery.<br><br>**Time from injury to surgery, median**	**Inclusion:**<br>• Patients ≥16 years with traumatic cervical SCI, GCS score >13, and concomitant chest injury necessitating ICU admission.

		N = 55<br>Median age (range): 46 (18 to 83) years<br>Male: 65.4%	(54/55)	B: 42.5% (17/40)<br>C: 17.5% (7/40)<br>D: 12.5% (5/40)<br>E: 0% (0/40)<br>Late:<br>A: 41% (17/55)<br>B: 35% (19/55)<br>C: 18% (10/55)<br>D: 16% (9/55)<br>E: 0% (0/55)<br><br>**Cervical cord injury, % (n/N)**<br>Early:<br>C1-C3: 7.5% (3/40)<br>C3-C6: 70% (28/40)<br>C6-T1: 22.5% (9/40)<br>Late:<br>C1-C3: 14.5% (8/55)<br>C3-C6: 62% (34/55)<br>C6-T1: 23.5% (13/55)<br><br>**Chest injury, % (n/N)**<br>Early:<br>Pulmonary contusions: 75% (30/40)<br>Hemopneumothorax: 20% (8/40)<br>Pneumothorax: 5% (2/40)<br>Late:<br>Pulmonary contusions: 64% (35/55)<br>Hemopneumothorax: 31% (17/55)<br>Pneumothorax: 5%	Late:<br>Road traffic accident: 56% (31/55)<br>Fall: 40% (22/55)<br>Assault: 4% (2/55)		**(range)**<br>Early: 18 (8 to 24) hours<br>Late: 73 (25 to 504) hours	**Exclusion:**<br>• Patients with head injuries (GCS score ≤13).<br><br>**Adjusted for:**<br>• Age.<br>• Complete/incomplete SCI.<br>• Performance of tracheostomy.

				(3/55)<br><br>**American Society of Anesthesiologists grade, % (n/N)**<br>Early:<br>1: 72.5% (29/40)<br>2: 22.5% (9/40)<br>3: 5% (2/40)<br>Late:<br>1: 65.5% (36/55)<br>2: 29% (16/55)<br>3: 5.5% (3/55)				
Fehlings (2012)*<br><br>Cervical SCI<br><br>Complete/incomplete<br><br>STASCIS trail (multi-center)<br><br>From prior report	Prospective cohort (Fair)	**Early (<24 h)**<br>N = 182<br>Mean age ± SD: 45.0 ± 17.2 years<br>Male: 76.9%<br><br>**Late (≥24 h)**<br>N = 131<br>Mean age ± SD: 50.7 ± 15.9 years<br>Male: 73.3%	6 months 70.9% (222/313)<br><br>Early: 72.0% (131/182)<br><br>Late: 69.5% (91/131)	Initial neurological exam performed within 24 hours on all patients.<br><br>**Baseline AIS grade, % (n/N)**<br>Early:<br>A: 35.7% (65/182)<br>B: 22.0% (40/182)<br>C: 17.6% (32/182)<br>D: 24.7% (45/182)<br>Late:<br>A: 27.5% (36/131)<br>B: 10.7 % (14/131)<br>C: 26.0% (34/131)<br>D: 35.9% (47/131)<br><br>**Baseline Charlson Comorbidity Index ≥1, % (n/N)**<br>Early: 22.0% (40/182)<br>Late: 26.0% (30/131)	**Cause of trauma, % (n/N)**<br>Early<br>MVA: 41.8% (76/182)<br>Fall: 35.1% (64/182)<br>Assault-blunt: 4.4% (8/182)<br>Sports: 8.8% (16/182)<br>Other: 9.9% (18/182)<br>Late<br>MVA: 32.8% (43/131)<br>Fall: 43.5% (57/131)<br>Assault-blunt: 3.8% (5/131)<br>Sports: 9.2% (12/131)<br>Other: 10.7% (14/131)	**Interventions**<br>• Decompression accompanied by an instrumented fusion procedure.<br>• Approach (anterior vs. posterior) and number of levels decompressed at the discretion of the spinal surgeon.<br><br>**Co-interventions**<br>• 194 (62.0%) patients received steroids at hospital admission; significantly higher proportion	From time of injury to treatment.<br><br>**Time from injury to surgery, mean ± SD**<br>Early: 14.2 ± 5.4 hours<br>Late: 48.3 ± 29.3 hours	**Inclusion:**<br>• Male or female, ages 16-80, initial GCS > 13, initial AIS grade A-D, cervical spinal cord compression confirmed by MRI or CT myelography, patient or Proxy willing to provide consent for enrollment, neurological level of injury between C2-T1 .<br><br>**Exclusion:**<br>• Cognitive impairment preventing accurate neurologic assessment, penetrating injuries to the neck, pregnancy, pre-injury major neurologic deficits or disease (i.e. ischemic stroke, Parkinson's disease), life threatening

				**Baseline GCS (mean ± SD)**<br><u>Early:</u> 14.9 ± 0.4<br><u>Late:</u> 14.9 ± 0.4		administered in the early vs. the late group (*P* = .04).<br>• All patients received appropriate medical support according to 2002 AANS cervical SCI guidelines.<br>• Methylprednisolone used as per the discretion of the treating team.<br>• All patients underwent a post-operative rehabilitation regimen, tailored to individual and injury specific factors.		injuries which prevent early decompression of the spinal cord, arrival at health center >24 hours after SCI, surgery >7 days after SCI<br><br>**<u>Adjusted for:</u>**<br>• Stratified by baseline AIS grade changes.<br>• Pre-operative neurological status and steroid administration.
Lee (2021)<br><br>Cervical SCI<br><br>Incomplete	Retrospective cohort (Poor)	**<u>Early (≤24 h)</u>**<br>N = 33<br>Mean age ± SD: 57.4 ± 14.0 years<br>Male: 78.8%<br><br>**<u>Conservative treatment</u>**<br>N = 21<br>Mean age ± SD: 56.9 ± 13.6	6 months (100.0%; n = 54/54)<br><br><u>Early</u>: 100.0% (n = 33/33)<br><br><u>Late</u>: 100.0% (n = 21/21)	Initial neurologic examinations NR<br><br>**Baseline AIS, % (n/N)**<br><u>Early:</u><br>B: 23.8% (6/33)<br>C: 76.2% (27/33)<br><u>Conservative:</u><br>B: 18.2% (5/21)<br>C: 81.8% (16/21)	**Cause of trauma, % (n/N)**<br><u>Early</u><br>Traffic accident: 30.3% (10/33)<br>Falling: 24.2% (8/33)<br>Slip down: 21.2% (7/33)<br>Sports: 9.1% (3/33)<br>Others: 15.2%	**<u>Interventions</u>**<br>• Cervical spinal fusion and decompression surgery through a posterior approach without methylprednisolone (surgery group)<br>• Received high	From initial trauma to surgical treatment<br><br>**<u>Mean time from injury to surgery</u>**<br>NR	**<u>Inclusion:</u>**<br>• Pre-existing cervical spinal canal stenosis with SCI grade B or C, but without major fracture or dislocation.<br><br>**<u>Exclusion:</u>**<br>• Patients with vertebral body fracture, lamina fracture, facet fracture, dislocation, traumatic

		years<br>Male: 81.0%			<u>Conservative</u><br>Traffic accident: 28.6% (6/21)<br>Falling: 28.6 (6/21)<br>Slip down: 23.8% (5/21)<br>Sports: 9.5% (2/21)<br>Others: 9.5% (2/21)	dose of methylprednisolone, and instructed to limit cervical motion and stay in bed for at least 1 or 2 weeks (Conservative group)<br><br>**<u>Co-interventions</u>**<br>NR		herniated intervertebral disk, follow-up <2 years, co-occurrence of another significant injury, or because they had incomplete study protocols.<br><br>**<u>Adjusted for:</u>**<br>• adjusted for age, sex, cause of trauma, canal compression rate, spinal canal diameter, baseline AIS grade, and treatment type for early (≤24 h) vs. conservative treatment.<br>• AIS outcome stratified by baseline AIS (baseline AIS grade B and C only).
Umerani (2014)<br><br>Cervical SCI<br><br>Complete/incomplete	Prospective cohort (Fair)	**<u>Early (≤24 h)</u>**<br>N = 34<br>Mean age (range): 37.5 (21 to 65) years<br>Male: 82.35%<br><br>**<u>Late (>24 h)</u>**<br>N = 64<br>Mean age (range): 40.1 (19 to 61) years<br>Male: 76.56%	6 months<br>93.9% (92/98)<br><br><u>Early</u>: 91.2% (31/34)<br><br><u>Late</u>: 95.3% (61/64)	Initial neurologic examinations on admission.<br><br>**Baseline AIS grade, % (n/N)**<br><u>Early:</u><br>A: 38.2% (13/34)<br>B: 11.8% (4/34)<br>C: 29.4% (10/34)<br>D: 20.6% (7/34)<br><u>Late:</u><br>A: 35.9% (23/64)<br>B: 12.5% (8/64)<br>C: 21.9% (14/64)<br>D: 29.7% (19/64)	**Cause of trauma, % (n/N)**<br><u>Early:</u><br>Traffic accidents: 41.2% (14/34)<br>Fall: 29.4% (10/34)<br>Assault: 23.5% (8/34)<br>Other: 5.9% (2/34)<br><u>Late:</u><br>Traffic accidents: 60.9% (39/64)<br>Fall: 18.8% (12/64)<br>Assault: 15.6% (10/64)<br>Other: 4.7%	**<u>Interventions</u>**<br>• Surgical decompression and fusion.<br><br>**<u>Co-interventions</u>**<br>NR	From time of trauma to surgery.<br><br>**<u>Time from injury to surgery, mean (range)</u>**<br><u>Early:</u> 18.4 (13 to 24) hours<br><u>Late:</u> 52.7 (31 to 124) hours	**<u>Inclusion:</u>**<br>• Patients presenting with cervical cord injury from C3 to T1, aged between 18-65 years.<br><br>**<u>Exclusion:</u>**<br>• Patients with GCS of <14 or baseline AIS grade E.<br><br>**<u>Adjusted for:</u>**<br>• AIS outcome stratified by baseline AIS grade.

					(3/64)			
Badhiwala (2021)[*]<br><br>Cervical SCI, thoracic SCI, Lumbosacral SCI[†]<br><br>Complete/incomplete	Pooled individual patient data from 4 cohorts (NACTN SCI, STASCIS, Sygen Trial, NASCIS III) (Good)	<u>**Early (<24 h)**</u><br>N = 528<br>Mean age ± SD: 39.5 ± 16.9 years<br>Male: 79.0%<br><br><u>**Late (≥24 h)**</u><br>N = 1020<br>Mean age ± SD: 38.9 ± 17.0 years<br>Male: 79.9%	12 months (% NR)	Initial neurologic exam varied by dataset.<br><br>**Baseline AIS grade, % (n/N)**<br><u>Early:</u><br>A: 49.2% (260/528)<br>B: 15.5% (82/528)<br>C: 16.7% (88/528)<br>D: 18.6% (98/528)<br><u>Late</u>:<br>A: 49.6% (506/1020)<br>B: 11.5% (117/1020)<br>C: 17.7% (181/1020)<br>D: 21.2% (216/1020)<br><br>**Baseline AMS, mean ± SD**<br><u>Early:</u> 32.8 ± 27.4<br><u>Late:</u> 36.1 ± 28.8<br><br>**Baseline Light touch score, mean ± SD**<br><u>Early:</u> 53.2 ± 34.8<br><u>Late:</u> 54.8 ± 35.3<br><br>**Baseline Pin prick score, mean ± SD**<br><u>Early:</u> 49.1 ± 34.5<br><u>Late:</u> 50.9 ± 34.6	**Mechanism of injury, % (n/N)**<br><u>Early</u><br>Fall: 32.4% (171/528)<br>Motor vehicle collision: 43.6% (230/528)<br>Sports injury: 10.2% (54/528)<br>Other: 13.8% (73/528)<br><u>Late</u><br>Fall: 28.3% (289/1020)<br>Motor vehicle collision: 47.5% (484/1020)<br>Sports injury: 10.0% (102/1020)<br>Other: 14.2% (145/1020)<br><br>**Level of injury, % (n/N)**<br><u>Early:</u><br>Cervical: 86.9% (459/528)<br>Thoracic: 10.2% (54/528)<br>Lumbosacral: 2.8% (15/528)<br><u>Late:</u><br>Cervical: 80.0%	<u>**Interventions**</u><br>• Surgical decompression.<br><br><u>**Co-interventions**</u><br>• In the NASCIS III trial, patients were randomized to receive methylprednisolone at 24 hours, methylprednisolone at 48 hours, or tirilazad at 48 hours.	From time of injury to decompression.<br><br><u>**Time from injury to surgery, median (IQR)**</u><br><u>Early:</u> 13 (9 to 18) hours<br><u>Late:</u> 69 (41 to 135) hours	<u>**Inclusion:**</u><br>• All patients with acute SCI who received surgical decompression found in any one of four datasets.<br><br><u>**Exclusion:**</u><br>NR<br><br><u>**Adjusted for:**</u><br>• Baseline score, age, mechanism of injury, baseline AIS grade, spinal level of injury, and administration of methylprednisolone.

					(816/1020)<br>Thoracic: 17.2% (175/1020)<br>Lumbosacral: 2.8% (29/1020)			
**Thoracolumbar SCI**								
Rahimi-Movaghar (2014)<br><br>Thoracolumbar tSCI<br><br>Complete/incomplete<br><br>From prior report	RCT (Fair)	**<u>Early (≤24 h)</u>**<br>N = 16<br>Mean age ± SD: 31.7 ± 9.1 years<br>Male: 69.0%<br><br>**<u>Late (24-72 h)</u>**<br>N = 19<br>Mean age ± SD: 37.8 ± 13.70 years<br>Male: 74.0%	<u>Early:</u><br>1 month follow-up: 87.5% (14/16)<br>3 months follow-up: 56.3% (9/16)<br>6 months follow-up: 87.5% (14/16)<br>12 months follow-up: 93.8% (15/16)<br><br><u>Late:</u><br>1 month follow-up: 73.7% (14/19)<br>3 months follow-up: 63.2% (12/19)<br>6 months follow-up: 78.9% (15/19)	Initial neurologic examinations performed on admission, preoperatively, immediately after surgery, and at one, 3, 6, and 12-months follow-ups.<br><br>**Baseline AIS grade, % (n/N)**<br><u>Early:</u><br>A: 44.0% (7/16)<br>B: 6.0% (1/16)<br>C: 25.0% (4/16)<br>D: 25.0% (4/16)<br><u>Late:</u><br>A: 47.0% (9/19)<br>B: 26.0% (5/19)<br>C: 5.0% (1/19)<br>D: 21.0% (4/19)<br><br>**Baseline AMS, mean ± SD**<br><u>Early:</u> 77 ± 22<br><u>Late:</u> 68 ± 22	**Cause of trauma % (n/N)**<br><u>Early:</u><br>Automobile crashes: 25% (4/16)<br>Motorcycle crashes: 19% (3/16)<br>Fall: 44% (7/16)<br>Other: 12% (2/16)<br><u>Late:</u><br>Automobile crashes: 74% (14/19)<br>Motorcycle crashes: 10% (2/19)<br>Fall: 16% (3/19)<br>Other: 0% (0/19)	**<u>Interventions</u>**<br>• Decompression accompanied by spinal fusion and fixation.<br><br>**<u>Co-interventions</u>**<br>• Standard spinal immobilization and resuscitation techniques.<br>• Intravenous methylprednisolone based on recommendations from National Acute Spinal Cord Injury Studies (NASCIS).<br>• Gastrointestinal prophylaxis.	From time of injury to decompression.<br><br>**<u>Time from injury to surgery, mean ± SD</u>**<br><u>Early:</u> 18.9 ± 4.75 hours<br><u>Late:</u> 45.0 ± 11.93	**<u>Inclusion:</u>**<br>• >18 years old, traumatic SCI between T1 – L1, hemodynamic stability, evidence of spinal cord/conus medullaris compression and/or MRI signal change, hospital admission before 24 hours of injury.<br><br>**<u>Exclusion:</u>**<br>• American Spinal Injury Association (ASIA) Impairment Scale (AIS) grade of E, no cord compression on MRI, spinal shock, injury involving more than 2 adjacent vertebral levels, Inability to provide informed consent, any cognitive deficit, major and current psychiatric illness, significant concurrent traumatic brain injury, major concurrent medical disease, pre-injury major neurologic deficits or disease, ankylosing

			12 months follow-up: 94.7% (18/19)					• spondylitis, penetrating thoracolumbar injuries, pregnancy, life-threatening injuries preventing early cord decompression, criminals under indictment, incarceration, substance abuse.<br><br>**Adjusted for:**<br>• AIS outcome stratified by baseline AIS grade.
Qadir (2020)<br><br>Thoracolumbar SCI<br><br>Complete/incomplete	Retrospective cohort (Poor)	**Early (<24 h)**<br>N = 144<br>Mean age ± SD: 30.5 ± 12.0 years<br>Male: 68.1%<br><br>**Intermediate (24-72 h)**<br>N = 77<br>Mean age ± SD: 33.5 ± 13.0 years<br>Male: 71.4%<br><br>**Late (>72 h)**<br>N = 96<br>Mean age ± SD: 31.6 ± 12.5 years<br>Male: 75.0%	12 months 100% (317/317)<br><br>Early: 100% (144/144)<br><br>Intermediate: 100% (77/77)<br><br>Late: 100% (96/96)	Initial neurologic examinations performed on admission.<br><br>**Baseline AIS grade, % (n/N)**<br>Early:<br>A: 59.7% (86/144)<br>B: 16.0% (23/144)<br>C: 16.7% (24/144)<br>D: 7.6% (11/144)<br>Intermediate:<br>A: 59.7% (46/77)<br>B: 7.8% (6/77)<br>C: 24.7% (19/77)<br>D: 7.8% (6/77)<br>Late:<br>A: 65.6% (63/96)<br>B: 7.3% (7/96)<br>C: 15.6% (15/96)<br>D: 11.5% (11/96)	**Neurological level of injury, % (n/N)**<br>Early:<br>D1 1: 2.8% (4/144)<br>D1 2: 37.5% (54/144)<br>L1: 42.4% (61/144)<br>L2: 17.4% (25/144)<br>Intermediate:<br>D1 1: 7.8% (6/77)<br>D1 2: 22.1% (17/77)<br>L1: 50.6% (39/77)<br>L2: 19.5% (15/77)<br>Late:<br>D1 1: 7.3% (7/96)<br>D1 2: 42.7% (41/96)<br>L1: 41.7% (40/96)<br>L2: 8.3% (8/96)	**Interventions**<br>• Decompression accompanied by spinal fusion and fixation.<br><br>**Co-interventions**<br>• Decompression accompanied by spinal fusion and fixation.	From time of injury to decompression.<br><br>**Time from injury to surgery, mean ± SD**<br>NR	**Inclusion:**<br>• Non-penetrating traumatic SCI (AIS A-D) at the thoracolumbar junction (T11 to L2), >14 years old, complete initial and 1-year ASIA examinations.<br><br>**Exclusion:**<br>• Patients with cauda equina, peripheral nerve injuries, cases where physical examinations were not reliable because of concurrent injuries, fractures involving L3 to L5 level.<br><br>**Adjusted for:**<br>• AIS outcome stratified by baseline AIS grade.

**Thoracic SCI**								
Haghnegahdar (2020)<br><br>Thoracic SCI<br><br>Complete/incomplete<br><br>Complete data of Rahimi-Movaghar 2014	RCT (Fair)	**<u>Early (<24 h)</u>**<br>N = 37<br>Mean age ± SD: 29.7 ± 10.3 years<br>Male: 75.7%<br><br>**<u>Late (24-72 h)</u>**<br>N = 36<br>Mean age ± SD: 34.9 ± 12.0 years<br>Male: 72.2%	12 months: 92.4% (73/79)<br><br><u>Early</u>: 94.8% (37/39)<br><br><u>Late</u>: 92.3% (36/39)	Initial neurologic exam was performed first by a junior resident at admission and then again by senior resident or local PI at least 1 hour prior to surgery.<br><br>**Baseline AIS grade, % (n/N)**<br><u>Early:</u><br>A: 56.8% (21/37)<br>B: 13.5% (5/37)<br>C: 10.8% (4/37)<br>D: 18.9% (7/37)<br><u>Late:</u><br>A: 55.6% (20/36)<br>B: 13.9% (5/36)<br>C: 11.1% (4/36)<br>D: 19.4% (7/36)<br><br>**Neurological level number, % (n/N)**<br><u>Early:</u><br>T1-4: 2.7% (1/37)<br>T5-8: 13.5% (5/37)<br>T9-L1: 83.8% (31/37)<br><u>Late:</u><br>T1-4: 11.1% (4/36)<br>T5-8: 19.4% (7/36)<br>T9-L1: 69.4% (25/36)<br><br>**Baseline AMS, %**	**Mechanism of injury, % (n/N)**<br><u>Early:</u><br>MVA: 45.9% (17/37)<br>Falls: 48.7% (18/37)<br>Sport 0% (0/37)<br>Other: 5.4% (2/37)<br>Missing: 0% (0/37)<br><u>Late:</u><br>MVA: 66.7% (24/36)<br>Falls: 30.6% (11/36)<br>Sport 0% (0/36)<br>Other: 0% (0/36)<br>Missing: 2.8% (1/36)	**<u>Interventions</u>**<br>• Surgical decompression.<br><br>**<u>Co-interventions</u>**<br>• Patients infused with methylprednisolone sodium succinate.	From time of injury to presentation in ER.<br><br>**<u>Time from injury to surgery, mean ± SD</u>**<br>NR	**<u>Inclusion:</u>**<br>• Patients ≥16 years with acute traumatic thoracic and thoracolumbar SCI (T1-L1) that were hemodynamically stable, evident of spinal cord compression on MRI, presenting less than 24 hours since injury.<br><br>**<u>Exclusion:</u>**<br>• Patients with concomitant traumatic brain injury, pre-injury comorbidities or neurological deficits, psychiatric illness, ankylosing spondylitis, penetrating SCI, life-threatening injuries that prevent decompression, pregnancy, criminality, spinal shock, cognitive impairment preventing accurate neurological assessment, or injury involving more than 2 adjacent vertebral levels.<br><br>**<u>Adjusted for:</u>**<br>• AIS outcome stratified by baseline AIS grade.<br>• Mean AMS adjusted for

				**(n/N)**<br>Early: 62.3 ± 15.6<br>Late: 58.1 ± 14.1				baseline neurological status
**Mixed SCI**								
Bourassa-Moreau (2013)<br><br>Cervical, thoraco-lumbar SCI<br><br>Complete/incomplete<br><br>From prior report	Retrospective cohort (Fair)	**Early (≤24 h)**<br>N = 90<br>Mean age ± SD: 37.0 ± 15.9 years<br>Males: 82.2%<br><br>**Late I (25-72 h)**<br>N = 231<br>Mean age ± SD: 40.7 ± 17.3 years<br>Males: 78.4%<br><br>**Late II (>72 h)**<br>N = 110<br>Mean age ± SD: 47.9 ± 18.0 years<br>Males: 72.7%	Mean NR; follow-up period included the acute hospital stay	Timing of initial neurological exam NR.<br><br>**Baseline grade, % (n/N)**<br>Early:<br>A: 61.1% (55/90)<br>B: 17.8% (16/90)<br>C: 8.9% (8/90)<br>D: 12.2% (11/90)<br>Late I:<br>A: 47.2% (109/231)<br>B: 18.2% (42/231)<br>C: 16.5% (38/231)<br>D: 18.2% (42/231)<br>Late II:<br>A: 30.0% (33/110)<br>B: 11.8% (13/110)<br>C: 13.6% (15/110)<br>D: 44.5% (49/110)<br><br>**Baseline Charlson Comorbidity Index (mean ± SD)**<br>Overall: 0.22 ± 0.68<br>Early: 0.10 ± 0.37<br>Late I: 0.19 ± 0.60<br>Late II: 0.38 ± 0.96<br><br>**Baseline ISS (mean ± SD)**<br>Overall: 26.2 ± 10.2	• Spinal fracture, dislocation or fracture-dislocation from C1-L2, with clinical evidence of SCI<br><br>**Paraplegia, % (n/N)**<br>Early: 67.8% (61/90)<br>Late I: 56.3% (130/231)<br>Late II: 32.7% (36/110)<br><br>**Traumatic Brain Injury, % (n/N)**<br>Early: 36.7% (33/90)<br>Late I: 37.7% (76/231)<br>Late II: 28.2% (31/110)	**Intervention**<br>• Surgical decompression.<br><br>**Co-interventions**<br>NR	From time of injury to time of skin incision.<br><br>**Time from injury to surgery, mean ± SD**<br>NR	**Inclusion:**<br>• Spinal fracture, dislocation or fracture-dislocation from C1 to L2, clinical evidence of SCI (ASIA A, B, C, and D), minimal age of 16 years, spine surgery performed at our center.<br><br>**Exclusion:**<br>• Penetrating trauma to the spine, nonsurgical management, central cord syndrome or absence of acute spine injury, unknown neurologic assessment, associated neurologic disorders that preclude neurologic assessment including severe traumatic brain injury.<br><br>**Adjusted for:**<br>• Age, sex, Charlson Co-morbidity Index, neurological level of injury, ISS, presence of mild or moderate traumatic brain injury, and surgical invasiveness index.

				Early: 28.2 ± 10.2<br>Late I: 26.6 ± 10.0<br>Late II: 23.5 ± 10.3<br><br>**Surgical Invasiveness Index (mean ± SD)**<br>Overall: 12.2 ± 7.2<br>Early: 12.5 ± 7.3<br>Late I: 12.7 ± 7.3<br>Late II: 10.9 ± 7.0				
Bourassa-Moreau (2016)<br><br>Cervical SCI, thoracolumbar SCI<br><br>Complete	Prospective cohort (Fair)	**Early (≤24 h)**<br>N = 38<br>Mean age ± SD: 39.6 ± 16.6 years<br>Male: 89%<br><br>**Late (>24 h)**<br>N = 15<br>Mean age ± SD: 49.6 ± 15.4 years<br>Male: 73%	Mean 5 months (% NR)	Initial neurologic exam at arrival to the trauma center and at rehabilitation discharge.<br><br>**Baseline GCS, mean ± SD**<br>Early: 13.8 ± 2.5<br>Late: 13.7 ± 2.4<br><br>**Baseline ISS, mean ± SD**<br>Early: 32.1 ± 10.8<br>Late: 34.4 ± 14.1	**BMI, Mean ± SD**<br>Early: 26.3 ± 3.9<br>Late: 26.0 ± 4.0<br><br>**Proportion with comorbidities, % (n/N)**<br>Early: 26% (10/38)<br>Late: 40% (6/15)<br><br>**Proportion of nonsmokers, % (n/N)**<br>Early: 76% (29/38)<br>Late: 67% (10/15)<br><br>**Follow-up (days), mean ± SD**<br>Early: 150.6 ± 39.7<br>Late: 156.9 ± 31.2	**Interventions**<br>• Surgical decompression.<br><br>**Co-interventions**<br>• Cervical traction in the presence of cervical dislocation or significant cervical misalignment causing spinal cord compression, unless surgery is to be performed within 1 hour.	From time of injury to surgical incision.<br><br>**Time from injury to surgery, mean ± SD**<br>Early: 16.1 ± 4.9 hours<br>Late: 39.1 ± 16.3 hours	**Inclusion:**<br>• Patients ≥16 years with AIS A traumatic SCI with vertebral fracture and/or luxation from C1 to L2.<br><br>**Exclusion:**<br>• Patients with neurological or cognitive impairment, surgical intervention performed in previous 3 days, or surgical decompression or fusion performed in another center.<br><br>**Adjusted for:**<br>• Stratified by SCI type<br>• Stratified by age <40 and ≥40 years.
Dvorak (2015)<br><br>Cervical, thoracic, lumbosacral SCI	Prospective cohort (Poor)	**Early (≤24 hours)**<br>N = 355 (40% patients)<br>Average age: NR<br>Male: NR	Mean NR; 'Final' ISNCSCI assessments were generally performed	Initial neurologic exam performed within 72 hours post-injury.<br><br>**Baseline AIS grade, % (n/N)**	**Neurological level of injury, % (n/N)**<br>Overall<br>High cervical (C1-C4): 26.5% (NR)<br>Low cervical (C5-T1): 35.8% (NR)	**Interventions**<br>• Combination of either stabilization and/or decompression	From time of injury to treatment.<br><br>**Time from injury to surgery, mean**	**Inclusion:**<br>• Participants in the RHSCIR, acute traumatic SCI, surgery <1 month post-injury.<br><br>**Exclusion:**

Complete/incomplete<br><br>From prior report		**<u>Late (25-168 h)</u>**<br>N = 533 (60% patients)<br>Average age: NR<br>Male: NR	between 3-6 months following injury."	<u>Overall</u><br>A: 38.8% (NR)<br>B: 12% (NR)<br>C: 18.4% (NR)<br>D: 30.9% (NR)	Thoracic (T2-T10): 16.7% (NR)<br>Thoracolumbar (T11-L2): 21.0% (NR)	**<u>Co-interventions</u>**<br>NR	**<u>± SD</u>**<br><u>Total:</u> 60.4 ± 80 hours	• GCS <14, timing of surgery and neurological examinations unspecified, failure to provide consent to collection of baseline and follow-up neurological examination results.<br><br>**<u>Adjusted for:</u>**<br>• AIS outcome stratified by baseline AIS grade.
Wilson (2012)<br><br>Cervical, thoracic, lumbosacral SCI<br><br>Complete/incomplete<br><br>From prior report	Prospective cohort (Fair)	**<u>Early (<24 h)</u>**<br>N = 35<br>Mean age: 41.6 years<br>Males: 83%<br><br>**<u>Late (≥24 h)</u>**<br>N = 49<br>Mean age: 47.9 years<br>Males: 78.0%	**Acute care discharge**, **mean:** 24.8 ± 29.2 days, 97.6% (82/84)<br><br>**Inpatient rehabilitation discharge, mean ± SD:** 89.6 ± 47.4 days 65.4% (55/84)<br><u>Early:</u> 62.9% (22/35)<br><u>Late:</u> 67.3% (33/49)	Initial assessment at acute-care admission<br><br>**Baseline AIS grade, % (n/N)**<br><u>Early:</u><br>A: 51% (18/35)<br>B: 17% (6/35)<br>C: 14% (5/35)<br>D: 17% (6/35)<br><u>Late:</u><br>A: 31% (15/49)<br>B: 6% (3/49)<br>C: 12% (6/49)<br>D: 51% (25/49)	**Neurological level of injury, % (n/N)**<br><u>Early</u><br>Cervical: 40% (14/35)<br>Thoracic: 34.3% (12/35)<br>Lumbosacral: 25.7 (9/35)<br><u>Late</u><br>Cervical: 61.2% (30/49)<br>Thoracic: 18.4% (9/49)<br>Lumbosacral: 20.4% (10/49)<br><br>**Cause of trauma, % (n/N):**<br><u>Early:</u><br>MVA: 37.1% (13/35)<br>Fall: 37.1% (13/35)<br>Assault: 2.9%	**<u>Interventions</u>**<br>• Approach, extent of decompression and use of spinal instrumentation made on a case-by-case basis by the attending orthopedic or neurosurgeon.<br><br>**<u>Co-interventions</u>**<br>• All patients received optimal medical support, which included permissive or induced hypertensive therapy for 1 week following the injury.<br>• Methylprednisolone was used as	From timing of SCI to surgery.<br><br>**<u>Time from injury to surgery, mean ± SD</u>**<br><u>Early:</u> 12.7 ± 4.9 hours<br><u>Late:</u> 155.0 ± 236.7 hours	**<u>Inclusion:</u>**<br>• Traumatic SCI, age > 16, initial ASIA impairment scale grade A-D, spinal cord compression confirmed by MRI or CT myelography, patient or proxy willing to provide consent for enrollment.<br><br>**<u>Exclusion:</u>**<br>• Cognitive impairment preventing accurate neurological assessment, penetrating injuries, pregnancy, pre-injury major neurological deficits or disease (i.e. ischemic stroke, Parkinson's disease), life-threatening injuries that prevent early decompression of the spinal cord, significant pre-morbid medical

					(1/35)<br>Other: 22.9% (8/35)<br>Late:<br>MVA: 20.4% (10/49)<br>Fall: 59.2% (29/49)<br>Assault: 6.1% (3/49)<br>Other: 14.3% (7/49)<br><br>**Received methylprednisolone, % (n/N)**<br>Early: 12% (3/25)<br>Late: 19.4% (7/36)<br><br>All patient underwent an individualized post-op rehab protocol in a spinal cord rehab unit	per the discretion of the treating team according to the recommendations of the Second National Acute Spinal Cord Injury Study.		illness, including but not limited to: myocardial infarction within 3 months; uncompensated heart failure; active systemic cancer; AIDS.<br><br>**Adjusted for:**<br>• Adjusted for surgical timing, baseline AIS grade, and neurological level of injury.
Du (2018)<br><br>Thoracic SCI, Thoracolumbar SCI<br><br>Incomplete	Prospective cohort (Fair)	**Early (<24 h)**<br>N = 335<br>Mean age ± SD: 43.4 ± 13.9 years<br>Males: 70.4%<br><br>**Late (24-72 h)**<br>N = 386<br>Mean age: 47.9 ± 14.2 years	12 months: 100% (721/721)<br><br>Early: 100% (335/335)<br><br>Late: 100% (386/386)	Initial assessment performed within 12 hours of admission.<br><br>**Baseline AIS grade, % (n/N)**<br>Early:<br>B: 24.8% (83/335)<br>C: 34.3% (115/335)<br>D: 40.9% (137/335)<br>Late:	**Neurological level of injury, % (n/N)**<br>Early:<br>Thoracic: 58.5% (196/335)<br>Thoracolumbar: 41.5% (139/335)<br>Late:<br>Thoracic: 56.7% (219/386)<br>Thoracolumbar:	**Interventions**<br>• Surgical decompression.<br><br>**Co-interventions**<br>NR	From timing of injury to decompression.<br><br>**Time from injury to surgery, mean ± SD**<br>Early: 18.0 ± 3.8 hours	**Inclusion:**<br>• 16-18 years old, incomplete SCI with an initial AIS grade of B-D, spinal cord compression or injury confirmed with magnetic resonance imaging or CT myelography, informed consent from patients, thoracic/thoracolumbar

		Males: 68.9%		B: 32.1% (124/386)<br>C: 24.9% (96/386)<br>D: 43.0% (166/386)<br><br>**Injury Severity Score (mean ± SD)**<br>Early: 17.6 ± 6.1<br>Late: 18.4 ±7.0	43.3% (167/386)<br><br>**Cause of trauma, % (n/N):**<br>Early:<br>Motor vehicle: 61.2% (205/335)<br>Fall: 26.3% (88/335)<br>Other: 12.5% (42/335)<br>Late:<br>Motor vehicle: 65.5% (253/386)<br>Fall: 24.9% (96/386)<br>Other: 9.6% (37/386)<br><br>**Received methylprednisolone % (n/N):**<br>Early: 28.9% (97/335)<br>Late: 23.6% (91/386)		Late: 43.4 ± 12.9 hours	SCI level at T1-L1, indication for surgery as defined by thoracolumbar injury severity score of 4 or greater.<br><br>**Exclusion:**<br>• Major neurologic deficits or illness before injury, serious life-threatening injury that is not early cord decompression, vertebral infection, tumors, or ankylosing spondylitis, penetrating thoracolumbar injuries, pregnancy, arrival at orthopedic trauma center more than 24 hours after thoracic/thoracolumbar incomplete SCI or surgery or more than 72 hours after SCI, injury along with cervical fractures or multiple system injuries, or injury involving more than 2 adjacent vertebral levels.<br><br>**Adjusted for:**<br>• AIS outcome stratified by baseline AIS grade.
Ter Wengel (2022)<br><br>Cervical SCI,	Retrospective cohort (Fair)	**Early (<24 h)**<br>N = 82<br>Mean age ± SD: 49.7 ± 18.1	NR, follow-up ended at discharge from	Initial assessment performed at admission	**Neurological level of injury, % (n/N)**<br>Early:<br>Cervical: 59.8%	**Interventions**<br>• Surgical decompression.	From timing of admission to surgery, with additional	**Inclusion:**<br>• Patients with traumatic AIS A or B injuries from C2-L2.

Thoracic SCI, Thoracolumbar SCI<br><br>Complete/Incomplete		years<br>Males: 80.5%<br><br>**<u>Late (≥24 h)</u>**<br>N = 14<br>Mean age: 50.2 ± 20.8 years<br>Males: 71.4%	rehabilitation center.	**Baseline AIS grade, % (n/N)**<br><u>Early:</u><br>A: 63.4% (52/82)<br>B: 36.6% (30/82)<br><u>Late:</u><br>A: 85.5% (12/14)<br>B: 14.3% (2/14)<br><br>**Baseline AO classification**<br><u>Early:</u><br>A: 8.5% (7/82)<br>B: 41.5% (34/82)<br>C: 50.0% (41/82)<br><u>Late:</u><br>A: 14.3% (2/14)<br>B: 57.1% (8/14)<br>C: 28.6% (4/14)	(49/82)<br>Thoracic: 19.5% (16/82)<br>Thoracolumbar: 20.7% (17/82)<br><u>Late:</u><br>Cervical: 71.4% (10/14)<br>Thoracic: 14.3% (2/14)<br>Thoracolumbar: 14.3% (2/14)<br><br>**Cause of trauma, % (n/N):**<br><u>Early:</u><br>High energy trauma: 61.0% (50/82)<br>Low energy trauma: 31.7% (26/82)<br>Other: 7.3% (6/82)<br><u>Late:</u><br>High energy trauma: 71.4% (10/14)<br>Low energy trauma: 21.4% (3/14)<br>Other: 7.1% (1/14)	**<u>Co-interventions</u>**<br>NR	average of 50 minutes for timing of injury to admission<br><br>**<u>Time from injury to surgery, mean ± SD</u>**<br><u>Early:</u> 7.9 ± 5.3 hours<br><u>Late:</u> 151.2 ± 196.4 hours	**<u>Exclusion:</u>**<br>• No surgical treatment, age <15 years, <M6 in the GCS, presence of voluntary anal contraction, life-threatening injuries which prevented initial examination and subsequent spinal surgery, gunshot or stab injuries, clear transection of the spinal cord seen with MR images or intraoperatively.<br><br>**<u>Adjusted for:</u>**<br>• Adjusted for surgical timing, level of injury, baseline AIS, and AO classification.

AANS = American Association of Neurological Surgeons; ACDF = Anterior cervical discectomy and fusion; ACCF = Anterior cervical corpectomy and fusion; AIDS = acquired immunodeficiency syndrome; AIS = ASIA Impairment Scale; AMS = ASIA Motor Score; ASIA = American Spinal Injury Association; CCS = central cord syndrome; CI = confidence interval; CT = computed tomography; GCS = Glasgow Coma Scale; ICU = intensive care unit; IQR = interquartile range; ISNCSCI = International Standards for Neurological Classification of Spinal Cord Injury; ISS = Injury Severity Score; MRI = magnetic resonance imaging; MVA = motor vehicle accident; NR = not reported; RHSCIR = Rick Hansen Spinal Cord Registry; OR = odds ratio; SCI = spinal cord injury; SD = standard deviation; STASCIS = Surgical Timing in Acute Spinal Cord Injury Study; tSCI = traumatic spinal cord injury.

* There is some overlap between Badhiwala (2021) and Fehlings (2012). However, Fehlings' outcomes are reported at 6 month follow-up, while Badhiwala continues follow-up to 1 year. Badhiwala does not report n/Ns at 1 year, only proportions.
† 83% of sample is Cervical SCI.

Table D2. Study characteristics and patient demographics for studies comparing other surgical timings

Author (year)<br>Injury<br>Injury level	Study design (Quality)	Demographics	Follow-up (% followed)	Baseline neurological, disease, and trauma severity status	Patient characteristics	Intervention(s) and Co-intervention(s)	Timing of treatment	Inclusion/Exclusion<br><br>Adjustment for baseline neuro-status
**Cervical SCI**								
Jug (2015)<br><br>Cervical SCI<br><br>Complete/incomplete	Prospective cohort (Fair)	**<u>Early (<8 h)</u>**<br>N = 22<br>Median age (IQR): 44.0 years (30.5 to 58.5)<br>Male: 82.0%<br><br>**<u>Late (8-24 h)</u>**<br>N = 20<br>Median age (IQR): 52.0 years (25.8 to 72.8)<br>Male: 80.0%	6 months: 100% (42/42)<br><br><u>Early</u>: 100% (22/22)<br><br><u>Late</u>: 100% (20/20)	Initial neurologic exam on admission.<br><br>**Baseline AIS grade, % (n/N)**<br><u>Early:</u><br>A: 59% (13/22)<br>B: 23% (5/22)<br>C: 18% (4/22)<br><u>Late:</u><br>A: 65% (13/20)<br>B: 5% (1/20)<br>C: 30% (6/20)<br><br>**Spinal canal compromise %, median (IQR)**<br><u>Early:</u> 50 (30 to 50)<br><u>Late:</u> 30 (30 to 50)<br><br>**Spinal injury pattern, % (n/N)**<br><u>Early:</u><br>A: 18% (4/22)<br>B: 23% (5/22)<br>C: 59% (13/22)<br><u>Late:</u>	**Smokers, % (n/N)**<br><u>Early:</u> 27% (6/22)<br><u>Late:</u> 35% (7/20)<br><br>**Comorbidities, % (n/N)**<br><u>Early:</u> 14% (3/22)<br><u>Late:</u> 35% (7/20)<br><br>**Mechanism of injury, % (n/N)**<br><u>Early:</u><br>Assault: 9% (2/22)<br>Diving: 14% (3/22)<br>Fall 27% (6/22)<br>MVA: 36% (8/22)<br>Sport: 14% (3/22)<br><u>Late:</u><br>Assault: 0% (0/20)<br>Diving: 15% (3/20)<br>Fall 50% (10/20)<br>MVA: 35% (7/20)<br>Sport: 0% (0/20)	**<u>Interventions</u>**<br>• Surgical decompression.<br><br>**<u>Co-interventions</u>**<br>NR	From time of injury to decompression (for dislocations) or time at which successful decompression through disc or corpectomy resulted in spinal cord decompression (for anterior spinal cord compression).<br><br>**<u>Time from injury to surgery, median (range)</u>**<br><u>Early:</u> 5 (4 to 6) hours<br><u>Late:</u> 11 (8.6 to 15) hours	**<u>Inclusion:</u>**<br>• Patients aged 16-85 with initial ASIA AIS grade A-C, fracture or dislocation C3-C7, neurological level of injury between C3 and C8, cord compression confirmed by MRI, spinal canal compromise of at least 25%, and surgery within 24 hours of injury.<br><br>**<u>Exclusion:</u>**<br>• Neurological deficits before surgery, no evidence of fracture or dislocation, central cord syndrome, life threatening injuries that prevent decompression, or cognitive impairment preventing accurate neurologic assessment.<br><br>**<u>Adjusted for:</u>**<br>• Baseline AIS grade and degree of spinal canal

				A: 20% (4/20)<br>B: 5% (1/20)<br>C: 75% (15/20)				compromise.<br>• Complete/incomplete SCI.
Mattiassich (2017)<br><br>Cervical SCI<br><br>Complete/incomplete	Retrospective cohort (Poor)	**<u>Ultra-early (<5 h)</u>**<br>N = 33<br>Mean age ± SD: 47 ± 20 years<br>Male: 82%<br><br>**<u>Early (5-24 h)</u>**<br>N = 16<br>Mean age ± SD: 55 ± 18 years<br>Male: 69%	≥6 months 100% (49/49)<br><br><u>Early</u>: 100% (33/33)<br><br><u>Early</u>: 100% (16/16)	Initial neurologic examinations on admission.<br><br>**Baseline AIS grade, % (n/N)**<br><u>Ultra-early:</u><br>A: 49.0% (16/33)<br>B: 9.0% (3/33)<br>C: 24.0% (8/33)<br>D: 18.0% (6/33)<br><u>Early:</u><br>A: 25.0% (4/16)<br>B: 13.0% (2/16)<br>C: 25.0% (4/16)<br>D: 37.0% (6/16)	**Cause of trauma, % (n/N)**<br><u>Ultra-early:</u><br>Fall on the level: 13.0% (4/33)<br>Fall from heigh (≥4m): 19.0% (6/33)<br>Sports/recreation: 40.0% (13/33)<br>Motor vehicle: 28.0% (9/33)<br>Other: 3% (1/33)<br><u>Early:</u><br>Fall on the level: 44.0% (7/16)<br>Fall from height (≥4m): 19.0% (3/16)<br>Sports/recreation: 31.0% (5/16)<br>Motor vehicle: 6.0% (1/16)<br>Other: 0% (0/16)	**<u>Interventions</u>**<br>• Surgical decompression and fusion.<br><br>**<u>Co-interventions</u>**<br>NR	From incident to decompression.<br><br>**<u>Time from injury to surgery, mean ± SD</u>**<br><u>Ultra-early:</u><br>3.24 ± 1.06 hours<br><u>Early:</u> 8.60 ± 5.47 hours	**<u>Inclusion:</u>**<br>• Newly diagnosed traumatic cervical SCI, age from 15-85 years, initial neurological level between C2-T1 CT or MRI to verify compression of the spinal cord, initial AIS grade A-D, and adequate follow-up data at least 6 months after injury.<br><br>**<u>Exclusion:</u>**<br>• Non-traumatic SCI, severe craniocerebral injury (GCS <14), previously known polyneuropathy, Pre-existing major neurological deficits, dementia or severe reduction of intelligence leading to reduced capabilities of cooperation, pregnancy, surgery later than 24 h after trauma, or severe polytrauma with ISS.<br><br>**<u>Adjusted for:</u>**<br>• AIS outcome stratified by baseline AIS grade.

Aarabi (2020)<br><br>Cervical SCI<br><br>Complete/incomplete	Retrospective cohort (Fair)	<u>**Ultra-early (<12 h)**</u><br>N = 32<br>Mean age ± SD: 41.8 ± 18.4 years<br>Male: 81.3%<br><br><u>**Early (12-24 h)**</u><br>N = 25<br>Mean age ± SD: 49.4 ± 18.3 years<br>Male: 84.0%<br><br><u>**Late (>24 h)**</u><br>N = 15<br>Mean age ± SD: 49.3 ± 13.2 years<br>Male: 86.7%	≥6 months<br>100% (72/72)<br><br><u>Ultra-early</u>: 100% (32/32)<br><br><u>Early</u>: 100% (25/25)<br><br><u>Late</u>: 100% (15/15)	Initial neurologic examinations upon admission and determined stable.<br><br>**Baseline AIS grade, % (n/N)**<br><u>Ultra-early:</u><br>A: 40.6% (13/32)<br>B: 43.8% (14/32)<br>C: 15.7% (5/32)<br><u>Early:</u><br>A: 44.0% (11/25)<br>B: 28.0% (7/25)<br>C: 28.0% (7/25)<br><u>Late:</u><br>A: 20.0% (3/15)<br>B: 13.3% (2/15)<br>C: 66.7% (10/15)<br><br>**Baseline AMS, mean ± SD**<br><u>Ultra-early:</u> 18.6 ± 14.4<br><u>Early:</u> 22.0 ± 15.2<br><u>Late:</u> 24.5 ±14.2	**Cause of trauma, % (n/N)**<br><u>Ultra-early:</u><br>Fall: 43.8% (14/32)<br>Motor vehicle: 37.5% (12/32)<br>Other: 18.8% (6/32)<br><u>Early:</u><br>Fall: 56.0% (14/25)<br>Motor vehicle: 20.0% (5/25)<br>Other: 24.0% (6/25)<br><u>Late:</u><br>Fall: 46.7% (7/15)<br>Motor vehicle: 20.0% (3/15)<br>Other: 26.7% (4/15)<br><br>**Surgical technique, % (n/N)**<br><u>Ultra-early:</u><br>ACDF: 15.6% (5/32)<br>ACDF + laminectomy: 31.3% (10/32)<br>ACCF: 21.9% (7/32)<br>ACCF + laminectomy: 15.6% (5/32)<br>Laminectomy:	<u>**Interventions**</u><br>• ACDF<br>• ACDF + laminectomy<br>• ACCF<br>• ACCF + laminectomy<br>• Laminectomy<br><br><u>**Co-interventions**</u><br>NR	From trauma to decompression.<br><br><u>**Time from injury to surgery, mean ± SD**</u><br><u>Total:</u> 2.3 h ± 3.0	<u>**Inclusion:**</u><br>• Age over 16 years, GCS score ≥14, no concurrent life threatening injury or disease, imaging studies compatible with subaxial cervical spine fracture dislocations, available good quality pre- and post-operative CT and MRI studies indicating complete spinal cord decompression following surgery, and follow-up of at least 6 months after trauma and surgical management.<br><br><u>**Exclusion:**</u><br>• Being obtunded, stuperous, and non-testable, having penetrating subaxial traumatic SCI, having upper cervical SCI, a post-operative MRI indicating inadequate spinal cord decompression, non-operative management, having had a cervical CT myelogram and not an MRI as the primary study, dying or being lost to follow-up, or having poor quality imaging studies.

					15.6% (5/32)<br><u>Early:</u><br>ACDF: 8.0% (2/25)<br>ACDF + laminectomy: 44.0% (11/25)<br>ACCF: 8.0% (2/25)<br>ACCF + laminectomy: 16.0% (4/25)<br>Laminectomy: 24.0% (6/25)<br><u>Late:</u><br>ACDF: 20.0% (3/15)<br>ACDF + laminectomy: 26.7% (4/15)<br>ACCF: 0% (0/15)<br>ACCF + laminectomy: 13.3% (2/15)<br>Laminectomy: 40.0% (6/15)			**<u>Adjusted for:</u>**<br>• AIS outcome stratified by baseline AIS grade.
**Mixed SCI**								
Biglari (2016)<br><br>Cervical SCI, Thoracic SCI, Lumbar SCI	Prospective cohort (Fair)	**<u>Early (≤4 h)</u>**<br>N = 29<br>Mean age ± SD: 38.2 ± 17.8 years<br>Males: 86.2%<br><br>**<u>Late (4-24 h)</u>**<br>N = 22<br>Mean age: 50.2 ± 18.9 years<br>Males: 68.2%	6 months: 100% (51/51)<br><br><u>Early</u>: 100% (29/29)<br><br><u>Late</u>: 100% (22/22)	Initial assessment performed at admission.<br><br>**Baseline AIS grade, % (n/N)**<br><u>Early:</u><br>A: 44.8% (13/29)<br>B: 27.6% (8/29)<br>C: 24.1% (7/29)<br>D: 3.4% (1/29)<br><u>Late:</u>	**Neurological level of injury, % (n/N)**<br><u>Early:</u><br>Cervical: 41.4% (12/29)<br>Thoracic: 34.5% (10/29)<br>Lumbar: 24.1% (7/29)<br><u>Late:</u><br>Cervical: 45.5% (10/22)	**<u>Interventions</u>**<br>• Surgical decompression.<br><br>**<u>Co-interventions</u>**<br>• Ventral stabilization if necessary.	From timing of trauma to operation.<br><br>**<u>Time from injury to surgery, mean ± SD</u>**<br><u>Early:</u> 3.2 ± 0.65 hours<br><u>Late:</u> 8.2 ± 5.9 hours	**<u>Inclusion:</u>**<br>• Patients with AIS A-D, with traumatic spinal cord damage, informed consent from participants or next of kin.<br><br>**<u>Exclusion:</u>**<br>• Patients with nontraumatic acute paralysis, pregnant

				A: 50.0% (11/22)<br>B: 13.6% (3/22)<br>C: 18.2% (4/22)<br>D: 18.2% (4/22)<br><br>**Baseline GCS, mean ± SD**<br>Early: 13.3 ± 2.7<br>Late: 12.8 ± 3.8	Thoracic: 27.3% (6/22)<br>Lumbar: 27.3% (6/22)<br><br>**Cause of trauma, % (n/N):**<br>Early:<br>High-speed trauma: 41.4% 12/29)<br>Fall: 51.7% (15/29)<br>Domestic accident: 3.4% (1/29)<br>Unknown: 3.4% (1/29)<br>Late:<br>High-speed trauma: 36.4% 8/22)<br>Fall: 31.8% (7/22)<br>Domestic accident: 31.8% (7/22)<br>Unknown: 0% (0/22)			females, vertebral column cancer patients, patients operated on >24 hours after trauma, patients in a life-threatening situation with an immediate surgical contraindication, penetration injury, pre-existing neurological conditions.<br><br>**Adjusted for:**<br>• AIS outcome stratified by baseline AIS grade.

AANS = American Association of Neurological Surgeons; ACDF = Anterior cervical discectomy and fusion; ACCF = Anterior cervical corpectomy and fusion; AIDS = acquired immunodeficiency syndrome; AIS = ASIA Impairment Scale; AMS = ASIA Motor Score; ASIA = American Spinal Injury Association; CCS = central cord syndrome; CI = confidence interval; CT = computed tomography; GCS = Glasgow Coma Scale; ICU = intensive care unit; IQR = interquartile range; ISNCSCI = International Standards for Neurological Classification of Spinal Cord Injury; ISS = Injury Severity Score; MRI = magnetic resonance imaging; MVA = motor vehicle accident; NR = not reported; RHSCIR = Rick Hansen Spinal Cord Registry; OR = odds ratio; SCI = spinal cord injury; SD = standard deviation; STASCIS = Surgical Timing in Acute Spinal Cord Injury Study; tSCI = traumatic spinal cord injury.

Table D3. Detailed results for studies comparing early versus late decompression

Author (Year)<br>Injury type<br>SCI type	Neurological Outcomes	Functional, Administrative, and Other Outcomes	Complications/Adverse events
**Central cord SCI**			
Lenehan (2010)<br><br>Acute central cord injury without instability<br><br>Complete/Incomplete<br><br>From prior report	**AIS improvement from baseline to 6 months follow-up adjusted using propensity score stratification, OR (95% CI):** OR=3.39 (95% CI: 0.75 to 15.34), p=0.1131*<br><br>**AIS improvement from preoperative to 12 months follow-up adjusted using propensity score stratification OR (95% CI):** OR=2.81 (95% CI: 0.48 to 16.60), p=0.2548*<br><br>**Total motor score improvement from preoperative to 6 months follow-up adjusted using propensity score stratification, estimate (95% CI):** 7.47 (95% CI: -0.04 to 17.91), p=0.0511<br><br>**Total motor improvement from preoperative to 1 year follow-up adjusted using propensity score stratification, estimate (95% CI):** 6.31 (95% CI: 0.44 to 12.18), p=0.0359	**FIM motor sub-score improvement from discharge to 1 year adjusted using propensity score stratification, estimate (95% CI):** 6.92 (95% CI: -0.11 to 13.96), p=0.0537<br><br>**FIM total score improvement from discharge to 1 year adjusted using propensity score stratification, estimate (95% CI):** 7.79 (95% CI: 0.09 to 15.49), p=0.0474	NR
Aarabi (2021)<br><br>Acute traumatic central cord syndrome<br><br>Incomplete	**Crude AMS at ≥6 months, mean ± SD:**<br><ins>Early (≤24 h) (n=36):</ins> 91.1 ± 15.8<br><ins>Late (25-72 h) (n=38):</ins> 91.9 ± 13.4<br><ins>Very late (>72 h) (n=27):</ins> 97.5 ± 3.8<br><br>**Stepwise regression analysis of follow-up AMS, adjusted for time from injury to surgery, age, gender, etiology, baseline AMS, baseline AIS grade, number of stenosed skeletal**	NR	NR

	**segments, maximum canal compromise, point of maximum compression, number of high intensity signals on MRI, and intramedullary lesion length, estimate (95% CI):**<br>Early (≤24 h): Referent<br>Late (25-72 h): beta = -0.71 (95% CI: -5.71 to 4.28), p=0.777<br>IRR calc = 0.49 (95% CI: 0.003 to 72.24)[†]<br>Very late (>72 h): beta = 3.00 (95% CI: -2.63 to 8.62), p=0.293<br>IRR calc = 20.09 (95% CI: 0.07 to 530855280.20)[†]		
**Cervical SCI**			
Sewell (2018)<br><br>Cervical SCI<br><br>Complete/incomplete	**Crude AIS grade at 6 months, % (n/N):**<br>Early (≤24 h) (n=40):<br>A: 22.5% (9/40)<br>B: 15.0% (6/40)<br>C: 27.5% (11/40)<br>D: 32.5% (13/40)<br>E: 2.5% (1/40)<br>Late (>24 h) (n=55):<br>A: 20.0% (11/55)<br>B: 25.5.0% (14/55)<br>C: 20.0% (11/55)<br>D: 27.3% (15/55)<br>E: 5.5% (3/55)<br><br>**Crude AIS 1 grade improvement at 6 months, % (n/N):**<br>Early (≤24 h) (n=40): 32.5% (13/40)<br>Late (>24 h) (n=55): 41.8% (23/55)<br>p=0.78<br><br>**Crude AIS 2 grade improvement at 6 months, % (n/N):**<br>Early (≤24 h) (n=40): 15.0% (6/40)	**Crude length of hospital stay, mean (range):**<br>Early (≤24 h) (n=40): 14 (2 to 68) days<br>Late (>24 h) (n=55): 23 (4 to 68) days<br>P=0.013	**Crude proportion of patients with complication within first 30 days, % (n/N):**<br>Early (≤24 h) (n=40): 42.5% (17/40)<br>Late (>24 h) (n=55): 52.7% (29/55)<br><br>**Crude proportion of cardiorespiratory complications within first 30 days, % (n/N):**<br>Early (≤24 h) (n=40): 42.5% (17/40)<br>Late (>24 h) (n=55): 45.5% (25/55)<br><br>**Crude proportion of pressure sores within first 30 days, % (n/N):**<br>Early (≤24 h) (n=40): 10.0% (4/40)<br>Late (>24 h) (n=55): 12.7% (7/55)<br><br>**Crude proportion of pulmonary embolus within first 30 days, % (n/N):**<br>Early (≤24 h) (n=40): 2.5% (1/40)<br>Late (>24 h) (n=55): 3.6% (2/55)<br><br>**Crude proportion of fixation failure within first 30 days, % (n/N):**<br>Early (≤24 h) (n=40): 2.5% (1/40)

	<u>Late (>24 h) (n=55):</u> 5.5% (3/55)<br>p=0.24<br><br>**Crude AIS 3 grade improvement at 6 months, % (n/N):**<br><u>Early (≤24 h) (n=40):</u> 0% (0/40)<br><u>Late (>24 h) (n=55):</u> 1.8% (1/55)<br>p=0.24<br><br>**AIS 1 grade improvement at 6 months adjusted for incomplete SCI, OR (95% CI):**<br>OR=14.9 (95% CI: 3.1 to 72.4), p<0.01		<u>Late (>24 h) (n=55):</u> 1.8% (1/55)<br><br>**Crude proportion of wound infection within first 30 days, % (n/N):**<br><u>Early (≤24 h) (n=40):</u> 5.0% (2/40)<br><u>Late (>24 h) (n=55):</u> 5.5% (3/55)<br><br>**Crude proportion of mortality within first 30 days, % (n/N):**<br><u>Early (≤24 h) (n=40):</u> 0% (0/40)<br><u>Late (>24 h) (n=55):</u> 1.8% (1/55)
Fehlings (2012)<br><br>Cervical SCI<br><br>Complete/Incomplete<br><br>From prior report	**Crude AIS grade improvement at 6 months, % (n/N):**<br><u>Early (<24 h) (n=131):</u><br>No improvement: 42.7% (56/131)<br>1 grade improvement: 36.6% (48/131)<br>2 grade improvement: 16.8% (22/131)<br>3 grade improvement: 3.1% (4/131)<br>1 grade worsening: 0.8% (1/131)<br><u>Late (≥24 h) (n=91):</u><br>No improvement: 50.6% (46/91)<br>1 grade improvement: 40.7% (37/91)<br>2 grade improvement: 8.8% (8/91)<br>1 grade worsening: 0% (0/91)<br><br><u>Early</u><br>1+ grade improvement: 56.5% (74/131)<br><u>Late</u><br>1+ grade improvement: 49.5% (45/91)<br>OR=1.33 (95% CI: 0.78 to 2.27)<br><br><u>Early</u><br>2+ grade improvement: 19.8% (26/131)<br><u>Late</u><br>2+ grade improvement: 8.8% (8/91)<br>OR=2.57 (95% CI: 1.11 to 5.97)	NR	**Crude proportion of Cardiopulmonary complications, % (n/N):**<br><u>Early (<24 h) (n=131):</u> 17.6% (32/182)<br><u>Late (≥24 h) (n=91):</u> 26.0% (34/131)<br><br>**Crude proportion of Construct Failure, % (n/N) Requiring Surgery:**<br><u>Early (<24 h) (n=131)</u>**:** 1.6% (3/182)<br><u>Late (≥24 h) (n=91):</u> 0.8% (1/131)<br><br>**Crude proportion of Deep Wound Infection, % (n/N):**<br><u>Early (<24 h) (n=131):</u> 0% (0/182)<br><u>Late (≥24 h) (n=91):</u> 1.5% (2/131)<br><br>**Crude proportion of Neurologic Deterioration, % (n/N):**<br><u>Early (<24 h) (n=131):</u> 2.2% (4/182)<br><u>Late (≥24 h) (n=91):</u> 0.8% (1/131)<br><br>**Crude proportion of Pulmonary Embolism, % (n/N):**<br><u>Early (<24 h) (n=131):</u> 1.1% (2/182)<br><u>Late (≥24 h) (n=91):</u> 1.5% (2/131)

	**Early vs. Late after adjusting for baseline neurological status and steroid administration, OR (95% CI):**<br>Early vs. Late, 2+ grade improvement: OR=2.83 (95% CI: 1.10 to 7.28) p=0.03<br>Early vs. Late, 1+ grade improvement: OR=1.37 (95% CI: 0.80 to 2.57) p=0.31		**Crude proportion of Systemic Infection, % (n/N):**<br>Early (<24 h) (n=131): 3.3% (6/182)<br>Late (≥24 h) (n=91): 6.1% (8/131)<br><br>**Crude proportion of Wound Dehiscence, % (n/N):**<br>Early (<24 h) (n=131): 2.1% (NR)<br>Late (≥24 h) (n=91): 0.8% (NR)<br><br>**Crude proportion of Mortality; ≤30d post-injury, % (n/N):**<br>Early (<24 h) (n=131): 2.1% (NR)<br>Late (≥24 h) (n=91): 0.8% (NR)<br><br>**Crude proportion of Mortality; >30d post-injury, % (n/N):**<br>Early (<24 h) (n=131): 1.6% (3/182)<br>Late (≥24 h) (n=91): 0% (0/131)
Lee (2021)<br><br>Cervical SCI<br><br>Incomplete	**Crude AIS 1 grade improvement at 24 months, % (n/N)**<br>Early (≤24 h) (n=33): 60.6% (20/33)<br>Conservative treatment (n=21): 47.6% (10/21)<br><br>**Crude AIS 2 grade improvement at 24 months, % (n/N)**<br>Early (≤24 h) (n=33): 30.3% (10/33)<br>Conservative treatment (n=21): 9.5% (2/21)<br><br>**Multivariate linear regression of improvement in AIS grade after 24 months, adjusted for age, sex, cause of trauma, canal compression rate, spinal canal diameter, baseline AIS grade, and treatment type for early (≤24 h) vs. conservative treatment, estimate (SE) p-value**<br>Estimate = 0.543 (0.181), p=0.0044	**NR**	**Urinary tract infection**<br>Early (≤24 h) (n=33): 3.03% (1/33)<br>Conservative treatment (n=21): % (0/21)<br><br>**Pneumonia**<br>Early (≤24 h) (n=33): 0% (0/33)<br>Conservative treatment (n=21): 4.8% (1/21)<br><br>**Deep vein thrombosis**<br>Early (≤24 h) (n=33): 0% (0/33)<br>Conservative treatment (n=21): 4.8% (1/21)

	**Ordinal change in AIS grade from baseline to 24 month follow-up, stratified by baseline AIS = B, % (n/N)**<br>Early (≤24 h) (n=6):<br>B: 16.7% (1/6)<br>C: 16.7% (1/6)<br>D: 66.7% (4/6)<br>E: 0% (0/6)<br>Conservative treatment (n=5):<br>B: 60.0% (3/5)<br>C: 40.0% (2/5)<br>D: 0% (0/5)<br>E: 0% (0/5)<br><br>**Ordinal change in AIS grade from baseline to 24 month follow-up, stratified by baseline AIS = C, % (n/N)**<br>Early (≤24 h) (n=27):<br>B: 0% (0/27)<br>C: 7.4% (2/27)<br>D: 70.4% (19/27)<br>E: 22.2% (6/27)<br>Conservative treatment (n=16):<br>B: 0% (0/16)<br>C: 37.5% (6/16)<br>D: 50.0% (8/16)<br>E: 12.5% (2/16)		
Umerani (2014)<br><br>Cervical SCI<br><br>Complete/incomplete	**Crude AIS at 6 months, % (n/N):**<br>Early (≤24 h) (n=34):<br>A: 17.6% (6/34)<br>B: 11.7% (4/34)<br>C: 14.7% (5/34)<br>D: 23.5% (8/34)<br>E: 20.6% (7/34)<br>Late (>24 h) (n=64):<br>A: 21.9% (14/64)<br>B: 7.8% (5/64)	NR	**Crude mortality at 6 months, % (n/N):**<br>Early (≤24 h) (n=34): 2.9% (1/34)<br>Late (>24 h) (n=64): 6.2% (4/64)<br><br>**Crude neurological deterioration, % (n/N)**<br>Early (≤24 h) (n=34): 2.9% (1/34)<br>Late (>24 h) (n=64): 0% (0/64)

	C: 10.9% (7/64)<br>D: 37.5% (24/64)<br>E: 10.9% (7/64)<br><br>**Crude AIS ≥1 grade improvement at 6 months, % (n/N):**<br><u>Early (≤24 h) (n=34):</u> 52.9% (18/34)<br><u>Late (>24 h) (n=64):</u> 39.1% (25/64)<br>OR=3.12 (95% CI: 1.21 to 8.02)<br><br>**Crude AIS ≥2 grade improvement at 6 months, % (n/N):**<br><u>Early (≤24 h) (n=34):</u> 23.3% (7/34)<br><u>Late (>24 h) (n=64):</u> 8.7% (5/64)<br>OR=3.05 (95% CI: 0.89 to 10.51)<br><br>**Ordinal change in AIS grade from baseline to 6-month follow-up: stratified by baseline AIS = A, % (n/N):**<br><u>Early (≤24 h) (n=13):</u><br>A: 46.2% (6/13)<br>B: 15.4% (2/13)<br>C: 15.4% (2/13)<br>D: 7.7% (1/13)<br>E: 0% (0/13)<br>Dead: 7.7% (1/13)<br><u>Late (>24 h) (n=23):</u><br>A: 60.9% (14/23)<br>B: 8.7% (2/23)<br>C: 4.3% (1/23)<br>D: 4.3% (1/23)<br>E: 0% (0/23)<br>Dead: 8.7% (2/23)<br><br>**Ordinal change in AIS grade from baseline to 6-month follow-up: stratified by baseline AIS = B, % (n/N):**<br><u>Early (≤24 h) (n=4):</u>		

	A: 0% (0/4)<br>B: 25.0% (1/4)<br>C: 25.0% (1/4)<br>D: 25.0% (1/4)<br>E: 0% (0/4)<br>Dead: 0% (0/4)<br><u>Late (>24 h) (n=8):</u><br>A: 0% (0/8)<br>B: 37.5% (3/8)<br>C: 37.5% (3/8)<br>D: 12.5% (1/8)<br>E: 0% (0/8)<br>Dead: 12.5% (1/8)<br><br>**Ordinal change in AIS grade from baseline to 6-month follow-up: stratified by baseline AIS = C, % (n/N):**<br><u>Early (≤24 h) (n=10):</u><br>A: 0% (0/10)<br>B: 10.0% (1/10)<br>C: 20.0% (2/10)‡<br>D: 40.0% (4/10)<br>E: 30.0% (3/10)<br>Dead: 0% (0/10)<br><u>Late (>24 h) (n=14):</u><br>A: 0% (0/14)<br>B: 0% (0/14)<br>C: 21.4% (3/14)<br>D: 57.1% (8/14)<br>E: 14.3% (2/14)<br>Dead: 7.1% (1/14)<br><br>**Ordinal change in AIS grade from baseline to 6-month follow-up: stratified by baseline AIS = D, % (n/N):**<br><u>Early (≤24 h) (n=7):</u><br>A: 0% (0/7)<br>B: 0% (0/7)		

	C: 0% (0/7)<br>D: 28.6% (2/7)<br>E: 57.1% (4/7)<br>Dead: 0% (0/7)<br><u>Late (>24 h) (n=19):</u><br>A: 0% (0/19)<br>B: 0% (0/19)<br>C: 0% (0/19)<br>D: 73.7% (14/19)<br>E: 26.3% (5/19)<br>Dead: 0% (0/19)		
Badhiwala (2021)<br><br>Cervical SCI, thoracic SCI, Lumbosacral SCI§<br><br>Complete/Incomplete	**Change in total AMS at 12 months adjusted for baseline score, age, mechanism of injury, AIS grade, spinal level of injury, and administration of methylprednisolone, MD (95% CI):**<br><u>Early (<24 h) (n=528):</u> 23.7 (19.2 to 28.2)<br><u>Late (≥24 h) (n=1020):</u> 19.7 (15.3 to 24.0)<br>MD=4.0 (95% CI: 1.7 to 6.3) p=0.0006<br><br>**Change in light touch score at 12 months adjusted for baseline score, age, mechanism of injury, AIS grade, spinal level of injury, and administration of methylprednisolone, MD (95% CI):**<br><u>Early (<24 h) (n=528):</u> 19.0 (15.1 to 23.0)<br><u>Late (≥24 h) (n=1020):</u> 14.8 (11.2 to 18.4)<br>MD=4.3 (95% CI: 1.6 to 7.0) p=0.0021<br><br>**Change in pin prick score at 12 months adjusted for baseline score, age, mechanism of injury, AIS grade, spinal level of injury, and administration of methylprednisolone, MD**	NR	NR

	**(95% CI):**<br>Early (<24 h) (n=528): 18.3 (13.7 to 22.9)<br>Late (≥24 h) (n=1020): 14.2 (9.8 to 18.6)<br>MD=4.0 (95% CI: 1.5 to 6.6) p=0.0020<br><br>**AIS grade improvement at 12 months follow-up adjusted for baseline score, age, mechanism of injury, AIS grade, spinal level of injury, and administration of methylprednisolone, mean (95% CI), OR (95% CI):**[**]<br>Early (<24 h) (n=528):<br>A: 32.4% (28.3% to 36.4%)<br>B: 12.1% (10.3% to 13.8%)<br>C: 10.4% (8.6% to 12.1%)<br>D: 35.0% (32.4% to 37.5%)<br>E: 10.2% (8.1% to 12.3%)<br>late (≥24 h) (n=1020):<br>A: 37.9% (34.3% to 41.5%)<br>B: 11.4% (9.7% to 13.0%)<br>C: 9.7% (8.0% to 11.3%)<br>D: 33.2% (30.9% to 35.6%)<br>E: 7.8% (6.2% to 9.5%)<br>OR=1.48 (95% CI: 1.16 to 1.89), p=0.0019		
**Thoracolumbar SCI**			
Rahimi-Movaghar (2014)<br><br>Thoracolumbar tSCI<br><br>Complete/Incomplete<br><br>From prior report	**Mean AMS at 12 months, mean ± SD:**<br>Early (≤24 h) (n=16): 92 ± 12<br>Late (25-72 h) (n=19): 82 ± 16<br><br>**AIS grade at 12 months, % (n/N):**<br>Early (≤24 h) (n=16):<br>A: 31.2% (5/16)<br>B: 6.2% (1/16)<br>C: 6.2% (1/16)<br>D: 18.7% (3/16)<br>E: 31.2% (5/16)	**Mean hospital length of stay, mean ± SD:**<br>Early (≤24 h) (n=16): 7 ± 7.13 days<br>Late (25-72 h) (n=19): 9.7 ± 8.28 days<br>p>0.05	**Crude proportion of deep vein thrombosis, % (n/N):**<br>Early (≤24 h) (n=16): 6.2% (1/16)<br>Late (25-72 h) (n=19): 5.2% (1/19)<br><br>**Crude proportion of wound infection, % (n/N):**<br>Early (≤24 h) (n=16): 0% (0/16)<br>Late (25-72 h) (n=19): 5.2% (1/19)<br><br>**Crude proportion of CSF Leak, % (n/N):**

	Dead: 6.2% (1/16)<br>Late (25-72 h) (n=19):<br>A: 42.1% (8/19)<br>B: 5.2% (1/19)<br>C: 21% (4/19)<br>D: 15.7% (3/19)<br>E: 10.5% (2/19)<br>Dead: 5.2% (1/19)<br><br>**AIS 1 grade improvement at 12 months, % (n/N):**<br>Early (≤24 h) (n=16): 31.2% (5/16)<br>Late (25-72 h) (n=19): 44.0% (7/19)<br>OR calc = 0.78 (95% CI: 0.19 to 3.19), p=0.73[††]<br><br>**AIS 2 grade improvement at 12 months, % (n/N):**<br>Early (≤24 h) (n=16): 18.1% (3/16)<br>Late (25-72 h) (n=19): 5.2% (1/19)<br>OR calc = 4.15 (95% CI: 0.39 to 44.57), p=0.24[††]<br><br>**Ordinal change in AIS grade from baseline to 12-month follow-up: stratified by baseline AIS = A, % (n/N):**<br>Early (≤24 h) (n=7):<br>A: 71.4% (5/7)<br>B: 14.3% (1/7)<br>C: 0% (0/7)<br>D: 0% (0/7)<br>E: 0% (0/7)<br>Dead: 14.3 (1/7)<br>Late (25-72 h) (n=9):<br>A: 88.9% (8/9)<br>B: 11.1% (1/9)<br>C: 0% (0/9)<br>D: 0% (0/9)<br>E: 0% (0/9)		Early (≤24 h) (n=16): 0% (0/16)<br>Late (25-72 h) (n=19): 5.2% (1/19)<br><br>**Crude proportion of meningitis, % (n/N):**<br>Early (≤24 h) (n=16): 0% (0/16)<br>Late (25-72 h) (n=19): 5.2% (1/19)<br><br>**Crude proportion of decubitus ulcer, % (n/N):**<br>Early (≤24 h) (n=16): 0% (0/16)<br>Late (25-72 h) (n=19): 5.2% (1/19)<br><br>**Crude proportion of revision of surgical screws, % (n/N):**<br>Early (≤24 h) (n=16): 12.5% (2/16)<br>Late (25-72 h) (n=19): 15.7% (2/19)<br><br>**Crude proportion of bilateral rod fracture, % (n/N):**<br>Early (≤24 h) (n=16): 0% (0/16)<br>Late (25-72 h) (n=19): 5.2% (1/19)<br><br>**Crude proportion of mortality, % (n/N):**<br>Early (≤24 h) (n=16): 6.2% (1/16)<br>Late (25-72 h) (n=19): 5.2% (1/19)

	**Ordinal change in AIS grade from baseline to 12-month follow-up: stratified by baseline AIS = B, % (n/N):**<br>Early (≤24 h) (n=1):<br>A: 0% (0/1)<br>B: 0% (0/1)<br>C: 0% (0/1)<br>D: 100.0% (1/1)<br>E: 0% (0/1)<br>Late (25-72 h) (n=5):<br>A: 0% (0/5)<br>B: 0% (0/5)<br>C: 80.0% (4/5)<br>D: 20.0% (1/5)<br>E: 0% (0/5)<br><br>**Ordinal change in AIS grade from baseline to 12-month follow-up: stratified by baseline AIS = C, % (n/N):**<br>Early (≤24 h) (n=4):<br>A: 0% (0/4)<br>B: 0% (0/4)<br>C: 25.0% (1/4)<br>D: 25.0% (1/4)<br>E: 50.0% (2/4)<br>Late (25-72 h) (n=1):<br>A: 0% (0/1)<br>B: 0% (0/1)<br>C: 0% (0/1)<br>D: 0% (0/1)<br>E: 0% (0/1)<br>Dead: 100.0% (1/1)<br><br>**Ordinal change in AIS grade from baseline to 12-month follow-up: stratified by baseline AIS = D, % (n/N):**<br>Early (≤24 h) (n=3):<br>A: 0% (0/3)		

	B: 0% (0/3)<br>C: 0% (0/3)<br>D: 33.3% (1/3)<br>E: 66.7% (2/3)<br><u>Late (25-72 h) (n=5):</u><br>A: 0% (0/5)<br>B: 0% (0/5)<br>C: 0% (0/5)<br>D: 40.0% (2/5)<br>E: 60.0% (3/5)		

Qadir (2020)<br><br>Thoracolumbar SCI<br><br>Complete/incomplete	**Crude AIS ≥1 grade improvement, % (n/N):**<br>Early (<24 h) (n=144): 55.6% (80/144)<br>Intermediate (24-72 h) (n=77): 58.4% (45/77)<br>Late (>72 h) (n=96): 34.4% (33/96)<br>p=0.001<br><br>**Crude AIS ≥2 grade improvement, % (n/N):**<br>Early (<24 h) (n=144): 22.2% (32/144)<br>Intermediate (24-72 h) (n=77): 15.6% (12/77)<br>Late (>72 h) (n=96): 10.4% (10/96)<br>p=0.069<br><br>**Logistic Regression analysis of early surgery for neurologic improvement adjusted for severity of initial injury (complete vs. incomplete):**<br>p=0.004<br><br>**Ordinal change in AIS grade from baseline to 12-month follow-up: stratified by baseline AIS = A, % (n/N):**<br>Early (<24 h) (n=86):<br>A: 56.9% (49/86)<br>B: 22.1% (19/86)<br>C: 37.2% (13/86)<br>D: 3.5% (3/86)<br>E: 2.3% (2/86)<br>Intermediate (24-72 h) (n=46):<br>A: 54.3% (25/46)<br>B: 23.9% (11/46)<br>C: 15.2% (7/46)<br>D: 6.5% (3/46)<br>E: 0% (0/46)<br>Late (>72 h) (n=63):<br>A: 69.8% (44/63)<br>B: 17.5% (11/63)<br>C: 12.7% (8/63)	NR	NR

	D: 0% (0/63)<br>E: 0% (0/63)<br><br>**Ordinal change in AIS grade from baseline to 12-month follow-up: stratified by baseline AIS = B, % (n/N):**<br>Early (<24 h) (n=23):<br>A: 0% (0/23)<br>B: 21.7% (5/23)<br>C: 43.5% (10/23)<br>D: 21.7% (5/23)<br>E: 13.0% (3/23)<br>Intermediate (24-72 h) (n=6):<br>A: 0% (0/6)<br>B: 50.0% (3/6)<br>C: 50.0% (3/6)<br>D: 0% (0/6)<br>E: 0% (0/6)<br>Late (>72 h) (n=7):<br>A: 0% (0/7)<br>B: 71.4% (5/7)<br>C: 28.6% (2/7)<br>D: 0% (0/7)<br>E: 0% (0/7)<br><br>**Ordinal change in AIS grade from baseline to 12-month follow-up: stratified by baseline AIS = C, % (n/N):**<br>Early (<24 h) (n=24):<br>A: 0% (0/24)<br>B: 0% (0/24)<br>C: 17.7% (4/24)<br>D: 58.3% (14/24)<br>E: 25.0% (6/24)<br>Intermediate (24-72 h) (n=19):<br>A: 0% (0/19)<br>B: 0% (0/19)<br>C: 10.5% (2/19)		

	D: 78.9% (15/19)<br>E: 10.5% (2/19)<br>Late (>72 h) (n=15):<br>A: 0% (0/15)<br>B: 0% (0/15)<br>C: 46.7% (7/15)<br>D: 40.0% (6/15)<br>E: 13.3% (2/15)<br><br>**Ordinal change in AIS grade from baseline to 12-month follow-up: stratified by baseline AIS = D, % (n/N):**<br>Early (<24 h) (n=11):<br>A: 0% (0/11)<br>B: 0% (0/11)<br>C: 0% (0/11)<br>D: 54.5% (6/11)<br>E: 45.4% (5/11)<br>Intermediate (24-72 h) (n=6):<br>A: 0% (0/6)<br>B: 0% (0/6)<br>C: 0% (0/6)<br>D: 33.3% (2/6)<br>E: 66.7% (4/6)<br>Late (>72 h) (n=11):<br>A: 0% (0/11)<br>B: 0% (0/11)<br>C: 0% (0/11)<br>D: 63.6% (7/11)<br>E: 36.4% (4/11)		
**Thoracic SCI**			
Haghnegahdar (2020)<br><br>Thoracic SCI<br><br>Complete/incomplete	**Mean AMS at 12 months, mean ± SD:**<br>Early (<24 h) (n=37): 75.1 ± 21.2<br>Late (24-72 h) (n=36): 67.3 ± 19.2<br><br>**Mean improvement in AMS at 12 months, mean (95% CI):**	NR	**Crude proportion of deep vein thrombosis, % (n/N):**<br>Early (<24 h) (n=37): 8.1% (3/37)<br>Late (24-72 h) (n=36): 5.6% (2/36)<br><br>**Crude proportion of bilateral rod fracture, %**

Complete data of Rahimi-Movaghar 2014	Early (<24 h) (n=37): 12.8 (95% CI: 8.6 to 17.1)<br>Late (24-72 h) (n=36): 9.2 (95% CI: 5.7 to 12.7)<br><br>**AIS ≥1 grade improvement at 12 months, % (n/N):**<br>Early (<24 h) (n=37): 45.9% (17/37)<br>Late (24-72 h) (n=36): 33.3% (12/36)<br>OR=1.70 (95% CI: 0.66 to 4.39), p=0.27<br><br>**AIS ≥2 grade improvement at 12 months, % (n/N):**<br>Early (<24 h) (n=37): 24.3% (9/37)<br>Late (24-72 h) (n=36): 5.6% (2/36)<br>OR=5.46 (95% CI: 1.09 to 27.38), p=0.037<br><br>**Ordinal change in AIS grade from baseline to 12-month follow-up: stratified by baseline AIS = A, % (n/N):**<br>Early (<24 h) (n=21):<br>A: 76.2% (16/21)<br>B: 0% (0/21)<br>C: 0% (0/21)<br>D: 23.8% (5/21)<br>E: 0% (0/21)<br>Late (24-72 h) (n=20):<br>A: 95.0% (19/20)<br>B: 0% (0/20)<br>C: 5.0% (1/20)<br>D: 0% (0/20)<br>E: 0% (0/20)<br><br>**Ordinal change in AIS grade from baseline to 12-month follow-up: stratified by baseline AIS = B, % (n/N):**<br>Early (<24h) (n=5):<br>A: 0% (0/5)<br>B: 0% (0/5)<br>C: 60.0% (3/5)		**(n/N):**<br>Early (<24 h) (n=37): 0% (0/37)<br>Late (24-72 h) (n=36): 2.8% (1/36)<br><br>**Crude proportion of delayed pulled-out screw, % (n/N):**<br>Early (<24 h) (n=37): 0% (0/37)<br>Late (24-72 h) (n=36): 2.8% (1/36)[‡‡]<br><br>**Crude proportion of wound infection, % (n/N):**<br>Early (<24 h) (n=37): 0% (0/37)<br>Late (24-72 h) (n=36): 5.6% (2/36)<br><br>**Crude proportion of CSF leak, % (n/N):**<br>Early (<24 h) (n=37): 0% (0/37)<br>Late (24-72 h) (n=36): 2.8% (1/36)<br><br>**Crude proportion of meningitis, % (n/N):**<br>Early (<24 h) (n=37): 0% (0/37)<br>Late (24-72 h) (n=36): 2.8% (1/36)<br><br>**Crude proportion of decubitus ulcer, % (n/N):**<br>Early (<24 h) (n=37): 0% (0/37)<br>Late (24-72 h) (n=36): 2.8% (1/36)<br><br>**Crude proportion of complications related to methylprednisolone therapy, % (n/N):**<br>Early (<24 h) (n=37): 0% (0/37)<br>Late (24-72 h) (n=36): 0% (0/36)

	D: 20.0% (1/5)<br>E: 20.0% (1/5)<br>Late (24-72 h) (n=5):<br>A: 0% (0/5)<br>B: 0% (0/5)<br>C: 80.0% (4/5)<br>D: 20.0% (1/5)<br>E: 0% (0/5)<br><br>**Ordinal change in AIS grade from baseline to 12-month follow-up: stratified by baseline AIS = C, % (n/N):**<br>Early (<24 h) (n=4):<br>A: 0% (0/4)<br>B: 0% (0/4)<br>C: 0% (0/4)<br>D: 50.0% (2/4)<br>E: 50.0% (2/4)<br>Late (24-72 h) (n=4):<br>A: 0% (0/4)<br>B: 0% (0/4)<br>C: 0% (0/4)<br>D: 100.0% (4/4)<br>E: 0% (0/4)<br><br>**Ordinal change in AIS grade from baseline to 12-month follow-up: stratified by baseline AIS = D, % (n/N):**<br>Early (<24 h) (n=5):<br>A: 0% (0/5)<br>B: 0% (0/5)<br>C: 0% (0/5)<br>D: 80.0% (4/5)<br>E: 60.0% (3/5)<br>Late (24-72 h) (n=5):<br>A: 0% (0/7)<br>B: 0% (0/7)<br>C: 0% (0/7)		

	D: 71.4% (5/7)<br>E: 28.6% (2/7)		
**Mixed SCI**			
Bourassa-Moreau (2013)<br><br>Cervical, thoraco-lumbar SCI<br><br>Complete/Incomplete<br><br>From prior report	NR	NR	**Any complication adjusted for age, sex, Charlson Co-morbidity Index, neurological level of injury, ISS, presence of mild or moderate traumatic brain injury, and surgical invasiveness index, % (n/N):**<br>≤24 h:<br>41.1% (37/90)<br>25-72 h:<br>47.2% (109/231)<br>>72 h:<br>51.8% (57/110)<br>p=0.42<br>Logistic Regression Models[§§]<br>≤24 h vs. >72 h<br>OR=0.381; 95% CI: 0.195 to 0.743; p≤.005<br>25-72 h vs. >72 h<br>OR=0.536; 95% CI: 0.311 to 0.925; p≤0.05<br><br>**Pneumonia adjusted for age, sex, Charlson Co-morbidity Index, neurological level of injury, ISS, presence of mild or moderate traumatic brain injury, and surgical invasiveness index, % (n/N):**<br>≤24 h<br>16.7% (15/90)<br>25-72 h<br>23.8% (55/231)<br>>72 h<br>32.7% (36/110)<br>p=0.03<br>Logistic Regression Models[§§]

			≤24 h vs. >72 h<br>OR=0.275; 95% CI: 0.121 to 0.625; p≤0.005<br>25-72 h vs. >72 h<br>OR=0.473; 95% CI: 0.255 to 0.877; p≤0.05<br><br>**Pressure Ulcer adjusted for age, sex, Charlson Co-morbidity Index, neurological level of injury, ISS, presence of mild or moderate traumatic brain injury, and surgical invasiveness index, % (n/N):**<br>≤24 h<br>13.3% (12/90)<br>25-72 h<br>15.9% (37/231)<br>>72 h<br>32.7% (36/110)<br>p=0.10<br>Logistic Regression Models[§§]<br>≤24 h vs. >72 h<br>OR=0.301; 95% CI: 0.133 to 0.683; p≤0.005<br>25-72 h vs. >72 h<br>OR=0.406; 95% CI: 0.217 to 0.761; p≤0.005<br><br>**Urinary Tract Infection adjusted for age, sex, Charlson Co-morbidity Index, neurological level of injury, ISS, presence of mild or moderate traumatic brain injury, and surgical invasiveness index, % (n/N):**<br>≤24 h: 20.0% (18/90)<br>25-72 h: 23.8% (55/231)<br>>72 h: 25.5% (28/110)<br>p=0.71<br><br>**Other Complications adjusted for age, sex, Charlson Co-morbidity Index, neurological level of injury, ISS, presence of mild or moderate traumatic brain injury, and surgical invasiveness index, % (n/N):**

			<u>≤24 h:</u> 12.2% (11/90)<br><u>25-72 h:</u><br>15.9% (37/231)<br><u>>72 h:</u><br>16.5% (18/110)<br>p=0.66<br><br>**Mortality adjusted for age, sex, Charlson Co-morbidity Index, neurological level of injury, ISS, presence of mild or moderate traumatic brain injury, and surgical invasiveness index, % (n/N):**<br><u>≤24 h:</u> 3.3% (3/90)<br><u>2%-72 h:</u> 3.5% (8/231)<br><u>>72 h:</u> 0.9% (1/110)<br>p=0.39
Bourassa-Moreau (2016)<br><br>Cervical SCI, thoracolumbar SCI<br><br>Complete	**Crude AIS ≥1 grade improvement in patients with complete SCI, % (n/N):**[***]<br><u>Early (≤24 h) (n=38):</u> 34.2% (13/38)[†††]<br><u>Late (>24 h) (n=15):</u> 13.3% (2/15)[†††]<br>OR calc = 3.38 (95% CI: 0.66 to 17.30), p=0.14[‡‡]<br><br>**AIS ≥1 grade improvement adjusted for thoracolumbar SCI, % (n/N):**<br><u>Early (≤24 h) (n=20):</u> 20.0% (4/20)[†††]<br><u>Late (>24 h) (n=9):</u> 22.2% (2/9)[†††]<br>p=0.999<br><br>**AIS ≥1 grade improvement adjusted for cervical SCI, % (n/N):**<br><u>Early (≤24 h) (n=14):</u> 64.3% (9/14)[†††]<br><u>Late (>24 h) (n=6):</u> 0% (0/6)[†††]<br>p=0.008<br><br>**AIS ≥1 grade improvement adjusted for age <40 years, % (n/N):**<br><u>Early (≤24 h) (n=22):</u> 36.4% (8/22)[†††]<br><u>Late (>24 h) (n=3):</u> 33.3% (1/3)[†††]	NR	NR

	p=0.999<br><br>**AIS ≥1 grade improvement adjusted for age ≥40 years, % (n/N):**<br><u>Early (≤24 h) (n=16):</u> 31.3% (5/16)[†††]<br><u>Late (>24 h) (n=12):</u> 8.3% (1/12)[†††]<br>p=0.196		
Dvorak (2015)<br><br>Cervical, thoracic, lumbosacral SCI<br><br>Complete/Incomplete<br><br>From prior report	**ASIA Improvement Score**<br>**“Improved score” in AIS A patients adjusted for age, sex, ISS, and neurological level, % (n/N):**<br><u>Early (≤24 h):</u> NR<br><u>Late (>24 h):</u> NR<br>Beta = 0.068 (95% CI: -0.625 to 0.76); p = 0.848<br>IRR calc = 1.07 (95% CI: 0.54 to 2.14)[†]<br><br>**ASIA Improvement by 6 points in AIS B, C, and D patients adjusted for age, sex, ISS, and neurological level, % (n/N):**<br><u>Early (≤24 h):</u> NR<br><u>Late (>24 h):</u> NR<br>Beta = 6.258 (95% CI: 0.618 to 11.897); p = 0.03<br>IRR calc = 522.17 (95% CI: 1.855 to 146825.5)[†]<br><br>**AMS improvement:**<br>Author reports on AMS, but does not report AMS improvement by surgical timing.	**Length of stay (undefined):**<br><u>Early (≤24 h):</u><br>7.5 days in AIS A patients<br>Beta = -0.181 (95% CI:-0.303 to -0.059)<br>p = 0.004<br>IRR calc = 0.834 (95% CI:0.738 to 0.942)[†]<br><br>12.8 in AIS B patients<br>Beta = -0.358 (95% CI: -0.590 to -0.126)<br>p = 0.003<br>IRR calc = 0.699 (95% CI: 0.554 to 0.881)[†]<br><br><u>Late (>24 h):</u><br>Days NR	NR
Wilson (2012)<br><br>Cervical, thoracic, lumbosacral SCI<br><br>Complete/Incomplete	**Pre-op to acute-care discharge (mean 24.8 ± 29.2 days)**<br>**≥ 1 grade AIS improvement, % (n/N):**<br><u>Early (<24 h):</u> 21.2% (7/33)<br><u>Late (≥24 h)</u>: 18.4% (9/49)<br>p=0.47<br>**≥ 2 grade AIS improvement, % (n/N):**	**Mean acute care length of stay:**<br><u>Early (<24 h):</u> 24.9 days<br><u>Late (≥24 h):</u> 24.7 days<br>p=0.97<br><br>**Mean rehabilitation length of stay:**<br><u>Early (<24 h):</u> 102.9 days	NR

From prior report	Early (<24 h): 9.1% (3/33)<br>Late (≥24 h): 2.0% (1/49)<br>p=0.15<br>**AMS improvement, mean:**<br>Early (<24 h): 6.2<br>Late (≥24 h): 9.7<br>p=0.18<br><br>**Pre-op to inpatient rehabilitation discharge (mean 89.6 ± 47.4 days)**‡‡‡<br>**≥ 1 grade AIS improvement, % (n/N):**<br>Early (<24 h): 40.9% (9/22)<br>Late (≥24 h): 30.3% (10/33)<br>p=0.42<br>**≥ 2 grade AIS improvement, % (n/N):**<br>Early (<24 h): 27.2% (6/22)<br>Late (≥24 h): 3.0% (1/33)<br>p=0.01<br>**AMS improvement (mean):**<br>Early (<24 h): 19.5<br>Late (≥24 h): 15.4<br>p=0.46<br><br>**Multivariate analysis predicting change in AMS at rehabilitation discharge, adjusted for surgical timing, baseline AIS, and neurological level of injury:**<br>Early (<24 h vs. Late (≥24 h) surgery: effect estimate = 13.0; p=0.01<br>(i.e. early group ,<24 hours, experienced an additional 13 points in motor recovery as compared with late group, ≥24 hours)<br><br>**Ordinal change in AIS grade from baseline to follow-up: stratified by baseline AIS = A, % (n/N):**<br>Early (<24 h) (n=11):<br>A: 54.5% (6/11)	Late (≥24 h): 80.2 days<br>p=0.10	

	B: 0% (0/11)<br>C: 45.5% (5/11)<br>D: 0% (0/11)<br>E: 0% (0/11)<br><u>Late (≥24 h) (n=12):</u><br>A: 100.0% (12/12)<br>B: 0% (0/12)<br>C: 0% (0/12)<br>D: 0% (0/12)<br>E: 0% (0/12)<br><br>**Ordinal change in AIS grade from baseline to follow-up: stratified by baseline AIS = B, % (n/N):**<br><u>Early (<24 h) (n=4):</u><br>A: 0% (0/4)<br>B: 50.0% (2/4)<br>C: 25.0% (1/4)<br>D: 25.0% (1/4)<br>E: 0% (0/4)<br><u>Late (≥24 h) (n=2):</u><br>A: 0% (0/2)<br>B: 0% (0/2)<br>C: 50.0% (1/2)<br>D: 50.0% (1/2)<br>E: 0% (0/2)<br><br>**Ordinal change in AIS grade from baseline to follow-up: stratified by baseline AIS = C, % (n/N):**<br><u>Early (<24 h) (n=3):</u><br>A: 0% (0/3)<br>B: 0% (0/3)<br>C: 33.3% (1/3)<br>D: 66.7% (2/3)<br>E: 0% (0/3)<br><u>Late (≥24 h) (n=5):</u>		

	A: 0% (0/5)<br>B: 0% (0/5)<br>C: 0% (0/5)<br>D: 100.0% (5/5)<br>E: 0% (0/5)<br><br>**Ordinal change in AIS grade from baseline to follow-up: stratified by baseline AIS = D, % (n/N):**<br>Early (<24 h) (n=4):<br>A: 0% (0/4)<br>B: 0% (0/4)<br>C: 0% (0/4)<br>D: 100.0% (4/4)<br>E: 0% (0/4)<br>Late (≥24 h) (n=14):<br>A: 0% (0/14)<br>B: 0% (0/14)<br>C: 0% (0/14)<br>D: 78.6% (11/14)<br>E: 21.4% (3/14)		
Du (2018)<br><br>Thoracic SCI, Thoracolumbar SCI<br><br>Incomplete	**Crude AIS ≥1 grade improvement, mean + SD:**<br>Early (<24 h) (n=335): 50.7% (170/335)<br>Late (24-72 h) (n=386): 40.9% (158/386)<br>OR=1.487 (95% CI: NR), p=0.009<br><br>**AIS ≥1 grade improvement in those with AO Spine Subgroup A, % (n/N):**[§§§]<br>Early (<24 h) (n=135): 54.8% (74/135)<br>Late (24-72 h) (n=130): 53.1% (69/130)<br>OR=1.072 (95% CI: NR), p=0.777<br><br>**AIS ≥1 grade improvement in those with AO Spine Subgroup B, % (n/N):**[§§§]<br>Early (<24 h) (n=129): 48.8% (63/129)	**Unstratified PCS – SF-36, mean + SD:**<br>Early (<24 h) (n=335): 36.1 ± 9.8<br>Late (24 h to 72 h) (n=386): 35.4 ± 9.2<br>p=0.327<br><br>**PCS – SF-36 in those with AO Spine Subgroup A, mean + SD:**[§§§]<br>Early (<24 h) (n=135): 38.4 ± 11.6<br>Late (24 h to 72 h) (n=130): 38.0 ± 11.2<br>p=0.776<br><br>**PCS – SF-36 in those with AO Spine Subgroup B, mean + SD:**[§§§]<br>Early (<24 h) (n=129): 35.7 ± 9.1	**Unstratified complications, % (n/N):**<br>Early (<24 h) (n=335): 8.8% (29/335)<br>Late (24 h to 72 h) (n=386): 11.3% (43/386)<br>p=0.267<br><br>**Complications in those with AO Spine Subgroup A, % (n/N):**[§§§]<br>Early (<24 h) (n=135): 5.2% (7/135)<br>Late (24 h to 72 h) (n=130): 4.6% (6/130)<br>p=0.830<br><br>**Complications in those with AO Spine Subgroup B, % (n/N):**[§§§]<br>Early (<24 h) (n=129): 7.0% (9/129)

	Late (24-72 h) (n=153): 35.9% (55/153)<br>OR=1.701 (95% CI: NR), p=0.029<br><br>**AIS ≥1 grade improvement in those with AO Spine Subgroup C, % (n/N):**[§§§]<br>Early (<24 h) (n=71): 46.5% (33/71)<br>Late (24-72 h) (n=103): 33% (34/103)<br>OR=1.762 (95% CI: NR), p=0.007<br><br>**Crude AIS ≥2 grade improvement, % (n/N):**<br>Early (<24 h) (n=331): 11.5% (38/331)<br>Late (24-72 h) (n=380): 5% (19/380)<br>OR=2.47 (95% CI: NR), p=0.002<br><br>**AIS ≥2 grade improvement in those with AO Spine Subgroup A, % (n/N):**[§§§]<br>Early (<24 h) (n=135): 3.0% (4/135)<br>Late (24-72 h) (n=130): 0.7% (1/130)<br>OR=3.939 (95% CI: NR), p=0.189<br><br>**AIS ≥2 grade improvement in those with AO Spine Subgroup B, % (n/N):**[§§§]<br>Early (<24 h) (n=129): 17.8% (23/129)<br>Late (24-72 h) (n=153): 7.8% (12/153)<br>OR=2.550 (95% CI: NR), p=0.011<br><br>**AIS ≥2 grade improvement in those with AO Spine Subgroup C, % (n/N):**[§§§]<br>Early (<24 h) (n=71): 15.5% (11/71)<br>Late (24-72 h) (n=103): 5.8% (6/103)<br>OR=3.964 (95% CI: NR), p=0.035<br><br>**Ordinal change in AIS grade from baseline to 12-month follow-up: stratified by baseline AIS = B, % (n/N):**<br>Early (<24 h) (n=83):<br>B: 53.0% (44/83)<br>C: 22.9% (19/83)	Late (24 h to 72 h) (n=153): 33.2 ± 8.6<br>p=0.019<br><br>**PCS – SF-36 in those with AO Spine Subgroup C, mean + SD:**[§§§]<br>Early (<24 h) (n=71): 32.5 ± 7.7<br>Late (24-72 h) (n=103): 31.8 ± 7.9<br>p=0.562<br><br>**Unstratified length of hospital stay, mean ± SD:**<br>Early (<24 h) (n=335): 10.6 ± 3.3 days<br>Late (24-72 h) (n=386): 14.1 ± 4.5 days<br>P<0.0001<br><br>**Length of hospital stay in those with AO Spine Subgroup A, mean ± SD:**[§§§]<br>Early (<24 h) (n=135): 9.4 ± 3.1 days<br>Late (24-72 h) (n=130): 12.5 ± 3.5 days<br>P<0.0001<br><br>**Length of hospital stay in those with AO Spine Subgroup B, mean ± SD:**[§§§]<br>Early (<24 h) (n=129): 10.2 ± 3.4 days<br>Late (24-72 h) (n=153): 13.9 ± 3.8 days<br>P<0.0001<br><br>**Length of hospital stay in those with AO Spine Subgroup C, mean ± SD:**[§§§]<br>Early (<24 h) (n=71): 11.7 ± 3.7 days<br>Late (24-72 h) (n=103): 14.8 ± 4.5 days<br>P<0.0001	Late (24 h to 72 h) (n=153): 13.1% (20/153)<br>p=0.111<br><br>**Complications in those with AO Spine Subgroup C, % (n/N):**[§§§]<br>Early (<24 h) (n=71): 18.3 (13/71)<br>Late (24-72 h) (n=103): 16.5% (17/103)<br>p=0.757<br><br>**Thromboembolic event, % (n/N)**<br>Early (<24 h) (n=335): 4.2% (14/335)<br>Late (24 h to 72 h) (n=386): 5.4% (21/386)<br>p=0.432<br><br>**Pneumonia, % (n/N)**<br>Early (<24 h) (n=335): 1.5% (5/335)<br>Late (24 h to 72 h) (n=386): 2.6% (10/386)<br>p=0.303<br><br>**Urinary tract infection, % (n/N)**<br>Early (<24 h) (n=335): 0.9% (3/335)<br>Late (24 h to 72 h) (n=386): 1.0% (4/386)<br>p=0.848<br><br>**Decubitus ulcer, % (n/N)**<br>Early (<24 h) (n=335): 2.4% (8/335)<br>Late (24 h to 72 h) (n=386): 3.9% (15/386)<br>p=0.254<br><br>**Surgical infection, % (n/N)**<br>Early (<24 h) (n=335): 0.9% (3/335)<br>Late (24 h to 72 h) (n=386): 0.5% (2/386)<br>p=0.545<br><br>**Sepsis, % (n/N)**<br>Early (<24 h) (n=335): 0.6% (2/335)<br>Late (24 h to 72 h) (n=386): 0% (0/386)<br>p=0.128

	D: 20.5% (17/83)<br>E: 0.1% (1/83)<br><u>Late (24-72 h) (n=124):</u><br>B: 68.5% (85/124)<br>C: 24.2% (30/124)<br>D: 5.6% (7/124)<br>E: 0% (0/124)<br><br>**Ordinal change in AIS grade from baseline to 12-month follow-up: stratified by baseline AIS = C, % (n/N):**<br><u>Early (<24 h) (n=115):</u><br>B: 0% (0/115)<br>C: 40.9% (47/115)<br>D: 40.0% (46/115)<br>E: 17.4% (20/115)<br><u>Late (24-72 h) (n=96):</u><br>B: 0% (0/96)<br>C: 44.8% (43/96)<br>D: 41.7% (40/96)<br>E: 12.5% (12/96)<br><br>**Ordinal change in AIS grade from baseline to 12-month follow-up: stratified by baseline AIS = D, % (n/N):**<br><u>Early (<24 h) (n=137):</u><br>B: 0% (0/137)<br>C: 0% (0/137)<br>D: 51.1% (70/137)<br>E: 48.9% (67/137)<br><u>Late (24-72 h) (n=166):</u><br>B: 0% (0/166)<br>C: 0% (0/166)<br>D: 56.6% (94/166)<br>E: 41.6% (69/166)		

Ter Wengel (2022)<br><br>Cervical SCI, Thoracic SCI, Thoracolumbar SCI<br><br>Complete/Incomplete	**Crude AIS ≥1 grade improvement % (n/N):**<br>Early (<24 h) (n=82): 62.2% (51/82)[****]<br>Late (≥24 h) (n=14): 50.0% (7/14)[****]<br><br>**Crude AIS ≥2 grade improvement % (n/N):**<br>Early (<24 h) (n=82): 34.1% (28/82)[****]<br>Late (≥24 h) (n=14): 7.1% (1/14)[****]<br><br>**AIS ≥1 grade improvement adjusted for cervical SCI, % (n/N):**<br>Early (<24 h) (n=49): 73.5% (36/49)<br>Late (≥24 h) (n=10): 60.0% (6/10)<br>p=0.602<br><br>**AIS ≥2 grade improvement adjusted for cervical SCI, % (n/N):**<br>Early (<24 h) (n=49): 50.0% (24/49)<br>Late (≥24 h) (n=10): 10% (1/10)<br>p=0.031<br><br>**AIS ≥1 grade improvement adjusted for thoracic and thoracolumbar SCI, % (n/N):**<br>Early (<24 h) (n=33): 45.5% (15/33)<br>Late (≥24 h) (n=4): 25.0% (1/4)<br>p=0.285<br><br>**AIS ≥2 grade improvement adjusted for thoracic and thoracolumbar SCI, % (n/N):**<br>Early (<24 h) (n=33): 12.1% (4/33)<br>Late (≥24 h) (n=4): 0% (0/4)<br>p=0.031<br><br>**Multivariate analysis of ≥2 AIS grade improvement by surgical timing adjusted for level of injury, baseline AIS grade, and AO classification, OR (95% CI):**<br>Early (<24 h) (n=82): Reference<br>Late (≥24 h) (n=14): OR=0.06 (95% CI: 0.00 to	**Length of hospital stay, mean ± SD:**<br>Early (<24 h) (n=82): 25.59 ± 18.67 days<br>Late (≥24 h) (n=14): 35.15 ±25.5 days<br>p=0.108<br><br>**Length of Rehabilitation stay, mean ± SD:**<br>Early (<24 h) (n=82): 168.4 ± 93.8 days<br>Late (≥24 h) (n=14): 214.0 ± 98.5 days<br>p=0.140<br><br>**Crude >10 upper extremity motor score improvement, % (n/N):**<br>Early (<24 h) (n=82): 48.8% (40/82)[§§§]<br>Late (≥24 h) (n=14): 14.3% (2/14)[§§§]<br><br>**Crude >10 lower extremity motor score improvement, % (n/N):**<br>Early (<24 h) (n=82): 30.5% (25/82)[§§§]<br>Late (≥24 h) (n=14): 5.0% (5/14)[§§§]<br><br>**Multivariate analysis of ≥10 upper extremity motor score improvement by surgical timing adjusted for baseline AIS grade and AO classification, OR (95% CI):**<br>Early (<24 h) (n=82): Reference<br>Late (≥24 h) (n=14): OR=0.023 (95% CI: 0.02 to 1.23), p=0.130<br><br>**Multivariate analysis of ≥10 lower extremity motor score improvement by surgical timing adjusted for baseline AIS grade and AO classification, OR (95% CI):**<br>Early (<24 h) (n=82): Reference<br>Late (≥24 h) (n=14): OR=0.19 (95% CI: 0.02 to 1.13), p=0.100	**Any complication during hospital stay, % (n/N):**<br>Early (<24 h) (n=82): 59.8% (49/82)<br>Late (≥24 h) (n=14): 64.3 (9/14)<br>p=0.749

	0.47), p=0.030		

AIS = ASIA Impairment Scale; AMS = ASIA Motor Score; ASIA = American Spinal Injury Association; CI = confidence interval; FIM = Functional Independence Measure; ICU = intensive care unit; IQR = interquartile range; IRR = Incidence Rate Ratio; ISS = Injury Severity Score; NR = not reported; OR = odds ratio; PCS = Physical Component Score; SCI = spinal cord injury; SD = standard deviation; SF-36 = Short Form 36.

* Late surgery group is the reference group.

† IRR calculated from betas.

‡ AIS grade regression

§ 83% of sample is Cervical SCI.

** There is likely some overlap with Fehlings (2012), however the parent study ends follow-up at 6 months, while this individual patient data continues to 1 year. Fehlings also reports 1+ and 2+ improvement, while these pooled estimates report on any improvement.

†† OR, CIs, and p-value calculated from n's.

‡‡ Unclear if this is the same patient that experienced bilateral rod fracture.

§§ No estimates for ≤24 hours vs. 25-72 hours.

*** Considered adjusted because it only includes patients with complete SCI.

††† Estimates are back-calculated using percentage.

‡‡‡ Only 65.4% (n=55) patients had follow-up information for subgroup analysis of pre-op to inpatient rehabilitation discharge.

§§§ AO Spine Subgroup assessed according to the CT and MRI imaging.

**** Calculated by combining stratified results.

Table D4. Detailed results for studies comparing other surgical timings

Author (Year)<br>Injury type<br>SCI type	Neurological Outcomes	Functional, Administrative, and Other Outcomes	Complications/Adverse events
**Cervical SCI**			
Jug (2015)<br><br>Cervical SCI<br><br>Complete/incomplete	**Median improvement in AMS from pre-op to 6 months, median (IQR)**<br>Early (<8 h) (n=22): 38.5 (10.0 to 61.0)<br>Late (8-24 h) (n=20): 15.0 (9.9 to 34.0)<br><br>**AIS ≥2 grade improvement adjusted for baseline AIS grade and degree of spinal canal compromise, OR (95% CI):**<br>OR=11.08 (95% CI: 2.05 to 94.63), p=0.004<br><br>**Ordinal change in AIS grade from baseline to 6 month follow-up, stratified by baseline AIS = A, % (n/N):**<br>Early (<8 h) (n=13):<br>A: 46.2% (6/13)<br>B: 30.8% (4/13)<br>C: 7.7% (1/13)<br>D: 15.4% (2/13)<br>E: 0% (0/13)<br>Late (8-24 h) (n=13):<br>A: 84.6% (11/13)<br>B: 7.7% (1/13)<br>C: 0% (0/13)<br>D: 7.7% (1/13)<br>E: 0% (0/13)<br><br>**Ordinal change in AIS grade from baseline to 6 month follow-up, stratified by baseline AIS = B, % (n/N):**<br>Early (<8 h) (n=5):	**Crude length of hospital stay in days, mean ± SD:**<br>Early (<8 h) (n=22): 38.8 ± 24.0<br>Late (8-24 h) (n=20): 48.8 ± 40.3	**Crude proportion of surgical infection, % (n/N):**<br>Early (<8 h) (n=22): 0% (0/22)<br>Late (8-24 h) (n=20): 5.0% (1/20)<br><br>**Crude proportion of CSF leak, % (n/N):**<br>Early (<8 h) (n=22): 9.1% (2/22)<br>Late (8-24 h) (n=20): 0% (0/20)<br><br>**Crude proportion of cardiovascular event, % (n/N):**<br>Early (<8 h) (n=22): 0% (0/22)<br>Late (8-24 h) (n=20): 10.0% (2/20)<br><br>**Crude proportion of gastrointestinal event, % (n/N):**<br>Early (<8 h) (n=22): 4.5% (1/22)<br>Late (8-24 h) (n=20): 10.0% (2/20)

	B: 0% (0/5)<br>C: 0% (0/5)<br>D: 60.0% (3/5)<br>E: 40.0% (2/5)<br>Late (8-24 h) (n=1):<br>B: 0% (0/1)<br>C: 0% (0/1)<br>D: 100.0% (1/1)<br>E: 0% (0/1)<br><br>**Ordinal change in AIS grade from baseline to 6 month follow-up, stratified by baseline AIS = C, % (n/N):**<br>Early (<8 h) (n=4):<br>C: 0% (0/4)<br>D: 50.0% (2/4)<br>E: 50.0% (2/4)<br>Late (8-24 h) (n=6):<br>C: 0% (0/6)<br>D: 100.0% (6/6)<br>E: 0% (0/6)		
Mattiassich (2017)<br><br>Cervical SCI<br><br>Complete/incomplete	**Crude AIS grade at post-op, % (n/N):**<br>Ultra-early (<5 h) (n=33):<br>A: 42.4% (14/33)*<br>B: 12.1% (4/33)*<br>C: 15.2% (5/33)*<br>D: 30.3% (10/33)*<br>E: 0% (0/33)*<br>Early (5-24 h) (n=16):<br>A: 25.0% (4/16)*<br>B: 12.5% (2/16)*<br>C: 6.3% (1/16)*<br>D: 43.8% (7/16)*<br>E: 12.5% (2/16)*	NR	NR

	**Crude AIS at ≥6 months, % (n/N):**<br>Ultra-early (<5 h) (n=33):<br>A: 27.2% (9/33)[*]<br>B: 21.2% (7/33)[*]<br>C: 9.1% (3/33)[*]<br>D: 33.3% (11/33)[*]<br>E: 9.1% (3/33)[*]<br>Early (5-24 h) (n=16):<br>A: 6.3% (1/16)[*]<br>B: 0% (0/16)[*]<br>C: 18.8% (3/16)[*]<br>D: 50.0% (8/16)[*]<br>E: 25.0% (4/16)[*]<br><br>**Crude AIS 1 grade improvement at ≥6 months, % (n/N):**<br>Ultra-early (<5 h) (n=33): 42.4% (14/33)[*]<br>Early (5-24 h) (n=16): 31.3% (5/16)[*]<br><br>**Crude AIS 2 grade improvement at ≥6 months, % (n/N):**<br>Ultra-early (<5 h) (n=33): 6.1% (2/33)[*]<br>Early (5-24 h) (n=16): 31.3% (5/16)[*]<br><br>**Crude AIS 3 grade improvement at ≥6 months, % (n/N):**<br>Ultra-early (<5 h) (n=33): 3.0% (1/33)[*]<br>Early (5-24 h) (n=16): 6.3% (1/16)[*]<br><br>**Ordinal change in AIS grade from baseline to ≥6 months: stratified by baseline AIS = A, % (n/N):**<br>Ultra-early (<5 h) (n=16):<br>A: 56.3% (9/16)<br>B: 31.3% (5/16)<br>C: 6.3% (1/16)<br>D: 6.3% (1/16)<br>E: 0% (0/16)		

	<u>Early (5-24 h) (n=4):</u><br>A: 25.0% (1/4)<br>B: 0% (0/4)<br>C: 50.0% (2/4)<br>D: 25.0% (1/4)<br>E: 0% (0/4)<br><br>**Ordinal change in AIS grade from baseline to ≥6 months: stratified by baseline AIS = B, % (n/N):**<br><u>Ultra-early (<5 h) (n=3):</u><br>B: 66.7% (2/3)<br>C: 33.3% (1/3)<br>D: 0% (0/3)<br>E: 0% (0/3)<br><u>Early (5-24 h) (n=2):</u><br>B: 0% (0/2)<br>C: 0% (0/2)<br>D: 100% (2/2)<br>E: 0% (0/7)<br><br>**Ordinal change in AIS grade from baseline to ≥6 months: stratified by preoperative AIS = C, % (n/N):**<br><u>Ultra-early (<5 h) (n=8):</u><br>C: 12.5% (1/8)<br>D: 75.0% (6/8)<br>E: 12.5% (1/8)<br><u>Early (5-24 h) (n=4):</u><br>C: 25.0% (1/4)<br>D: 50.0% (2/4)<br>E: 25.0% (1/4)<br><br>**Ordinal change in AIS grade from baseline to ≥6 months: stratified by preoperative AIS = D, % (n/N):**<br><u>Ultra-early (<5 h) (n=6):</u><br>D: 66.7% (4/6)		

	E: 33.3% (2/6)<br>Early (5-24 h) (n=6):<br>D: 50.0% (3/6)<br>E: 50.0% (3/6)		
Aarabi (2020)<br><br>Cervical SCI<br><br>Complete/incomplete	**Crude AIS ≥1 grade improvement at ≥6 months, % (n/N):**<br>Ultra-early (<12 h) (n=32): 65.6% (21/32)<br>Early (12-24 h) (n=25): 60.0% (15/25)<br>Late (>24 h) (n=15): 80.0% (12/15)<br><br>**Multivariate regression analysis of timing of surgery on AIS conversion adjusted for age, gender, mechanism for injury, baseline AMS, baseline AIS, morphology type, surgical decompression, and Intramedullary lesion length, OR (95% CI):**<br>Ultra-early (<12 h): Referent<br>Early (12-24 h): OR=0.46 (95% CI: 0.12 to 1.75) p=0.25<br>Late (>24 h): OR=0.83 (95% CI: 0.14 to 4.88) p=0.83<br><br>**AIS grade conversion from baseline to ≥6 months: stratified by baseline AIS = A, % (n/N):**<br>Ultra-early (<12 h) (n=13):<br>A: 38.5% (5/13)<br>B: 38.5% (5/13)<br>C: 23.1% (3/13)<br>D: 0% (0/13)<br>E: 0% (0/13)<br>Early (12-24 h) (n=11):<br>A: 54.5% (6/11)<br>B: 9.1% (1/11)<br>C: 18.2% (2/11)<br>D: 18.2% (2/11)<br>E: 0% (0/11)	NR	**Neurological deteriorations, % (n/N)**<br>Ultra-early (<12 h) (n=32): 6.3% (2/32)<br>Early (12-24 h) (n=25): 4.0% (1/25)<br>Late (>24 h) (n=15): 0% (0/15)

	<u>Late (>24 h) (n=3):</u><br>A: 33.3% (1/3)<br>B: 33.3% (1/3)<br>C: 0% (0/3)<br>D: 33.3% (1/3)<br>E: 0% (0/3)<br><br>**AIS grade conversion from baseline to ≥6 months: stratified by baseline AIS = B, % (n/N):**<br><u>Ultra-early (<12 h) (n=14):</u><br>A: 14.3% (2/14)[†]<br>B: 21.4% (3/14)<br>C: 42.9% (6/14)<br>D: 21.4% (3/14)<br>E: 0% (0/14)<br><u>Early (12-24 h) (n=7):</u><br>A: 0% (0/7)<br>B: 42.9% (3/7)<br>C: 42.9% (3/7)<br>D: 6.1% (1/7)<br>E: 0% (0/7)<br><u>Late (>24 h) (n=2):</u><br>A: 0% (0/2)<br>B: 50.0% (1/2)<br>C: 0% (0/2)<br>D: 50.0% (1/2)<br>E: 0% (0/2)<br><br>**AIS grade conversion from baseline to ≥6 months: stratified by baseline AIS = C, % (n/N):**<br><u>Ultra-early (<12 h) (n=5):</u><br>A: 0% (0/5)<br>B: 0% (0/5)<br>C: 20.0% (1/5)<br>D: 60.0% (3/5)<br>E: 20.0% (1/5)		

	Early (12-24 h) (n=7):<br>A: 0% (0/7)<br>B: 14.3% (1/7)[†]<br>C: 0% (0/7)<br>D: 85.7% (6/7)<br>E: 0% (0/7)<br>Late (>24 h) (n=10):<br>A: 0% (0/10)<br>B: 0% (0/10)<br>C: 10.0% (1/10)<br>D: 90.0% (9/10)<br>E: 0% (0/10)		
**Mixed SCI**			
Biglari (2016)<br><br>Cervical SCI, Thoracic SCI, Lumbar SCI<br><br>Complete/incomplete	**Crude AIS ≥1 grade improvement, % (n/N):**<br>Ultra-early (≤4 h) (n=29): 44.8% (13/29)<br>Early (4-24 h) (n=22): 36.4% (8/22)<br><br>**Crude AIS grade at 6 months, % (n/N):**<br>Ultra-early (≤4 h) (n=29):<br>A: 41.4% (12/29)<br>B: 3.4% (1/29)<br>C: 27.6% (8/29)<br>D: 27.6% (8/29)<br>Early (4-24 h) (n=22):<br>A: 36.4% (8/22)<br>B: 9.1% (2/22)<br>C: 18.2% (4/22)<br>D: 36.4% (8/22)<br><br>**Crude logistic regression for neurologic improvement for time of surgery, OR (95% CI):**<br>OR=0.591 (95% CI: 0.173 to 2.020), p=0.402<br><br>**Ordinal change in AIS grade from baseline to 6 month follow-up, stratified by baseline AIS =**	NR	**Mortality**<br>Ultra-early (≤4 h) (n=29): 0% (0/29)<br>Early (4-24 h) (n=22): 0% (0/22)

	**A, % (n/N):**<br>Ultra-early (≤4 h) (n=13):<br>A: 92.3% (12/13)<br>B: 07% (0/13)<br>C: 7.7% (1/13)<br>Early (4-24 h) (n=11):<br>A: 72.7% (8/11)<br>B: 9.1% (1/11)<br>C: 18.2% (2/11)<br><br>**Ordinal change in AIS grade from baseline to 6 month follow-up, stratified by baseline AIS = B, % (n/N):**<br>Ultra-early (≤4 h) (n=8):<br>B: 12.5% (1/8)<br>C: 62.5% (5/8)<br>D: 25.0% (2/8)<br>Early (4-24 h) (n=3):<br>B: 33.3% (1/3)<br>C: 33.3% (1/3)<br>D: 33.3% (1/3)<br><br>**Ordinal change in AIS grade from baseline to 6 month follow-up, stratified by baseline AIS = C, % (n/N):**<br>Ultra-early (≤4 h) (n=7):<br>C: 28.6% (2/7)<br>D: 71.4 (5/7)<br>Early (4-24 h) (n=4):<br>C: 25.0% (1/4)<br>D: 75.0% (3/4)<br><br>**Ordinal change in AIS grade from baseline to 6 month follow-up, stratified by baseline AIS = D (no improvement), % (n/N):**<br>Ultra-early (≤4 h) (n=7):<br>D: 100% (1/1)<br>Early (4-24 h) (n=4):		

	D: 100% (4/4)		

AIS = ASIA Impairment Scale; AMS = ASIA Motor Score; ASIA = American Spinal Injury Association; CI = confidence interval; FIM = Functional Independence Measure; ICU = intensive care unit; IQR = interquartile range; IRR = Incidence Rate Ratio; ISS = Injury Severity Score; NR = not reported; OR = odds ratio; PCS = Physical Component Score; SCI = spinal cord injury; SD = standard deviation; SF-36 = Short Form 36.

* Estimates are back-calculated using percentage.

† AIS grade regression.

Appendix E. Excluded studies

Table E1. List of Select Excluded Studies and Rationale

	Citation	Reason for exclusion
1	Haldrup, M., et al. (2019). "Early decompressive surgery in patients with traumatic spinal cord injury improves neurological outcome." Acta Neurochir (Wien) 161(10): 2223-2228.	Does not control for baseline neurologic status or SCI severity or does not provide data on similarity of baseline status/severity
2	Nayak, B., et al. (2018). "Results of Early Versus Delayed Decompression for Traumatic Cervical Spinal Cord Injury: A Single Center Prospective Study." Indian Journal of Neurotrauma 15(1): 23-28.	Does not control for baseline neurologic status or SCI severity or does not provide data on similarity of baseline status/severity
3	Ramırez-Villaescusa, J., Lopez-Torres Hidalgo, J., Ruiz-Picazo, D., Martin-Benlloch, A., Torres-Lozano, P., and Portero-Martinez, E. (2018). The impact of urgent intervention on the neurologic recover in patients with thoracolumbar fractures. J. Spine Surg. 4, 388–396	Does not control for baseline neurologic status or SCI severity or does not provide data on similarity of baseline status/severity
4	Gaebler, C., R. Maier, et al. (1999). "Results of spinal cord decompression and thoracolumbar pedicle stabilisation in relation to the time of operation." Spinal Cord 37(1): 33-39.	Does not control for baseline neurologic status or SCI severity or does not provide data on similarity of baseline status/severity
5	Petitjean, M. E., et al. (1995). "Thoracic spinal trauma and associated injuries: should early spinal decompression be considered?" J Trauma 39(2): 368-372.	Does not control for baseline neurologic status or SCI severity or does not provide data on similarity of baseline status/severity
6	Wagner, F. C., Jr. and B. Chehrazi (1982). "Early decompression and neurological outcome in acute cervical spinal cord injuries." J Neurosurg 56(5): 699-705.	Ineligible population
7	Lubelski, D., et al. (2017). "Surgical timing for cervical and upper thoracic injuries in patients with polytrauma." J Neurosurg Spine 27(6): 633-637.	Ineligible population
8	Medress, Z., et al. (2015). "Cervical Fracture Stabilization within 72 Hours of Injury is Associated with Decreased Hospitalization Costs with Comparable Perioperative Outcomes in a Propensity Score-Matched Cohort." Cureus 7(1): e244.	Ineligible population
9	Tsuji O, Suda K, Takahata M, et al. Early surgical intervention may facilitate recovery of cervical spinal cord injury in DISH. J Orthop Surg (Hong Kong). 2019;27, 2309499019834783.	Ineligible intervention
10	Chen Qi, Li Feng, Fang Zhong, et al. Timing of surgical decompression for acute traumatic cervical spinal cord injury: a multicenter study. Neurosurg Q. 2012;22:61e68	Ineligible intervention
11	Nasi, D., et al. (2019). "Ultra-early surgery in complete cervical spinal cord injury improves neurological recovery: A single-center retrospective study." Surgical Neurology International 10: 1-5.	Ineligible intervention
12	Dobran, M., Iacoangeli, M., Nocchi, N., Di Rienzo, A., di Somma, L.G., Nasi, D., Colasanti, R., Al-Fay, M., and Scerrati, M. (2015). Surgical treatment of cervical spine trauma: Our experience and results. Asian J. Neurosurg. 10, 207–211.	Ineligible intervention
13	McCarthy, M. J., S. Gatehouse, et al. (2011). "The influence of the energy of trauma, the timing	Ineligible intervention

	of decompression, and the impact of grade of SCI on outcome." Evid Based Spine Care J 2(2): 11-17	
14	Chikuda H et. al. (2021). Effect of Early vs Delayed Surgical Treatment on Motor Recovery in Incomplete Cervical Spinal Cord Injury With Preexisting Cervical Stenosis: A Randomized Clinical Trial. JAMA Netw Open. Nov 1;4(11):e2133604. doi: 10.1001/jamanetworkopen.2021.33604. PMID: 34751757.	Ineligible intervention
15	Balas, M., et al. (2021). "The Reality of Accomplishing Surgery Within 24 hours for Complete Cervical Spinal Cord Injury: Clinical Practices and Safety." J Neurotrauma.	Ineligible intervention
16	Godzik, J., et al. (2019). "Early surgical intervention among patients with acute central cord syndrome is not associated with higher mortality and morbidity." J Spine Surg 5(4): 466-474.	Ineligible intervention
17	Burke, J. F., et al. (2019). "Ultra-Early (<12 Hours) Surgery Correlates With Higher Rate of American Spinal Injury Association Impairment Scale Conversion After Cervical Spinal Cord Injury." Neurosurgery 85(2): 199-203.	Ineligible intervention
18	Kim, M., et al. (2018). "Early (≤48 Hours) versus Late (>48 Hours) Surgery in Spinal Cord Injury: Treatment Outcomes and Risk Factors for Spinal Cord Injury." World Neurosurg 118: e513-e525.	Ineligible intervention
19	Gupta, D. K., et al. (2015). "Early versus delayed decompression in acute subaxial cervical spinal cord injury: A prospective outcome study at a Level I trauma center from India." Asian J Neurosurg 10(3): 158-165.	Ineligible intervention
20	Du, J. P., et al. (2019). "Early versus delayed decompression for traumatic cervical spinal cord injury: application of the AOSpine subaxial cervical spinal injury classification system to guide surgical timing." European spine journal 28(8): 1855-1863.	Ineligible intervention
21	Kim, E. J., et al. (2018). "Timing of Operative Intervention in Traumatic Spine Injuries Without Neurological Deficit." Neurosurgery 83(5): 1015-1022.	Ineligible intervention
22	Liu, Y., et al. (2015). "Timing of surgical decompression for traumatic cervical spinal cord injury." Int Orthop 39(12): 2457-2463.	Ineligible intervention
23	Mahon, J., et al. (2020). "Timing of surgical fixation in traumatic spinal fractures." Bone Joint J 102-B(5): 627-631.	Ineligible intervention
24	Mayol, M., et al. (2019). "Time of Surgery in the Outcome of Cervical Spinal Cord Injury: the University of Puerto Rico Experience." P R Health Sci J 38(2): 109-112.	Ineligible intervention
25	Zheng, C., et al. (2020). "Early Surgical Decompression Ameliorates Dysfunction of Spinal Motor Neuron in Patients with Acute Traumatic Central Cord Syndrome: An Ambispective Cohort Analysis." Spine 45(14): E829-E838.	Ineligible intervention
26	Tian, C., Lv, Y., Li, S., Wang, D.D., Bai, Y., Zhou, F., and Ma, Q.B. (2020). Factors related to improved American Spinal Injury Association grade of acute traumatic spinal cord injury. World J. Clin. Cases 8, 4807–4815.	Ineligible intervention
27	Cengiz, S. L., E. Kalkan, et al. (2008). "Timing of thoracolomber spine stabilization in trauma patients; impact on neurological outcome and clinical course. A real prospective (rct) randomized controlled study." Arch Orthop Trauma Surg 128(9): 959-966	Ineligible intervention

28	Sapkas, G. S. and S. A. Papadakis (2007). "Neurological outcome following early versus delayed lower cervical spine surgery." J Orthop Surg 15(2): 183-186.	Ineligible intervention
29	Vaccaro, A. R., R. J. Daugherty, et al. (1997). "Neurologic outcome of early versus late surgery for cervical spinal cord injury." Spine (Phila Pa 1976) 22(22): 2609-2613.	Ineligible intervention
30	Yamazaki, T., K. Yanaka, et al. (2005). "Traumatic central cord syndrome: analysis of factors affecting the outcome." Surg Neurol 63(2): 95-99; discussion 99-100.	Ineligible intervention
31	Prasad, V. S., J. V. Vidyasagar, et al. (1995). "Early surgery for thoracolumbar spinal cord injury: initial experience from a developing spinal cord injury centre in India." Paraplegia 33(6): 350-353.	Ineligible intervention
32	Tator, C. H., E. G. Duncan, et al. (1987). "Comparison of surgical and conservative management in 208 patients with acute spinal cord injury." Can J Neurol Sci 14(1): 60-69.	Ineligible intervention
33	Tator, C. H., et al. (1987). "Comparison of surgical and conservative management in 208 patients with acute spinal cord injury." Can J Neurol Sci 14(1): 60-69.	Ineligible intervention
34	Kiwerski, J. E. (1993). "Early anterior decompression and fusion for crush fractures of cervical vertebrae." Int Orthop 17(3): 166-168.	Ineligible intervention
35	Chen, T. Y., et al. (1997). "Efficacy of surgical treatment in traumatic central cord syndrome." Surgical Neurology 48(5): 435-441.	Ineligible intervention
36	Jug, M., et al. (2020). "Window of opportunity for surgical decompression in patients with acute traumatic cervical spinal cord injury." Journal of neurosurgery: spine 32(5): 633-641.	Ineligible study design for Key Question, e.g., case series, modeling (e.g., prediction models, thresholds/ROC, etc.)
37	Facchinello, Y., et al. (2018). "The use of classification tree analysis to assess the influence of surgical timing on neurological recovery following severe cervical traumatic spinal cord injury." Spinal Cord 56(7): 687-694.	Ineligible study design for Key Question, e.g., case series, modeling (e.g., prediction models, thresholds/ROC, etc.)
38	Grassner, L., et al. (2016). "Early decompression (< 8 h) after traumatic cervical spinal cord injury improves functional outcome as assessed by spinal cord independence measure after one year." Journal of neurotrauma 33(18): 1658-1666.	Ineligible study design for Key Question, e.g., case series, modeling (e.g., prediction models, thresholds/ROC, etc.)
39	Wutte, C., et al. (2020). "Early Decompression (<8 Hours) Improves Functional Bladder Outcome and Mobility After Traumatic Thoracic Spinal Cord Injury." World Neurosurg 134: e847-e854.	Ineligible study design for Key Question, e.g., case series, modeling (e.g., prediction models, thresholds/ROC, etc.)
40	Wutte, C., et al. (2019). "Earlier Decompression (< 8 Hours) Results in Better Neurological and Functional Outcome after Traumatic Thoracolumbar Spinal Cord Injury." Journal of neurotrauma 36(12): 2020-2027.	Ineligible study design for Key Question, e.g., case series, modeling (e.g., prediction models, thresholds/ROC, etc.)
41	Goulet, J., Richard-Denis, A., and Mac-Thiong, J.M. (2020). The use of classification and regression tree analysis to identify the optimal surgical timing for improving neurological outcomes following motorcomplete	Ineligible study design for Key Question, e.g., case series, modeling (e.g., prediction models, thresholds/ROC, etc.)
42	Parent, S., et al. (2012). "Non-neurological complication rate following surgical treatment of vertebral fracture with spinal cord injury: Does surgical timing matter?" Spine Journal 12(9): 127S.	Not a study (trial protocol, letter, editorial, non-systematic review article, abstract only)
43	Chikuda, H., et al. (2013). "Optimal treatment for spinal cord injury associated with cervical	Not a study (trial protocol, letter, editorial, non-

	canal stenosis (OSCIS): a study protocol for a randomized controlled trial comparing early versus delayed surgery." Trials 14: 245.	systematic review article, abstract only)
44	Bortz, C., et al. (2019). "238. Same-day surgical intervention dramatically minimizes complication occurrence and optimizes perioperative outcomes for central cord syndrome." Spine journal 19(9): S116-S117.	Not a study (trial protocol, letter, editorial, non-systematic review article, abstract only)
45	Bortz, C., et al. (2019). "Same day surgical intervention dramatically minimizes complication occurrence and optimizes perioperative outcomes for central cord syndrome." Clinical Neurosurgery 66: 57.	Not a study (trial protocol, letter, editorial, non-systematic review article, abstract only)
46	Aarabi, B., et al. (2020). "Response to Burke et al.: Efficacy of Ultra-Early (<12 h), Early (12-24 h), and Late (>24-138.5 h) Surgery with Magnetic Resonance Imaging-Confirmed Decompression in American Spinal Injury Association Impairment Scale Grades A, B, and C Cervical Spinal Cord Injury (DOI: 10.1089/neu.2020.7034)." Journal of neurotrauma 37(21): 2343-2344.	Not a study (trial protocol, letter, editorial, non-systematic review article, abstract only)
47	Badhiwala, J. H., et al. (2018). "The impact of time to surgical decompression for acute traumatic central cord syndrome." Journal of neurotrauma 35(16): A51.	Not a study (trial protocol, letter, editorial, non-systematic review article, abstract only)
48	Badhiwala, J. H., et al. (2019). "Early versus late surgical decompression for central cord syndrome: A propensity score-matched analysis." Clinical Neurosurgery 66: 112-113.	Not a study (trial protocol, letter, editorial, non-systematic review article, abstract only)
49	Burke, J. F., et al. (2016). "Ultra-Early (<12 Hours) decompression improves recovery after spinal cord injury compared to early (12-24 hours) decompression." Clinical Neurosurgery 63: 172.	Not a study (trial protocol, letter, editorial, non-systematic review article, abstract only)
50	Haghnegahdar, A., et al. (2018). "Early versus late surgery for traumatic spinal cord injury in the T1-L1 Area-second results of an RCT at one-year follow-up." Journal of neurotrauma 35(16): A43- .	Not a study (trial protocol, letter, editorial, non-systematic review article, abstract only)
51	Olexa, J. R., et al. (2019). "Magnetic resonance imaging evidence of therapeutic efficacy of timing of decompression in American spinal injury association impairment scale grades a to c cervical spinal cord injury patients." Clinical Neurosurgery 66: 112.	Not a study (trial protocol, letter, editorial, non-systematic review article, abstract only)
52	Wutte, C., et al. (2019). "Earlier decompression (< 8 hours) improves the neurological and functional outcome after traumatic thoracolumbar spinal cord injury." European spine journal 28: 2698-2699.	Not a study (trial protocol, letter, editorial, non-systematic review article, abstract only)
53	Wutte, C., et al. (2019). "Early decompression (< 8 hours) improves the functional bladder outcome and mobility after traumatic thoracic spinal cord injury." European spine journal 28: 2698.	Not a study (trial protocol, letter, editorial, non-systematic review article, abstract only)
54	Badhiwala, J. H., et al. (2018). "The safety and efficacy of early surgery for traumatic central cord syndrome." Clinical Neurosurgery 65: 105.	Not a study (trial protocol, letter, editorial, non-systematic review article, abstract only)
55	Barzideh, E., et al. (2017). "Early versus late surgery for traumatic spinal cord injury in the thoracic or thoracolumbar area: secondary results of a randomized controlled trial at one-year follow-up." Global spine journal 7(2): 122S- 123S.	Not a study (trial protocol, letter, editorial, non-systematic review article, abstract only)
56	Burke, J. F., et al. (2020). "Effect of ultra-early (<12 hours) surgery on recovery after cervical spinal cord injury: A track-sci study." Journal of neurosurgery 132(4): 20.	Not a study (trial protocol, letter, editorial, non-systematic review article, abstract only)

57	Fehlings, M. G., et al. (2021). "Early (<24 hrs) versus late (≥24 hrs) surgical decompression for central cord syndrome: A propensity score-matched analysis." Journal of neurosurgery 135(2): 69.	Not a study (trial protocol, letter, editorial, non-systematic review article, abstract only)
58	Hosman, A. J. F., et al. (2016). "Interim findings from the sci-poem study: Logistic barriers to early surgical decompression following spinal cord injury." European spine journal 25: S322.	Not a study (trial protocol, letter, editorial, non-systematic review article, abstract only)
59	Jefferson, W., et al. (2018). "Impact of injury severity on the relationship between time to surgical decompression and neurological recovery and functional outcomes following traumatic cervical spinal cord injury." Global spine journal 8(1): 76S-77S.	Not a study (trial protocol, letter, editorial, non-systematic review article, abstract only)
60	Jug, M., et al. (2018). "The window of opportunity for surgical decompression in patients with acute traumatic cervical spinal cord injury." European spine journal 27: S610-S611.	Not a study (trial protocol, letter, editorial, non-systematic review article, abstract only)
61	Kim, D. H., et al. (2017). "Efficacy of surgical decompression within the first 8 hours versus 8 to 24 hours after acute traumatic spinal cord injury." Global spine journal 7(2): 341S.	Not a study (trial protocol, letter, editorial, non-systematic review article, abstract only)
62	Qutteineh, B., et al. (2018). "Early decompression for spinal cord injury: The faster the better." Global spine journal 8(1): 128S-129S.	Not a study (trial protocol, letter, editorial, non-systematic review article, abstract only)
63	Wilson, J., et al. (2017). "Natural history, mortality, complications and impact of early surgical decompression in thoracic spinal cord injury: A multicenter prospective study from the North American clinical trials network and aospine spinal cord injury knowledge forum." Global spine journal 7(2): 121S.	Not a study (trial protocol, letter, editorial, non-systematic review article, abstract only)
64	Yong, F., et al. (2018). "Early versus delayed decompression for traumatic cervical spinal cord injury: Application of the aospine subaxial cervical spinal injury classification system to guide surgical timing." European spine journal 27: S611.	Not a study (trial protocol, letter, editorial, non-systematic review article, abstract only)
65	Hao, D., et al. (2017). "Optimal timing for traumatic cervical spinal cord injury with surgical decompression: 10 years cases reviewed." European spine journal 26(2): S333.	Not a study (trial protocol, letter, editorial, non-systematic review article, abstract only)
66	Samuel AM, Bohl DD, Basques BA, et al. Analysis of Delays to Surgery for Cervical Spinal Cord Injuries. Spine (Phila Pa 1976) 2015;40:992-1000.	Not a study (trial protocol, letter, editorial, non-systematic review article, abstract only)
67	Ling, S. Y., et al. (2016). "Early surgical intervention for acute incomplete cervical spinal cord injury: An analysis of 387 cases." Academic Journal of Second Military Medical University 37(6): 761-766.	Not English language but possibly relevant
68	Ehsaei M, Samini F, TaghaviM. Comprative evaluation of outcomes for early and late decompressive surgery in patients with traumatic injuries of the spinal cord, in thoracic and throvacolumbar regions. Med J Mashhad Uni Med Sci. 2014;57(1):436-42.	Not English language but possibly relevant
69	Sharma, M., et al. (2019). "Impact of Surgical Timing and Approaches to Health Care Utilization in Patients Undergoing Surgery for Acute Traumatic Cervical Spinal Cord Injury." Cureus 11(11): e6166.	Does not control for baseline neurologic status or SCI severity or does not provide data on similarity of baseline status/severity. Ineligible intervention.
70	Bortz, C., et al. (2021). "Same Day Surgical Intervention Dramatically Minimizes Complication Occurrence and Optimizes Perioperative Outcomes for Central Cord Syndrome." Clin Spine Surg.	Does not control for baseline neurologic status or SCI severity or does not provide data on similarity of baseline status/severity. Ineligible intervention.
71	Chen, L., H. Yang, et al. (2009). "Effectiveness of surgical treatment for traumatic central cord syndrome." J Neurosurg Spine 10(1): 3-8	Does not control for baseline neurologic status or SCI severity or does not provide data on similarity of baseline status/severity. Ineligible intervention.

72	Dakson, A., et al. (2017). "Optimization of the mean arterial pressure and timing of surgical decompression in traumatic spinal cord injury: a retrospective study." Spinal Cord 55(11): 1033-1038.	Does not control for baseline neurologic status or SCI severity or does not provide data on similarity of baseline status/severity. Ineligible intervention. Ineligible study design for Key Question, e.g., case series, modeling (e.g., prediction models, thresholds/ROC, etc.)
73	Aarabi, B., Sansur, C.A., Ibrahimi, D.M., Simard, J.M., Hersh, D.S., Le, E., Diaz, C., Massetti, J., and Akhtar-Danesh, N. (2017). Intramedullary lesion length on postoperative magnetic resonance imaging is a strong predictor of ASIA Impairment Scale grade conversion following decompressive surgery in cervical spinal cord injury. Neurosurgery 8, 610–620.	Does not control for baseline neurologic status or SCI severity or does not provide data on similarity of baseline status/severity. Ineligible intervention. Ineligible study design for Key Question, e.g., case series, modeling (e.g., prediction models, thresholds/ROC, etc.)
74	Ruddell, J. H., et al. (2021). "Timing of Surgery for Thoracolumbar Spine Trauma: Patients With Neurological Injury." Clin Spine Surg 34(4): E229-e236.	Does not control for baseline neurologic status or SCI severity or does not provide data on similarity of baseline status/severity. Ineligible population. Ineligible intervention
75	Inoue, T., et al. (2017). "Efficacy of Early Surgery for Neurological Improvement in Spinal Cord Injury without Radiographic Evidence of Trauma in the Elderly." World neurosurgery 105: 790-795.	Ineligible population. Ineligible intervention
76	Pointillart V, Petitjean ME, Wiart L, et al. Pharmacological therapy of spinal cord injury during the acute phase. Spinal cord 2000;38:71- 6.	Does not control for baseline neurologic status or SCI severity or does not provide data on similarity of baseline status/severity. Ineligible intervention
77	Schroeder GD, Kepler CK, Hjelm N, Vaccaro AR, Weinstein MS. The effect of vertebral fracture on the early neurologic recovery in patients with central cord syndrome. Eur Spine J. 2015;24(5):985-989.	Ineligible outcomes. Does not control for baseline neurologic status or SCI severity or does not provide data on similarity of baseline status/severity. Ineligible intervention
78	Tanaka C, Tagami T, Kaneko J, et al. Early versus late surgery after cervical spinal cord injury: a Japanese nationwide trauma database study. J Orthop Surg Res 2019;14:302.	Does not control for baseline neurologic status or SCI severity or does not provide data on similarity of baseline status/severity.

Appendix F. Contemporary systematic reviews reporting on early vs. late timing of surgery

Table F1. Rating overall Confidence in the Results of the Review (Dettori 2020).[7]

High: No or 1 noncritical weakness	The systematic review provides an accurate and comprehensive summary of the results of the available studies that address the question of interest.
Moderate: More than 1 noncritical weakness*	The systematic review has more than 1 weakness but no critical flaws. It may provide an accurate summary of the results of the available studies that were included in the review.
Low: One critical flaw with or without noncritical weaknesses	The review has a critical flaw and may not provide an accurate and comprehensive summary of the available studies that address the question of interest.
Critically low: More than 1 critical flaw with or without noncritical weaknesses	The review has more than 1 critical flaw and should not be relied on to provide an accurate and comprehensive summary of the available studies.

* Multiple noncritical weaknesses may diminish confidence in the review, and it may be appropriate to move the overall appraisal down from moderate to low confidence.

Table F2. Summary table of contemporary systematic reviews reporting on early vs. late timing of surgery

SR,<br>Search dates,<br>Search databases	Purpose	Interventions	Primary Outcomes	Evidence Base	AMSTAR-2 rating	Primary Conclusions
**Traumatic central cord syndrome**						
Andersen 2015<br><br>Database inception through March 2015<br><br>PubMed, Cochrane Library, ClinicalTrials.gov, Web of Science, PubMed Health	Investigate neurological outcomes, length of stay, and complications by timing of surgery in TCCS patients	Surgery <24 hours vs. >24 hours after injury	<u>Efficacy</u><br>Difference in ASIA AIS, motor score, JOA,<br><br><u>Function</u><br>Difference in FIM, Frankel Grade<br><br><u>Administrative</u><br>Difference in	9 cohort studies (1596 patients)<br><br>Not all included studies adjusted for baseline characteristics<br><br>RoB: LoE<br><br>No pooled measures	Critically low	<u>Efficacy</u><br>- <24 hours vs. >24 hours: difference in post-operative ASIA motor score at 6 months and 1 year (1 prospective cohort; weak evidence)<br>- <24 hours vs. >24 hours: difference in ASIA motor score (3 retrospective cohorts; insufficient evidence)<br>- <2 weeks vs. >2 weeks: difference in post-operative JOA score and recovery rate (1 retrospective cohort; moderate evidence)

SR,<br>Search dates,<br>Search databases	Purpose	Interventions	Primary Outcomes	Evidence Base	AMSTAR-2 rating	Primary Conclusions
			LoHS, ICU stays<br><br><u>Safety</u><br>Difference in complications, mortality			<u>Function</u><br>- <24 hours vs. >24 hours: difference in Frankel Grade (1 retrospective cohort; insufficient evidence)<br>- <24 hours vs. >24 hours: improvement in FIM score between discharge and 1 year (1 prospective cohort, weak evidence)<br><br><u>Administrative</u><br>- <24 hours vs. >24 hours: difference in LoHS or ICU stays (2 retrospective cohorts, insufficient evidence)<br><br><u>Safety</u><br>- <24 hours vs. >24 hours: difference in mortality or complication rates (1 retrospective cohort, insufficient evidence)
**Incomplete spinal cord injury**						
Liu 2016<br><br>Database inception through March 2015<br><br>PubMed, MEDLINE, Cochrane Library, Google Scholar	Investigate neurological outcomes by timing of surgery	Surgery <24 hours vs. >24 hours after injury	<u>Efficacy</u><br>Difference in total motor score, difference in neurological improvement*<br><br><u>Function</u><br>NR<br><br><u>Administrative</u><br>Difference in LoHS, ICU stay	7 cohort studies, 2 RCTs (734 patients)<br><br>Studies adjusted for baseline characteristics<br><br>RoB: Newcastle-Ottowa Quality Assessment Scale	Low	<u>Efficacy</u><br>- <24 hours vs. >24 hours: difference in total motor score (1 retrospective cohort, 1 prospective cohort, 2 RCTs; MD = 3.30, 95% CI = 0.82 to 5.79)<br>- <24 hours vs. 24 hours: difference in neurologic improvement (4 prospective cohorts, 1 retrospective cohort, 2 RCTs; OR = 1.66, 95% CI = 1.19 to 2.31)<br><br><u>Function</u><br>NR

SR,<br>Search dates,<br>Search databases	Purpose	Interventions	Primary Outcomes	Evidence Base	AMSTAR-2 rating	Primary Conclusions
			Safety<br>Difference in complications, mortality			Administrative<br>- <24 hours vs. >24 hours: difference in LoHS (1 retrospective cohort, 1 prospective cohort, 2 RCTs; MD = - 4.76, 95% CI = -9.19 to -0.32)<br>- <24 hours vs. >24 hours: 2/3 (66%) studies show shorter ICU stay (2 retrospective cohorts, 1 RCT; meta-analysis not carried out due to missing SDs)<br><br>Safety<br>- <24 hours vs. >24 hours: difference in complication rates (2 retrospective cohorts, 2 prospective cohorts, 2 RCTs; OR = 0.61, 95% CI = 0.40 to 0.91)<br>- <24 hours vs. >24 hours: difference in mortality rates (3 retrospective cohorts, 3 prospective cohorts, 2 RCTs; OR = 1.39, 95% CI = 0.51 to 3.75)
**Mixed (complete and incomplete) spinal cord injury**						
Hsieh 2021<br><br>Database inception through December 2020<br><br>PubMed, Embase	Investigate neurological outcomes by timing of surgery	Early surgery vs. late surgery (<12 hours vs. >12 hours or <24 hours vs. >24 hours)	Efficacy<br>Improvement in ASIA AIS ≥1 grade<br><br>Function<br>NR<br><br>Administrative<br>NR<br><br>Safety<br>NR	24 cohort studies, 1 RCT, 1 quasi-RCT (3574 patients)<br><br>Not all studies adjusted for baseline characteristics<br><br>RoB: Newcastle-Ottowa Quality	Low	Efficacy<br>- Surgery at earliest time point in study: unadjusted neurological recovery ≥1 AISA AIS grade (10 prospective cohorts, 14 retrospective cohorts, 1 RCT, 1 quasi-RCT; OR = 1.85, 95% CI = 1.41 to 2.41)<br>- Surgery at earliest time point in study: unadjusted neurological recovery ≥2 AISA AIS grade (7 prospective cohorts, 9 retrospective cohorts, 1 RCT, 1 quasi-RCT; OR = 2.22, 95% CI = 1.31 to 3.75)<br>- <12 hours vs. >12 hours: neurological

SR,<br>Search dates,<br>Search databases	Purpose	Interventions	Primary Outcomes	Evidence Base	AMSTAR-2 rating	Primary Conclusions
				Assessment Scale		recovery ≥1 AISA AIS grade (5 retrospective cohorts; OR = 3.33, 95% CI = 0.76 to 14.57)<br>- <24 hours vs. >24 hours: neurological recovery ≥1 AISA AIS grade (2 prospective cohorts, 6 retrospective cohorts, 1 RCT; OR = 1.50, 95% CI = 1.34 to 1.66)<br><br><u>Function</u><br>NR<br><br><u>Administrative</u><br>NR<br><br><u>Safety</u><br>NR
Ter Wengel 2019a<br><br>Database inception through November 2017<br><br>PubMed, Embase	Investigate neurological outcomes by timing of surgery	Surgery <24 hours vs. >24 hours after injury	<u>Efficacy</u><br>Improvement of ASIA AIS ≥1 grade<br><br><u>Function</u><br>NR<br><br><u>Administrative</u><br>NR<br><br><u>Safety</u><br>NR	15 cohort studies (1126 patients<br><br>Not all studies adjusted for baseline characteristics<br><br>RoB: Newcastle-Ottowa Quality Assessment Scale	Critically low	<u>Efficacy</u><br>- <24 hours vs. >24 hours: unadjusted neurological improvement of ≥2 ASIA AIS grades (14 cohorts, types NR; OR = 1.1, 95% CI = 0.8 to 1.6)<br>- <24 hours vs. >24 hours: neurological improvement of ≥2 ASIA AIS grades in severe incomplete (B, C) (8 cohorts, types NR; OR = 2.0, 95% CI = 0.9 to 6.2)<br>- <24 hours vs. >24 hours: neurological improvement of ≥2 ASIA AIS grades in complete (A) SCI (13 cohorts, types NR; OR = 2.6, 95% CI = 1.4 to 5.1)<br>- <24 hours vs. >24 hours: neurological improvement of ≥2 ASIA AIS grades in incomplete (B, C, D) SCI (10 cohorts, types NR; OR = 0.9, 95% CI = 0.4 to 1.9)

SR, Search dates, Search databases	Purpose	Interventions	Primary Outcomes	Evidence Base	AMSTAR-2 rating	Primary Conclusions
						- <8 hours vs. >8 hours: proportion of patients with complete SCI with neurological improvement: 20/69 (29%) (studies NR; pooled estimates NR)<br>- <8 hours vs. >8 hours: proportion of patients with incomplete SCI with neurological improvement: 9/50 (18%) (studies NR; pooled estimates NR)<br><br><u>Function</u><br>NR<br><br><u>Administrative</u><br>NR<br><br><u>Safety</u><br>NR
Ter Wengel 2019b<br><br>Database inception through July 2018<br><br>PubMed, Embase	Investigate neurological outcomes by timing of surgery in patients with thoracic and/or thoracolumbar SCI	Surgery <24 hours vs. >24 hours after injury	<u>Efficacy</u><br>Improvement of ASIA AIS ≥1 grade or Grade E at end of follow-up<br><br><u>Function</u><br>NR<br><br><u>Administrative</u><br>NR<br><br><u>Safety</u><br>NR	12 cohort studies, 1 RCT, 1 quasi-RCT (1075 patients)<br><br>Not all studies adjusted for baseline characteristics<br><br>RoB: Newcastle-Ottawa Quality Assessment Scale	Critically low	<u>Efficacy</u><br>- <24 hours vs. >24 hours: neurological improvement of ≥1 ASIA AIS grades (11 cohorts, types NR; OR = 2.2, 95% CI = 0.6 to 14.0)<br>- <24 hours vs. >24 hours: neurological improvement of ≥2 ASIA AIS grades (11 cohorts, types NR; OR = 1.9, 95% CI = 0.6 to 7.3)<br>- <24 hours vs. >24 hours: proportion of patients with neurological improvement of ≥1 ASIA AIS grades in patients with complete (A) SCI: 20/59 (33%) vs. 14/46 (30%) (7 studies, types NR; pooled estimates NR)<br>- <24 hours vs. >24 hours: proportion of

SR, Search dates, Search databases	Purpose	Interventions	Primary Outcomes	Evidence Base	AMSTAR-2 rating	Primary Conclusions
						patients with neurological improvement of ≥1 ASIA AIS grades in patients with incomplete (B,C,D) SCI: 217/386 (56%) vs. 201/445 (45%) (9 cohorts, types NR; pooled estimates NR)<br><br><u>Function</u><br>NR<br><br><u>Administrative</u><br>NR<br><br><u>Safety</u><br>NR
Lee 2018<br><br>Database inception through May 2016<br><br>MEDLINE, Embase, CENTRAL, Web of Science, SCOPUS	Investigate neurological outcomes by timing of surgery	<8 hours vs. >8 hours	<u>Efficacy</u><br>Difference in neurological improvement rate (ASIA AIS)<br><br><u>Function</u><br>NR<br><br><u>Administrative</u><br>Difference in LoHS<br><br><u>Safety</u><br>Difference in complications	7 cohort studies (650 patients)<br><br>Unclear if studies adjusted for baseline characteristics<br><br>RoB: Newcastle-Ottawa Quality Assessment Scale	Low	<u>Efficacy</u><br>- <8 hours vs. >8 hours: difference in neurological improvement (2 prospective cohorts, 2 retrospective cohorts; OR = 1.77, 95% CI = 1.24 to 2.52)<br><br><u>Function</u><br>NR<br><br><u>Administrative</u><br>- <8 hours vs. >8 hours: difference in LoHS (2 prospective cohorts, 1 retrospective cohort; WMD = -12.77, 95% CI = -18.52 to -7.02)<br><br><u>Safety</u><br>- <8 hours vs. >8 hours: difference in perioperative complications (3 prospective cohorts, 2 retrospective cohorts; OR =

SR, Search dates, Search databases	Purpose	Interventions	Primary Outcomes	Evidence Base	AMSTAR-2 rating	Primary Conclusions
						0.95, 95% CI = 0.35 to 2.61)
Ma 2020<br><br>Database inception through December 2019<br><br>Embase, MEDLINE, Cochrane library, PubMed	Investigate neurological outcomes by timing of surgery	<8 hours vs. >8 hours	Efficacy<br>Improvement in ASIA AIS ≥1 grade<br><br>Function<br>NR<br><br>Administrative<br>Difference in LoHS<br><br>Safety<br>Difference in perioperative complications	7 cohort studies, 2 RCTs (716 patients)<br><br>Not all studies adjusted for baseline characteristics<br><br>RoB: Newcastle-Ottawa Quality Assessment Scale	Low	Efficacy<br>- <8 hours vs. >8 hours: unadjusted improvement of ASIA AIS ≥1 grade (1 prospective cohort, 3 retrospective cohort, 2 RCT; MD = 0.74, 95% CI = 0.56 to 0.99)<br>- <8 hours vs. >8 hours: improvement of ASIA AIS ≥1 grade in patients with complete (A) SCI (1 prospective cohort, 5 retrospective cohort, 1 RCT; RR = 3.96, 95% CI = 2.02 to 7.76)<br>- <8 hours vs. >8 hours: improvement of ASIA AIS ≥1 grade in patients with incomplete (B, C, D) SCI (1 prospective cohort, 4 retrospective cohorts, 1 RCT; RR = 1.41, 95% CI = 0.95 to 2.10)<br><br>Function<br>NR<br><br>Administrative<br>- <8 hours vs. >8 hours: difference in LoHS (1 prospective cohort, 2 retrospective cohorts, 1 RCT; MD = 0.34, 95% CI = 0.24 to 0.92)<br><br>Safety<br>- <8 hours vs >8 hours: difference in perioperative complications (1 prospective

SR, Search dates, Search databases	Purpose	Interventions	Primary Outcomes	Evidence Base	AMSTAR-2 rating	Primary Conclusions
						cohort, 4 retrospective cohorts, 2 RCTs; RR = 0.92, 95% CI = 0.70 to 1.22)

AMSTAR = A Measurement Tool to Assess Systematic Reviews; ASIA = American Spinal Cord Injury Association; CI = confidence interval; FIM = Functional independence measure; ICU = intensive care unity; LoE = Level-of-evidence; LoHS = length of hospital stay; JOA = Japanese Orthopedic Association; MD = mean difference; RCT = randomized control trial; SD = standard deviation; SR = systematic review' TCCS = traumatic central cord syndrome; WMD = weighted mean difference.

* It is unclear which measure was used to determine neurological improvement.

Appendix G. Forest plots of meta-analyses for key questions

Figure G1. AIS improvement ≥1 AIS grade: Early surgery (≤ 24 hours) vs. late (>24 hours) [KQ1]

			Early		Later			Risk Ratio
Study or Subgroup	Level	% Complete	Events	Total	Events	Total	Weight	PL Estimate [95% CI]
6 Months								
Lenehan 2010	Central Cord	0%	13	17	26	56	17.3%	1.65 [1.12, 2.42]
Fehlings 2012	Cervical	32%	74	131	45	91	39.2%	1.14 [0.88, 1.48]
Sewell 2018	Cervical	29%	19	40	27	55	14.4%	0.97 [0.63, 1.48]
Umerani 2014	Cervical	37%	18	31	25	61	14.3%	1.42 [0.93, 2.17]
Bourassa-Moreau 2016	Mixed	100%	13	38	2	15	1.4%	2.57 [0.66, 10.03]
Wilson 2012*	Mixed	42%	9	22	10	33	5.0%	1.35 [0.66, 2.78]
Ter Wengel 2022	Mixed	67%	51	82	7	14	8.5%	1.24 [0.72, 2.16]
**Subtotal (95% CI)**				**361**		**325**	**100.0%**	**1.26 [1.07, 3.26]**
Total events			197		142			
Heterogeneity: Tau² = 0.00; Chi² = 5.30, df = 6 (P = 0.51); I² = 0%								
Test for overall effect: Z = 2.81 (P = 0.005)								
12 Months								
Lenehan 2010	Central Cord	0%	10	17	18	56	9.1%	1.83 [1.06, 3.17]
Badhiwala 2021	Cervical (83%)	0%	116	528	163	1020	21.5%	1.37 [1.11, 1.70]
Qadir 2020	Thoracolumbar	60%	80	144	45	77	20.3%	0.95 [0.75, 1.21]
Haghnegahdar 2020	Thoracic/TL†	56%	17	37	12	36	8.5%	1.38 [0.77, 2.46]
Du 2018	Mixed‡	0%	170	335	158	386	24.0%	1.24 [1.06, 1.45]
Aarabi 2020	Cervical36	38%	36	57	12	15	16.5%	0.79 [0.57, 1.09]
**Subtotal (95% CI)**				**1118**		**1590**	**100.0%**	**1.17 [0.95, 1.43]**
Total events			429		408			
Heterogeneity: Tau² = 0.04; Chi² = 14.97, df = 5 (P = 0.01); I² = 67%								
Test for overall effect: Z = 1.49 (P = 0.14)								

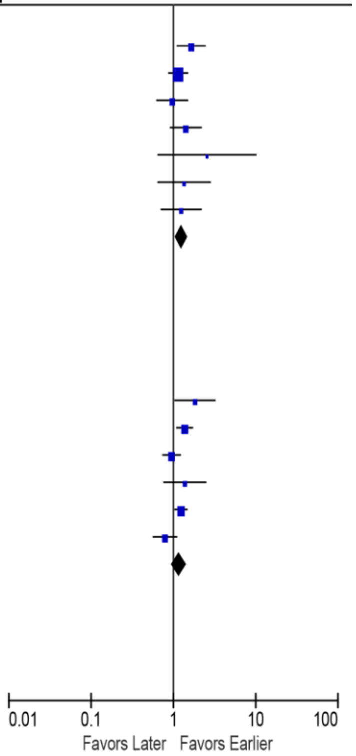

CI = confidence interval; F/U = follow-up; PL = profile-likelihood

* Timing from preoperative to inpatient rehabilitation, mean 89.6 ± 47.4 days

† TL = thoraco-lumbar; >80% had TL injuries

‡ 58% of population had thoracic, 42% had thoracolumbar SCI.

Figure G2. Any complication: Early surgery (≤ 24 hours) vs. late (>24 hours) [KQ2]

Study or Subgroup	Level	F/U(months)	Early Events	Early Total	Later Events	Later Total	Weight	Risk Ratio PL Estimate [95% CI]
**Any Complication**								
Sewell 2018	Cervical	6	17	40	29	55	18.4%	0.81 [0.52, 1.25]
Bourassa-Moreau 2013	Mixed	NR	37	90	109	231	44.5%	0.87 [0.66, 1.16]
Ter Wengel 2022	Mixed	6	49	82	9	14	19.3%	0.93 [0.61, 1.43]
Du 2018	Mixed	12	29	331	43	380	17.7%	0.77 [0.50, 1.21]
**Subtotal (95% CI)**				**543**		**680**	**100.0%**	**0.85 [0.70, 1.03]**
**Total events**			**132**		**190**			

Heterogeneity: Tau² = 0.00; Chi² = 0.44, df = 3 (P = 0.93); I² = 0%

Test for overall effect: Z = 1.67 (P = 0.09)

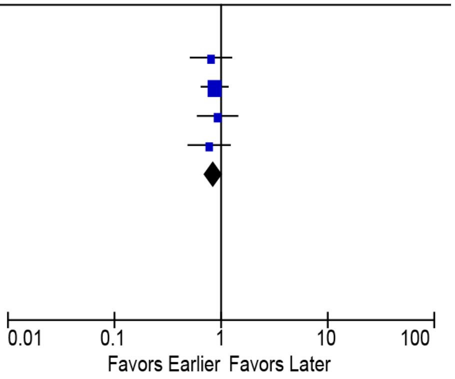

CI = confidence interval; F/U = follow-up; PL = profile-likelihood.

Figure G3. Complete/Incomplete: AIS improvement ≥1 AIS grade: Early surgery (≤ 24 hours) vs. late (>24 hours) [KQ4]

Study or Subgroup	Level	F/U(months)	Early Events	Early Total	Later Events	Later Total	Weight	Risk Ratio PL Estimate [95% CI]
**Complete SCI**								
Wilson 2012*	Mixed	3	5	11	0	12	2.4%	11.92 [0.73, 193.38]
Bourassa-Moreau 2016	Mixed	5	13	38	2	15	8.5%	2.57 [0.66, 10.03]
Fehlings 2012	Cervical	6	19	44	10	27	24.4%	1.17 [0.64, 2.12]
Umerani 2014	Cervical	6	5	11	4	18	12.2%	2.05 [0.69, 6.02]
Aarabi 2020	Cervical	≥6	13	24	2	3	16.0%	0.81 [0.34, 1.96]
Qadir 2020	Thoracolumbar	12	37	86	21	46	32.4%	0.94 [0.63, 1.40]
Haghnegahdar 2020	Thoracic/TL†	12	5	21	1	20	4.2%	4.76 [0.61, 37.28]
**Subtotal (95% CI)**				**235**		**141**	**100.0%**	**1.14 [0.85, 2.09]**
Total events			97		40			
Heterogeneity: Tau² = 0.12; Chi² = 9.59, df = 6 (P = 0.14); I² = 37%								
Test for overall effect: Z = 1.22 (P = 0.22)								
**Incomplete SCI**								
Wilson 2012*	Mixed	3	4	11	10	21	2.2%	0.76 [0.31, 1.88]
Fehlings 2012	Cervical	6	55	87	35	64	17.1%	1.16 [0.88, 1.52]
Umerani 2014	Cervical	169	13	19	19	39	8.1%	1.40 [0.90, 2.19]
Aarabi 2020	Cervical	≥6	23	33	10	12	12.6%	0.84 [0.60, 1.17]
Qadir 2020	Thoracolumbar	12	43	58	24	31	20.1%	0.96 [0.75, 1.22]
Haghnegahdar 2020	Thoracic/TL†	12	12	16	11	16	8.4%	1.09 [0.71, 1.69]
Du 2018	Mixed‡	12	170	335	158	386	31.4%	1.24 [1.06, 1.45]
**Subtotal (95% CI)**				**569**		**569**	**100.0%**	**1.09 [0.95, 1.25]**
Total events			320		267			
Heterogeneity: Tau² = 0.00; Chi² = 5.00, df = 6 (P = 0.54); I² = 0%								
Test for overall effect: Z = 2.47 (P = 0.01)								

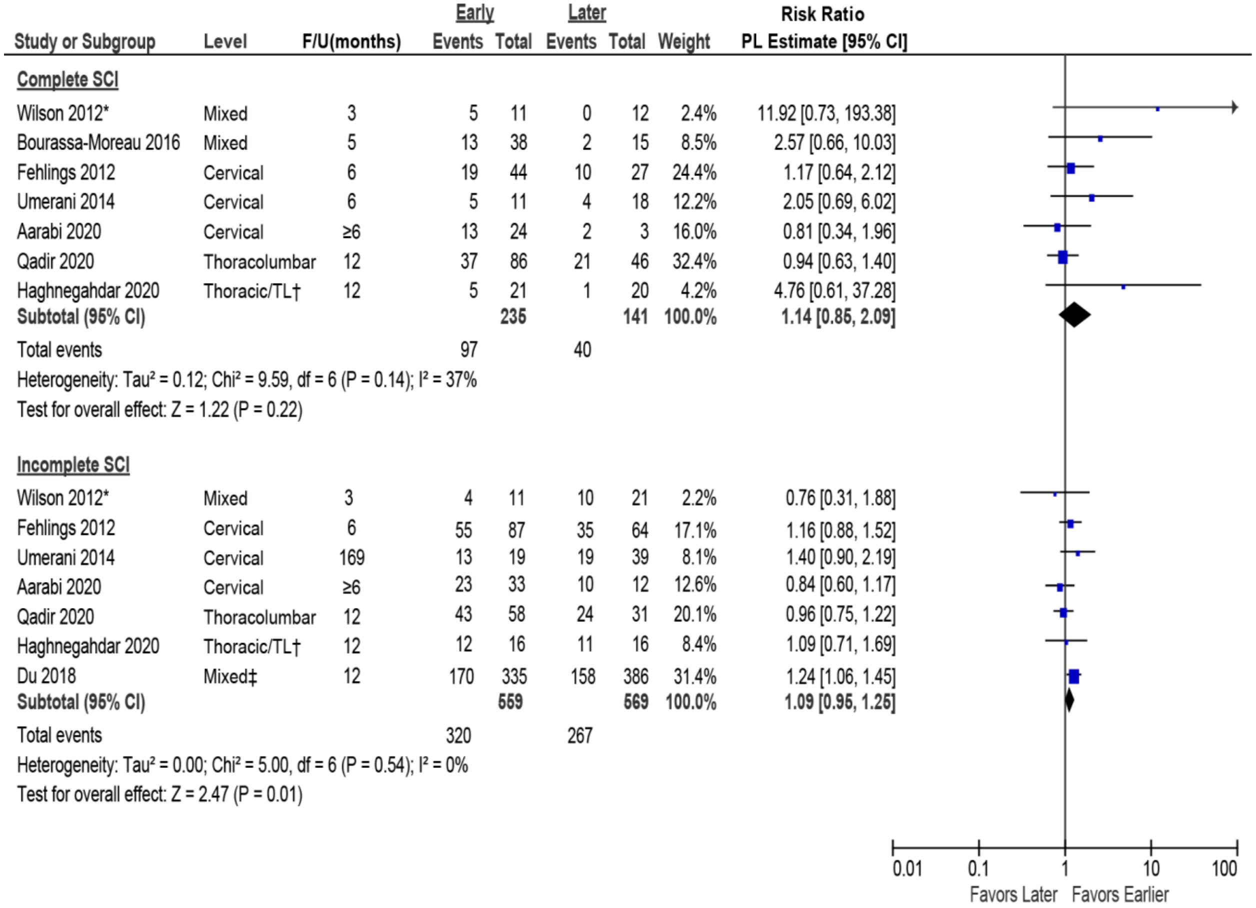

AIS = Asia Impairment Scale; CI = confidence interval; F/U = follow-up; PL = profile-likelihood; SCI = spinal cord injury.
* Timing from preoperative to inpatient rehabilitation, mean 89.6 ± 47.4 days
† TL = thoraco-lumbar; >80% had TL injuries
‡ 58% of population was thoracic, 42% had thoracolumbar.

Figure G4. Levels: AIS improvement ≥1 AIS grade: Early surgery (≤ 24 hours) vs. late (>24 hours) [KQ4]

Study or Subgroup	F/U(months)	% Complete	Early Events	Early Total	Later Events	Later Total	Weight	Risk Ratio PL Estimate [95% CI]
**Cervical**								
Sewell 2018	6	29%	19	40	27	55	19.9%	0.97 [0.63, 1.48]
Umerani 2014	6	37%	18	31	25	61	19.8%	1.42 [0.93, 2.17]
Bourassa-Moreau 2016	5	100%	9	14	0	6	1.0%	8.87 [0.60, 131.75]
Aarabi 2020	≥6	38%	36	57	12	15	24.7%	0.79 [0.57, 1.09]
Badhiwala 2021	12	50%	281	528	444	1020	34.5%	1.22 [1.10, 1.36]
**Subtotal (95% CI)**				**670**		**1157**	**100.0%**	**1.09 [0.80, 1.44]**
Total events			363		508			
Heterogeneity: Tau² = 0.06; Chi² = 12.95, df = 5 (P = 0.02); I² = 61%								
Test for overall effect: Z = 0.90 (P = 0.37)								
**Thoracolumbar**								
Bourassa-Moreau 2016	5	100%	4	20	2	9	2.5%	0.90 [0.20, 4.05]
Qadir 2020	23	60%	80	144	45	77	97.5%	0.95 [0.75, 1.21]
**Subtotal (95% CI)**				**164**		**86**	**100.0%**	**0.99 [0.61, 1.45]**
Total events			84		47			
Heterogeneity: Tau² = 0.00; Chi² = 0.01, df = 1 (P = 0.94); I² = 0%								
Test for overall effect: Z = 0.43 (P = 0.67)								
**Mixed**								
Wilson 2012*	3	42%	7	33	9	49	1.7%	1.15 [0.48, 2.79]
Du 2018†	12	0%	170	335	158	386	95.2%	1.24 [1.06, 1.45]
Ter Wengel 2022	NR	68%	24	49	1	10	3.1%	4.90 [0.75, 32.14]
**Subtotal (95% CI)**				**417**		**445**	**100.0%**	**1.30 [1.08, 1.51]**
Total events			201		168			
Heterogeneity: Tau² = 0.00; Chi² = 0.02, df = 1 (P = 0.88); I² = 0%								
Test for overall effect: Z = 2.65 (P = 0.008)								
**Thoracic/TL‡**								
Haghnegahdar 2020	12	56%	17	37	12	36	100.0%	1.38 [0.77, 2.46]
**Subtotal (95% CI)**				**37**		**36**	**100.0%**	**1.38 [0.77, 2.46]**
Total events			17		12			
Heterogeneity: Not applicable								
Test for overall effect: Z = 1.09 (P = 0.28)								
**Central Cord**								
Lenehan 2010	6	0%	12	17	24	56	53.6%	1.65 [1.07, 2.53]
Lenehan 2010	12	0%	11	17	24	56	46.4%	1.51 [0.95, 2.40]

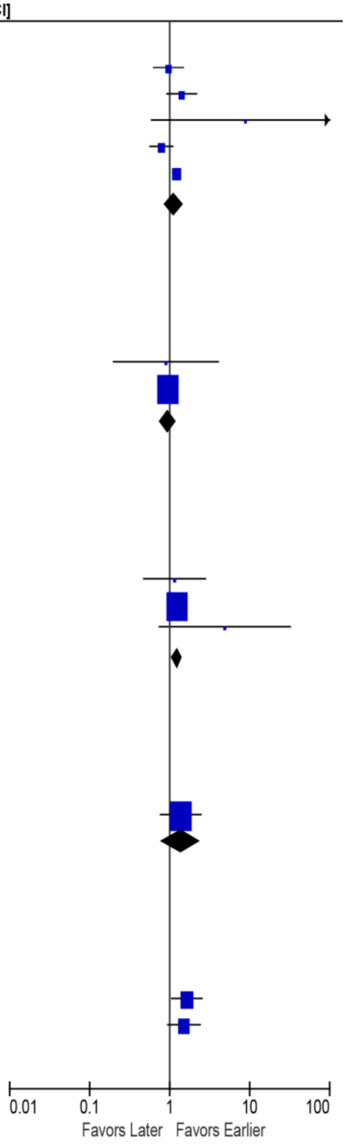

AIS = Asia Impairment Scale; CI = confidence interval; F/U = follow-up; PL = profile-likelihood; SCI = spinal cord injury.
* Timing from preoperative to inpatient rehabilitation, mean 89.6 ± 47.4 days
† 58% of population was thoracic, 42% had thoracolumbar.
‡ TL = thoraco-lumbar; >80% had TL injuries.

Appendix H. Algorithm for classifying adverse events

Categorization of adverse outcomes/harms

Adverse events were often poorly specified or described in studies which is a limitation of the primary studies. Clinical authors were consulted to identify which adverse events would be considered serious. We chose to consider events minor unless they were clearly reported as or likely to be major (i.e., life-threatening or requiring re-operation or invasive intervention).

To categorize events as serious harms/adverse events the following methods were used: For events that were poorly specified or unclear, "Defer to Not serious if unspecified" was used to give a practical and consistent approach to the uncertainty.

Complication	Major (Yes/No)
Mortality	Y
Cardiorespiratory complications	unclear - defer to N if unspecified
Cardiovascular event	unclear - defer to N if unspecified
Construct failure	Y - likely required re-operation
Pressure sores, Decubitus ulcer	unclear - defer to Y because often major
Fixation failure	Y- likely required re-operation
Wound infection (not specified)	unclear - Y for deep or re-operation, N for superficial or no re-operation; defer to N for unspecified
Deep wound infection	Y
Surgical infection (not specified)	unclear, as per above for Wound infection; defer to N for unspecified
Systemic infection, Sepsis	Y
Wound dehiscence	Y
Pulmonary embolism	Y
Deep vein thrombosis	N
Thromboembolic event	unclear - Y for PE, N for DVT; defer to N if unspecified
CSF leak	Y - often requires re-operation or further managment
Gastrointestinal event	unclear - generally N except Y for life-threatening GI bleed; defer to N for unspecified
Neurological deterioration	Y
Meningitis	Y
Revision of surgical screw	Y
Delayed pulled out screw	unclear - Y if required re-operation, defer to N if no re-operation or unspecified
Bilateral rod fracture	Y - likely required re-operation
Methylprednisolone complications	unclear - see comments for sepsis, pneumonia, GI event; defer to N for unspecified
Pneumonia	unclear - Y if associated with any of re-intubation or prolonged intubation or tracheostomy or new ICU admission or sepsis; defer to N if unspecified
Urinary tract infection	N
Tracheostomy required	Y
Unplanned return to operation room	Y
"Other" complications" (not specified)	Unclear - defer to N
Any complications (not specified)	Unclear - defer to N

Appendix I.

Table I1. Criteria for grading the quality of individual studies.

Rating	Description and Criteria
**Good**	• Low risk of bias, most criteria for quality are met and results generally considered valid<br>• Valid methods for selection, inclusion, and treatment allocation; report similar baseline characteristics in different treatment groups; clearly describe attrition and have low attrition; appropriate means for preventing bias and use of appropriate analytic methods
**Fair**	• Some study flaws: May not meet all criteria for good quality, but no flaw is likely to cause major bias that would invalidate results; the study may be missing some information making it difficult to assess limitations and potential problems. This is a broad category; results from studies may or may not be valid.
**Poor**	• Significant flaws that imply biases of various kinds that may invalidate results; most criteria for a good quality study are not met and/or “fatal flaws” in design, analysis or reporting are present; large amounts of missing information; discrepancies in reporting; or serious problems with intervention delivery

Table I2. Description of the strength of evidence grades

Strength of Evidence	Description
**High**	We are very confident that the estimate of risk lies close to the true effect for this outcome. The body of evidence has few or no deficiencies. We believe that the findings are stable, i.e., another study would not change the conclusions.
**Moderate**	We are moderately confident that the estimate of risk lies close to the true effect for this outcome. The body of evidence has some deficiencies. We believe that the findings are likely to be stable, but some doubt remains.
**Low**	We have limited/low confidence that the estimate of risk lies close to the true effect for this outcome. The body of evidence has major or numerous deficiencies (or both). We believe that additional evidence is needed before concluding either that the findings are stable or that the estimate of effect is close to the true effect.
**Very Low**	We have extraordinarily little confidence in the estimate for this outcome. The body of evidence has unacceptable deficiencies.

Appendix References

1. AHRQ Methods for Effective Health Care. In: *Methods Guide for Effectiveness and Comparative Effectiveness Reviews.* Rockville (MD): Agency for Healthcare Research and Quality (US); 2008.
2. Furlan AD, Malmivaara A, Chou R, et al. 2015 Updated Method Guideline for Systematic Reviews in the Cochrane Back and Neck Group. *Spine (Phila Pa 1976).* 2015;40(21):1660-1673.
3. Higgins J, Altman DG. Assessing risk of bias in included studies. In: *Cochrane handbook for systematic reviews of interventions.* Wiley; 2008:187-241.
4. Morton SC, Murad MH, O'Connor E, et al. AHRQ Methods for Effective Health Care Quantitative Synthesis—An Update. In: *Methods Guide for Effectiveness and Comparative Effectiveness Reviews.* Rockville (MD): Agency for Healthcare Research and Quality (US); 2008.
5. Shea BJ, Reeves BC, Wells G, et al. AMSTAR 2: a critical appraisal tool for systematic reviews that include randomised or non-randomised studies of healthcare interventions, or both. *Bmj.* 2017;358:j4008.
6. Dettori JR, Skelly AC, Brodt ED. Critically low confidence in the results produced by spine surgery systematic reviews: an AMSTAR-2 evaluation from 4 spine journals. *Global spine journal.* 2020;10(5):667-673.
7. Dettori JR, Norvell DC. Discordant Systematic Reviews: Which to Believe? *Global Spine J.* 2020;10(2):237-239.
